# Impact of Environmental Airborne Manganese Exposure on Cognitive and Motor Functions in Adults: A Systematic Review and Meta-Analysis

**DOI:** 10.3390/ijerph18084075

**Published:** 2021-04-13

**Authors:** Laura Ruiz-Azcona, Ignacio Fernández-Olmo, Andrea Expósito, Bohdana Markiv, María Paz-Zulueta, Paula Parás-Bravo, Carmen Sarabia-Cobo, Miguel Santibáñez

**Affiliations:** 1 Global Health Research Group, Dpto Enfermería, Universidad de Cantabria, Avda. Valdecilla s/n, 39008 Santander, Cantabria, Spain; laura.ruiz@unican.es; 2 Dpto. de Ingenierías Química y Biomolecular, Universidad de Cantabria, Avda. Los Castros s/n, 39005 Santander, Cantabria, Spain; ignacio.fernandez@unican.es (I.F.-O.); andrea.exposito@unican.es (A.E.); bohdana.markiv@unican.es (B.M.); 3 Economía de la Salud y Gestión de Servicios Sanitarios Research Group, Dpto Enfermería, Universidad de Cantabria, Avda. Valdecilla s/n, 39008 Santander, Cantabria, Spain; maria.paz@unican.es (M.P.-Z.); paula.paras@unican.es (P.P.-B.); 4 Research Health and Bioethics Law Group, GRIDES, IDIVAL, Calle Cardenal Herrera Oria s/n, 39011 Santander, Cantabria, Spain; 5 Cuidados Research Group, Dpto Enfermería, Universidad de Cantabria, Avda. Valdecilla s/n, 39008 Santander, Cantabria, Spain; carmen.sarabia@unican.es; 6 Research Nursing Group, IDIVAL, Calle Cardenal Herrera Oria s/n, 39011 Santander, Cantabria, Spain

**Keywords:** manganese, environmental health, cognitive function, motor function, adults, meta-analysis

## Abstract

Background/Objective: Whether environmental exposure to Manganese (Mn) in adults is associated with poorer results in cognitive and motor function is unclear. We aimed to determine these associations through a meta-analysis of published studies. Methods: A systematic review was conducted to identify epidemiological studies on a population ≥18 years old exposed to environmental airborne Mn, and in which results on specific tests to evaluate cognitive or motor functions were reported. We consulted Medline through PubMed, Web of Science and SCOPUS databases. We also performed a manual search within the list of bibliographic references of the retrieved studies and systematic reviews. To weight Mn effects, a random effects versus fixed effect model was chosen after studying the heterogeneity of each outcome. Results. Eighteen studies met the inclusion criteria. Among them, eleven studies reported data susceptible for meta-analysis through a pooled correlation or a standardized means difference (SMD) approach between exposed and non-exposed groups. Regarding cognitive function, the results of the studies showed heterogeneity among them (I^2^ = 76.49%, *p* < 0.001). The overall effect was a statistically significant negative correlation in the random effects model (pooled r = −0.165; 95%CI: −0.214 to −0.116; *p* < 0.001). For SMD, the results showed a lower heterogeneity with a negative SMD that did not reach statistical significance under the fixed effects model (SMD = −0.052; 95%CI −0.108 to 0.004; *p* = 0.068). Regarding motor function, heterogeneity (I^2^ = 75%) was also observed in the correlation approach with a pooled r (random effect model) = −0.150; 95%CI: −0.219 to −0.079; *p* < 0.001. Moderate heterogeneity was observed according to the SMD approach (I^2^ = 52.28%), with a pooled SMD = −0.136; 95%CI: −0.188 to−0.084; *p* < 0.001, indicating worse motor function in those exposed. Conclusions: Correlation approach results support a negative effect on cognitive and motor functions (the higher the Mn levels, the poorer the scores). Regarding the SMD approach, results also support a worse cognitive and motor functions in those exposed, although only for motor function statistical significance was obtained.

## 1. Introduction

Manganese (Mn) is a trace element, and therefore essential for our normal physiological function [1], being implicated in physiological processes, related to reproduction and development (e.g., formation of healthy cartilage and bone), energy metabolism (e.g., pyruvate carboxylase), urea cycle (e.g., arginase), and antioxidative capacity (e.g., Mn superoxide dismutase) [2]. In contrast, the exposure to high Mn levels described in workers in the Mn industry, such as workers in Mn mines [3], workers employed in plants producing Mn oxides and salts from Mn ore [4], workers employed in alloy production [5,6] or welders [7,8], all with occupational exposure to Mn fumes and/or dusts; has been associated with negative neurological health effects, with a characteristic disorder called “manganism” described, which, although resembling Parkinson’s disease, appears to damage different areas of the brain [9].

With regard to environmental exposure to high levels of Mn, an increasing number of epidemiological studies have been published in recent decades, both in children and in adult populations, focusing mainly on neurological effects on cognitive and motor functions [10]. With respect to children (under 18 years), a systematic review [11] and a meta-analysis [12] have been recently published, in addition to previous ones [13], attempting to select primary studies in which exposure was determined by biomarkers, trying to group the effects into homogeneous neurodevelopmental endpoints. Of the 22 identified relevant studies, only seven reported adjusted associations with the same specific endpoint—child intelligence—using comparable instruments, all but one being cross-sectional, highlighting the need for prospective cohort studies with greater homogeneity regarding endpoints, as well as better control of confounding bias [11]. In the meta-analysis, which included 55 primary studies published until 31 December 2019, the results from their included cohort studies support an association between higher Mn levels and a negative effect on neurodevelopment, especially regarding cognitive and motor skills for children under six years old, as indicated by several metrics, although the need for prospective studies, ideally with repeated measures of exposures, confounders, and outcomes over time, was also highlighted [12]. In the adult population, to our knowledge, there is no published meta-analysis.

Therefore, the objective of this study is to analyze, through a meta-analysis of published primary studies, the impact of environmental Mn exposure on cognitive and motor functions in adults.

## 2. Materials and Methods

### 2.1. Search Strategy and Selection Criteria

**Figure 1:**
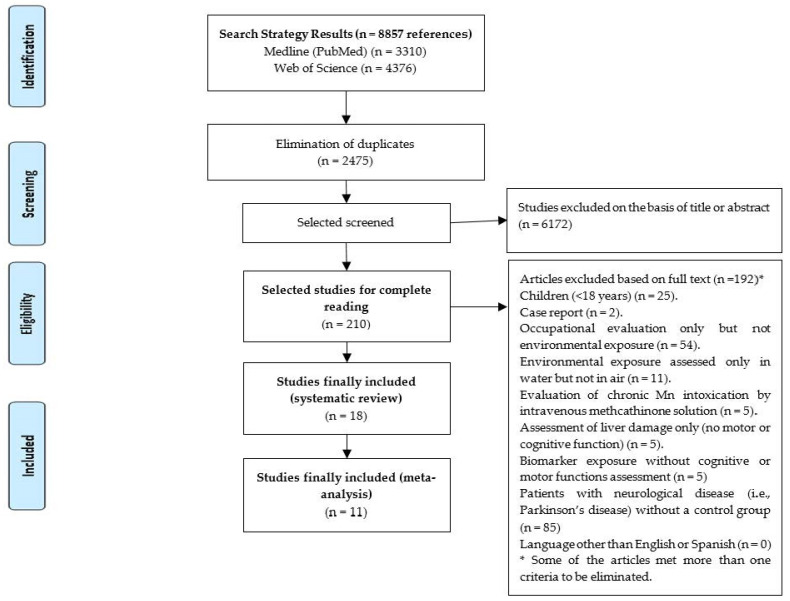
Flowchart used for the identification of original epidemiological articles with Mn environmental exposure determined in air and/or biomarkers in adults (≥18 years), evaluating motor or cognitive function as outcome variables.

A bibliographic search was conducted to identify original epidemiological studies carried out in adults (≥18 years), written in English or Spanish, in which environmental air Mn exposure and/or biomarkers of environmental exposure were assessed, and with at least one determination in cognitive and/or motor functions. We consulted different international bibliographic databases: “Medline through PubMed”, “Web of Science (WOS)” and “Scopus”. We identified all relevant primary studies (published and under publication) until 11th November 2020, by using the strategy: [(MANGANESE OR MN) AND (NEURO* OR COGNIT*) AND ENVIRONMENTAL], using free text and without applying any limitation in the search strategy in PubMed or WOS, and restricting to Title & Abstract in Scopus. We also performed a manual search within the bibliographic references lists of the retrieved studies and systematic reviews. Overall, we found 3310 studies from Medline, 4376 from Web of Science and 1171 from Scopus. Table 1 shows the inclusion and exclusion criteria applied to the references found, either by reading the abstracts or, when necessary, by reading the full text of the primary studies. Figure 1 shows the flowchart used to identify the primary studies to be included in the systematic review and it also reports the reasons for exclusion.

### 2.2. Data Extraction and Quality Assessment

Data generation was performed independently by 2 authors and quality (internal validity and risk of bias) was assessed by using the quality assessment tool for observational cohort and cross-sectional studies from the National Heart, Lung, and Blood Institute (NHLBI), in which 14 criteria are measured to determine an overall quality rating of ‘good’, ‘fair’ or ‘poor’ for each study [14]. Two investigators (MS and LRA) independently evaluated these 14 items of the tool as “Yes”, “No”, “Not Applicable (NA)”, “Cannot Determine (CD)” or “Not Reported (NR)”. Scoring was based on information reported in the manuscript. In addition, an intermediate category of “fair–good” was incorporated. In case of disagreement, consensus was reached among them. Inter-rater agreement between the two investigators who independently scored each manuscript was >95%.

### 2.3. Data Analysis

A meta-analysis was conducted first restricted to studies for which Pearson’s (r) or Spearman’s rho (r_s_) correlations were available. Pooled estimates of the correlation coefficients were calculated by transforming the correlation coefficients into Fisher’s z values. The resulting values were weighted with the inverse of the variance of the correlation coefficients [15]. The 95% confidence intervals of the pooled weighted Fisher’s z values were also calculated, after which all the values were back-transformed to the metric of the Pearson weighted correlation coefficient (pooled r) [16,17].

The standardized mean difference (SMD), with its 95% confidence interval (95%CI), was chosen as a summary measure of the effect to allow us to combine data for the cognitive and motor functions separately. This strategy, which is consistent with the approach taken in other reviews [18,19,20], increases the pool of studies, thereby increasing the power to detect a difference in the motor function both within groups and between groups. Cohen’s criteria were followed to assess effect size (<0.2 = very small effect; ≥0.2 to <0.5 = small effect; ≥0.5 to <0.8 = medium effect; ≥0.8 = large effect) [21]. When both medians and means were reported, standardized differences of medians were also obtained in a sensitivity analysis, by incorporating in the meta-analysis the values of the medians instead of the means.

To weight the Mn effects, a random effects versus fixed effect model was chosen after studying the heterogeneity for each outcome. Statistical heterogeneity was assessed through the Cochran’s Q-test and I^2^ statistic, which describe the percentage of total variation across studies that is attributable to statistical heterogeneity rather than to chance. I^2^ values of 25, 50 and 75% corresponded to low, moderate and high between-study statistical heterogeneity. A *p* value < 0.10 was set as the cut-off point for a statistically significant heterogeneity in the chi squared test for heterogeneity [22]. We used the DerSimonian and Laird random effects model with inverse variance to generate SMDs [23]. The results were grouped separately into cognitive and motor functions. Subgroup analyses were predefined in both groups, attending to the cognitive or motor test used; the domain of cognitive or motor functions analyzed; and according to the type of exposure to Mn assessed.

We sought evidence of publication bias by using the funnel plot method and Egger’s regression asymmetry test [24,25]. In addition, Duval and Tweedie’s “trim and fill” approach was used to obtain the best estimation of the unbiased effect size [17]. The meta-analysis was written following the recommendations of the Preferred Reporting Items for Systematic Reviews and Meta-Analyses (PRISMA) statement [26]. All analyses were conducted by using Comprehensive Meta-Analysis (CMA v2) [27].

Trail Making and Grooved Pegboard Test are based on the time (seconds) required to complete the tests, so the more seconds, the worse function. In the Simple Visual Reaction Time test, the mean is based on milliseconds. Higher scores in the “Unified Parkinson’s Disease Rating Scale (UPDRS)”, Eurythmokinesimetry (EKM) and Postural Balance Testing also denote worse function. In the “Visual Attention Computerized Test, third version (TAVIS-3), both selective and sustained”, the scores are: reaction time, omission errors and commission errors; so longer execution time and greater errors also indicate worse function. This is also the case for the “Coordination Ability Test System (CATSYS) Tremor Pen^®^ “used by Bowler et al., (2016) to determine the Tremor Harmonic index (HI) [28], since abnormal scores are expected to be higher (Danish Product Development Ltd., Snekkersten, Denmark, 2000), so the higher the HI, the worse the motor function [29]. For the rest of the test, the lower scores, the worse function. Thus, if the results for the mentioned tests were shown in the primary articles without correction, normalization or standardization, they were reversed for the pooled analysis. This way, in the CMA v2 meta-analyzed results (and in the text and figures of the results section), a negative correlation denotes that the higher the Mn levels, the worse the cognitive or motor functions, and a negative SMD indicates worse cognitive or motor functions in the group with higher Mn exposure.

## 3. Results

### 3.1. Original Articles That Fulfill Inclusion Criteria for the Systematic Review

According to the selection criteria of our systematic-review, 18 original articles were found [28,30,31,32,33,34,35,36,37,38,39,40,41,42,43,44,45,46]. Table 2 presents the characteristics of these original articles that met inclusion criteria by chronological order of publication, indicating their inclusion or exclusion in the meta-analysis and the section of the meta-analysis in which they are included (cognitive or motor functions, and correlation or SMD). Most of the studies from the same countries were based on the same geographical areas or exposed population. These studies are described in the online supplementary appendix 1.1 specifically. Seven studies [31,32,33,35,44,45,46] presented an odds ratio (OR) or another statistical approach, but they did not present “correlation” or “mean difference data” susceptible of being analyzed in our meta-analysis, so they were included in our systematic review but not in the meta-analysis. These studies are described in the online supplementary appendix 1.2. Therefore, among the 18 original articles, 11 studies presented data finally meta-analyzed in at least one of our analysis strategies. Among these 11 studies included in the meta-analysis, three studies also provided additional results that could not be meta-analyzed [36,39,43]. These studies and their complementary results are specifically detailed in online supplementary appendix 1.3.

Tables S1 and S2 present the results in the NHLBI Quality Assessment Tool for Observational Cohort and Cross-Sectional Studies, for the 11 included and the 7 non-included studies, respectively. All the studies were cross-sectional. This is, with the cognitive or motor functions assessed at the same time as exposure, without a follow up period. Most of the studies evaluating the association between Mn exposure and poorer cognitive and/or motor function attempted to control confounding bias by using multivariate regression analysis, with the exception of four [28,38,39,43]. In the Kornblith et al. study, the control of confounding by using multivariate analysis was not applicable since a two-step cluster data analysis strategy was performed [45].

### 3.2. Meta-Analysis Results

#### 3.2.1. Cognitive Function Correlation

Only four articles showed cognitive function data subsidiary to be analyzed through a correlation coefficient in the meta-analysis [39,42,43,44]. These three studies provided 56 determinations, corresponding to 11 different tests (Auditory Consonant Trigrams; Corsi Block-Tapping Task; Mini-Mental State Examination; Montreal Cognitive Assessment; NAB; RAVLT; Rey Osterrieth Complex Figure test (ROCF); Stroop Color Word test; TAVIS-3; Trail Making Test; WAIS III) (see Table 3).

**Figure 2:**
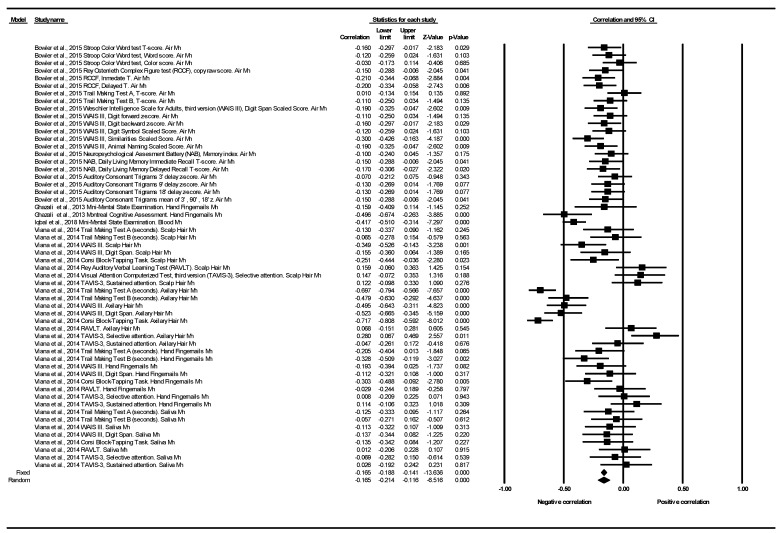
Correlation between cognitive function and exposure to Mn. All tests and all exposures to Mn. Note: a negative correlation indicates that the higher the Mn levels, the worse the cognitive function.

Figure 2 shows the correlation for all cognitive function tests and all exposures to Mn in each study. The article by Bowler et al. (2015) provided 21 determinations corresponding to six different tests, in a combined population belonging to two towns (Marietta and East Liverpool, OH, USA) both highly exposed to environmental Mn from industrial sources [42]. Twenty out of their 21 determinations showed negative correlations in a range between −0.03 and −0.21. One determination was null with a Spearman’s correlation coefficient (r_s_ = 0.01) corresponding to the “Trail Making Test A, T-score” [42]. The article published by Ghazali et al. (2013) in a Malaysian population, provided one determination for each of the two tests used. The determination corresponding to the MMSE scale showed a negative correlation based on Pearson’s correlation coefficient (r = −0.159). The MoCA scale also showed a negative correlation (r = −0.496) [39]. The article published by Iqbal et al. (2018) showed a negative correlation in the MMSE (r = −0.417) in 183 patients diagnosed with cognitive impairment from Pakistan [44]. Finally, the study published by Viana et al. (2014) provided 32 determinations corresponding to five different tests, in a healthy population (without cognitive impairment), from two communities of the town of Simões Filho, Bahia, Brazil: Cotegipe and Santa Luzia villages [42]. These communities are situated at a distance of approximately 1.5 and 2.5 km, respectively, from a ferroMn alloy plant. Twenty-three out of the 32 determinations showed negative correlations in a range between −0.03 and −0.72. Two determinations were null with a correlation coefficient = 0.01, in relation to TAVIS-3 and RAVLT tests in Hand Fingernails and Saliva Mn, respectively. Finally, seven determinations showed positive correlations in a range between +0.03 and +0.28, in relation to these two tests (TAVIS-3 and RAVLT), in Hand Fingernails Mn, Scalp Hair Mn and Axillary Hair Mn (at higher levels, better cognitive function) (see Figure 2). The individual results of the studies presented a high heterogeneity between them (Q = 233.92, df = 55, *p* < 0.001, I^2^ = 76.49%, Tau = 0.027). The overall effect was that of a statistically significant negative correlation in the random effects model, pooled r: −0.165; 95%CI (−0.214 to −0.116), *p* < 0.001 (See Table 3 and Figure 2).

As for the subgroup analysis, only two tests (RAVLT and TAVIS-3) out of the 11 different tests meta-analyzed did not correlate negatively. Depending on the domain of cognitive function analyzed, negative correlations were obtained in all domains, reaching statistical significance in seven of the nine domains studied. Depending on the type of exposure to Mn, all exposure matrices were equally associated with negative correlations (See Table 3 and Tables S3 and S4 and Figures S1–S3).

#### 3.2.2. Cognitive Function SMD between Groups

Only three articles showed cognitive function data subsidiary to be analyzed through a SMD between groups in the meta-analysis [37,40,41]. These three articles together provided 26 determinations corresponding to nine different tests: the Corsi Block-Tapping Test; CPM; MMSE; RAVLT; Story Recall Test; TAVIS-3; TMT; UPDRS; WAIS III (see Table 4).

**Figure 3:**
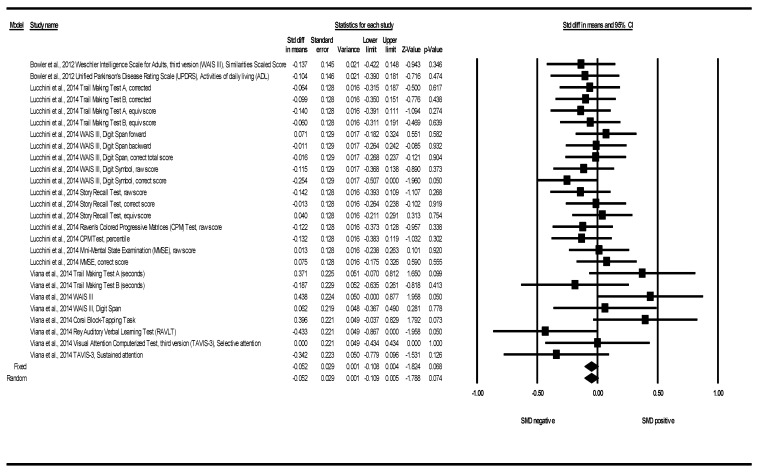
Cognitive function. Standardized mean differences (SMDs) between groups. All tests and all exposures to Mn. Note: a negative SMD indicates that the group with higher Mn levels had worse cognitive function on average.

Figure 3 shows the SMDs for all tests and all exposures to Mn. Bowler et al. (2012) provided two determinations corresponding to two tests (UDPRS, WAIS III) [37]. The article by Lucchini et al. (2014) contributed with 16 determinations corresponding to five different tests: CPM, MMSE, Story recall, TMT A and B, WAIS III (Digit symbol and Digit Span) [40]. Viana et al. (2014) provided eight determinations corresponding to five tests: Corsi Block-Tapping Task, RAVLT, TAVIS-3, TMT and WAIS III (Digit span) [41]. In 17 of the 26 determinations, negative SMDs were obtained, indicating that the means in the exposed group were lower than in the non-or-less-exposed. In one determination, a null SMD was obtained (TAVIS-3, selective attention), indicating that the mean scores in this cognitive test were similar in both groups. Finally, in eight determinations, those exposed scored higher on average (see Figure 3). The results showed a low statistical heterogeneity among them (Q = 25.67, df = 25, *p* = 0.425, I^2^ = 2.62%, Tau = 0.02). The overall effect was a negative SMD that did not reach statistical significance: SMD under the fixed effects model: −0.052; 95%CI (−0.108 to 0.004); *p* = 0.068 (See Table 4 and Figure 3). In a sensitivity analysis, prioritizing the use of medians versus means (when both medians and means were reported), the results also showed low-moderate heterogeneity among them (Q = 31.59, df = 25, *p* = 0.17, I^2^ = 20.86%, Tau = 0.08). We obtained similar results regarding the standardized differences of medians: SMD under the fixed effects model: −0.041; 95%CI (−0.097 to 0.015); *p* = 0.154. SMD under the random effects model: −0.038; 95%CI (−0.102 to 0.025); *p* = 0.239 (see Figure S4).

The analysis of subgroups according to the cognitive tests shows the high number of different tests used, with very few tests involving determinations from more than one publication. In terms of the domain of cognitive function analyzed, the number of domains with determinations from more than one study increased. In this sense, all the SMDs in each of the meta-analyzed cognitive domains were negative (indicating a worse cognitive function in the domain analyzed in those exposed), except for intelligence, based on a single Viana determination (SMD = 0.438), and attention and working memory (SMD under the fixed effects model = 0.02). In terms of the type of Mn exposure evaluated, each study showed a different Mn evaluation approach with different exposures, so the subgroup analysis by this approach was equivalent to a meta-analysis of the results of each study. The results reported by Viana et al., were the only ones that overall did not report a negative SMD (SMD = 0.038) [41] (See Table 4 and Tables S5 and S6 and Figures S5–S7).

#### 3.2.3. Motor Function Correlation

Only three papers showed motor function data subsidiary to be analyzed through a correlation coefficient in the meta-analysis [27,33,40]. These three articles provided 36 determinations, corresponding to five different tests (CATSYS; Dynamometer; Grooved Pegboard; Finger Tapping; Postural Balance) (see Table 5).

**Figure 4:**
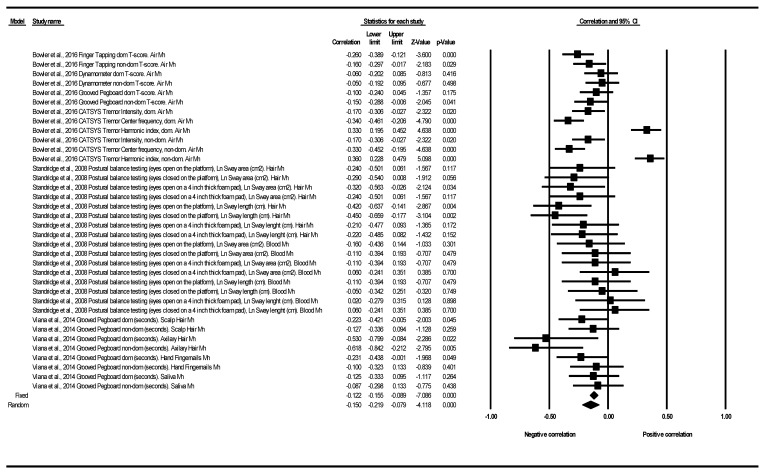
Correlation between cognitive function and exposure to Mn. All tests and all exposures to Mn. Note: a negative correlation indicates that the higher the Mn levels, the worse the motor function.

Figure 4 shows the correlation for all motor function tests and all exposures to Mn in each study. The article published by Bowler et al. (2016) provided 12 determinations corresponding to four different tests (Finger Tapping; Dynamometer; Grooved Pegboard; CATSYS Tremor). Ten out of the 12 determinations showed negative correlations in a range between −0.05 and −0.34. In two determinations corresponding to the CATSYS Tremor HI (dominant and nondominant), positive correlations were obtained (the higher the Mn level, the better the motor function) [28]. The study published by Standridge et al. (2008) provided 16 determinations corresponding to a single test (postural balance testing). Thirteen of the 16 determinations showed negative correlations in a range between −0.05 and −0.45. In three determinations, null or positive correlations of 0.02, 0.06 and 0.06 were obtained [34]. The study published by Viana et al. (2014) provided eight determinations corresponding to the Grooved Pegboard Test (measured in seconds). All the determinations showed negative correlations in a range between −0.09 and −0.62 [41] (see Figure 4). The results showed heterogeneity among them (Q = 139.97, df = 35, *p* = < 0.001, I^2^ = 75.00%, Tau = 0.181). The global effect was that of a statistically significant negative correlation (pooled r under random effect model: −0.150; 95%CI (−0.219 to −0.079); *p* < 0.001 (see Table 5 and Figure 4).

With regard to the subgroup analysis, all the meta-analyzed correlations (in terms of the motor test used, the domain analyzed, and the type of Mn exposure evaluated), were negative. Heterogeneity was very low or null between the different determinations, with the only exception of two determinations between airborne Mn exposure and the results in CATSYS in the section of Tremor HI (dominant and nondominant), presented in the study by Bowler et al. (2016) [28] (See Table 5 and Tables S7 and S8 and Figures S8–S10).

#### 3.2.4. Motor Function SMD between Groups

Seven articles showed motor function data subsidiary to be analyzed through a SMD between groups in the meta-analysis [30,34,36,37,39,40,41]. These articles provided 71 determinations corresponding to 11 different tests: CATSYS, Dynamometer, EKM, Finger Tapping, Grooved Pegboard, Luria Nebraska, Postural balance test, Purdue Pegboard, Simple Visual Reaction Time, Sniffin’ sticks, UPDRS (see Table 6).

**Figure 5:**
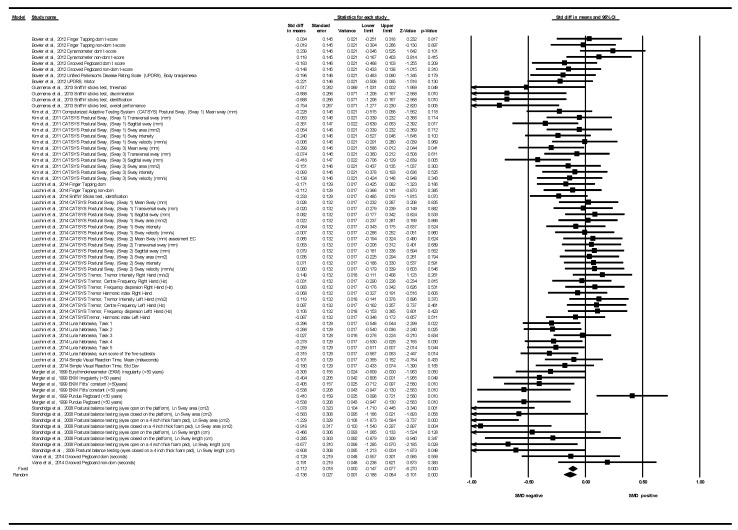
Motor function. Standardized mean differences (SMDs) between groups. All tests and all exposures to Mn. Note: a negative SMD indicates that the group with higher Mn levels had worse motor function on average.

Figure 5 shows the SMDs for all tests and all exposures to Mn. The study published by Bowler et al. (2012) provided eight determinations corresponding to four tests [Hand dynamometer, Finger Tapping, Grooved Pegboard, PDRS (bradykinesia and motor part)] [37]. The article by Guarneros et al. (2013) contributed with four determinations corresponding to a single test (Sniffin’ Sticks Test) [39]. The article by Kim et al. (2011) provided four determinations corresponding to a single test (CATSYS system) [36]. The article by Lucchini et al. (2014) provided 31 determinations corresponding to five different tests (CATSYS, Finger Tapping, Luria Nebraska, Sniffin’ Sticks Test Simple Visual Reaction Time) [40]. The article by Mergler et al. (1999) contributed with six determinations corresponding to two different tests (EKM and Purdue Pegboard) [30] (see Figure 5). The article by Standridge et al. (2008) contributed with seven determinations from a single test (Postural Balance test) [34] (see Figure 5). Finally, the study published by Viana et al. (2014) provided two determinations corresponding to a single test (Grooved Pegboard) [41] (see Figure 5). In 52 of the 71 determinations, negative SMDs were obtained, indicating a lower motor function in those exposed. In four determinations, the SMDs were null (SMD < 0.03 in absolute value), indicating that the average scores in the motor tests were similar in both groups; and in the rest (17 determinations), the SMDs were positive, indicating that the subjects scored higher on average in the motor function tests (better function in the subjects, contrary to the hypothesis) (see Figure 5). The results showed a moderate heterogeneity among them (Q = 146.69, df = 70, *p* < 0.001, I^2^ = 52.28%, Tau = 0.16). The overall effect was a negative SMD that was statistically significant both under the fixed and random effects model: fixed effect SMD: −0.112; 95%CI (−0.147 to −0.077); *p* < 0.001, random effect SMD: −0.136; 95%CI (−0.188 to −0.084); *p* < 0.001 (See Table 6 and Figure 5). In a sensitivity analysis, prioritizing the use of medians versus means (when both medians and means were reported), the results also showed moderate heterogeneity among them (Q = 156.09, df = 70, *p* = 0.001, I^2^ = 55.16%, Tau = 0.16). We obtained similar results regarding the standardized differences of medians: SMD under the fixed effects model: −0.114; 95%CI (−0.149 to −0.079); *p* < 0.001. SMD under the random effects model: −0.140; 95%CI (−0.194 to −0.086); *p* < 0.001 (see Figure S11).

Although all the tests were associated with negative SMDs in the subgroup analysis, two tests stood out in relation to their effect sizes: the “Postural balance testing” with an SMD = −0.715 and the “Sniffin’ sticks test”, with a SMD = −0.50. Depending on the domain of the motor function analyzed, the results in both heterogeneity and effect size practically overlap with those of the test. Subgroup analysis based on the types of exposure would not provide any added interest either, as it would also duplicate the results of the subgroup depending on the type of motor test used (see Table 6 and Tables S9 and S10 and Figures S12–S14).

#### 3.2.5. Publication Bias

**Figure 6:**
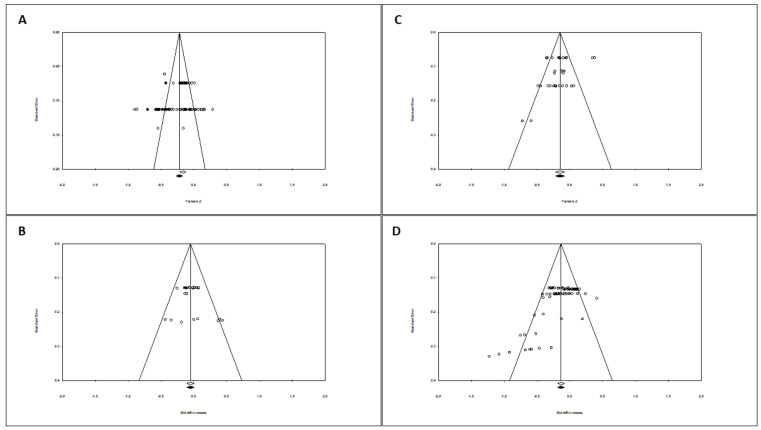
Funnel plot diagrams, with observed and imputed studies after incorporating the Duval and Tweedie (trim and fill) procedure. (**A**) Correlation between cognitive function and exposure to Mn. All tests and all exposures to Mn. Random effect. (**B**) Cognitive function. SMD between groups. All tests and all exposures to Mn. Fixed effect model. (**C**) Correlation between motor function and exposure to Mn. All tests and all exposures to Mn. Random effect. (**D**) Motor function. SMD between groups. All tests and all exposures to Mn. Random effect model.

In terms of publication bias in relation to the correlation between cognitive function and exposure to Mn, the funnel plot was asymmetric. When incorporating the ‘Duval and Tweedie (trim and fill)’ procedure [47], the model includes 13 studies on the left, so the overall effect adjusted by this procedure was slightly different to that observed (see Figure 6A). Therefore, the overall effect under the random effects model went from a negative correlation = −0.165 to increase slightly to −0.229; 95%CI (−0.277 to −0.179). The Egger test marked the intercept value at −0.10; *p*-value (1-tailed) = 0.467; *p*-value (2-tailed) = 0.934.

Regarding publication bias in relation to the cognitive function and exposure to Mn in terms of SMD, the funnel plot was less asymmetric. When incorporating the ‘Duval and Tweedie (trim and fill)’ procedure, no study was included. Therefore, the overall effect adjusted by this procedure was similar to that observed (see Figure 6B). The Egger test marked the intercept value at 1.01; *p*-value (1-tailed) = 0.138; *p*-value (2-tailed) = 0.276.

As regards to publication bias in relation to the correlation between motor function and exposure to Mn, the funnel plot visually presented a slight asymmetry. However, when incorporating the Duval and Tweedie (trim and fill) procedure, no study was included, so the overall effect adjusted by this procedure was similar to that observed (See Figure 6C). The Egger test marked the intercept value at −1.50; *p*-value (1-tailed) = 0.058; *p*-value (2-tailed) = 0.117.

Lastly, regarding publication bias in relation to the 71 motor function determinations in terms of SMD, the funnel plot visually presented a slight asymmetry to the left by the base (in those studies with higher standard error). However, when incorporating the Duval and Tweedie (trim and fill) procedure, no study was included, so the overall effect adjusted by this procedure was similar to that observed (See Figure 6D). The Egger test marked the intercept value at −3.66; *p*-value (1-tailed and 2-tailed) < 0.001.

## 4. Discussion

### 4.1. Cognitive Function Correlation

Our results on cognitive function support a slight but statistically significant negative correlation with environmental airborne exposure to Mn. With concern to the magnitude and interpretation of the correlation coefficient, it should be squared. In this sense, our result (−0.165^2 = 0.0272), indicates that cognitive function and Mn levels share around 2.7% of common variability, and the higher the Mn level, the worse the cognitive function. However, these results are based on only four studies [38,41,42,43], contributing two single studies to 32 and 21 out of the 56 determinations [41,42]. These four studies are very heterogeneous in terms of their study populations. The article by Viana et al. (2014) shows the results in a joint healthy population (without cognitive impairment) from two communities in the city of Simões Filho, Bahia, Brazil. These communities are located at a distance of approximately 1.5 and 2.5 km, respectively, from an industrial ferroalloy source of Mn [41]. The article by Bowler et al. (2015) also shows the results in another population without cognitive impairment, belonging to two other towns (Marietta and East Liverpool, OH, USA), both also highly exposed to environmental Mn from industrial sources, since Marietta is a town near a ferroMn smelter, and East Liverpool is a town adjacent to a facility processing, crushing, screening, and packaging Mn products [42]. In the study published by Ghazali et al. (2013), nail Mn levels were determined in a population from Malaysia in which 35.2% of patients scored below 24 points on the MMSE score and 92.6% scored <26 on the MoCA scale, which correspond to the cut-off points considered as cognitive impairment in both tests respectively. Mn levels in nails were on average = 1.00 µg/g [SD = 0.23], within the established reference range of 0.10 to 1.48 µg/g [38]. Finally, Iqbal et al. (2018) show a negative correlation in the MMSE in 183 participants diagnosed with cognitive impairment, ordinarily classified based on their score and further compared to 90 people without cognitive impairment, all of whom were from Pakistan [43]. Therefore, it is necessary to publish a higher number of studies that provide greater homogeneity in this regard. The clinical impact of these findings must also be evaluated in depth in subsequent prospective studies.

### 4.2. Cognitive Function Standardized Mean Difference (SMD) between Groups

Three articles provided means or medians in the cognitive tests in their respective exposed and non-or-less-exposed populations, so that 26 determinations were subsidiaries of meta-analysis through a SMD [37,40,41]. The meta-analyzed SMD indicates that overall, those exposed scored less on average in the cognitive tests, that is, they had worse results (worse cognitive function), although the effect size was very small (<0.2) based on Cohen’s criteria [21]. Luchini et al. (2014) [40], presented the results as Bowler et al. (2012) [37] of a healthy population (without cognitive impairment) from two regions, one (Valcamonica, Italy) exposed to significantly higher environmental levels than the reference region (Garda Lake, reference area). The study published by Viana et al. (2014) [41] also concerns a healthy population (without cognitive impairment), however as mentioned above it compares two communities at a distance of 1.5 and 2.5 km from the industrial source of Mn, and therefore the difference in exposure is not as pronounced as it could be between the other two studies [37,40]. This could explain why in this study mixed results are presented, with both negative and positive SMDs in the different determinations of the cognitive tests, highlighting the positive SMDs in the WAIS III that produce great heterogeneity between the results of the three studies, in relation to this cognitive test and its corresponding analyzed domains [40]. Despite this very small effect size, the clinical relevance of these results should also be studied in future longer-term prospective research, as the design of the published studies suggests that the greater the difference in exposure between the groups compared, the greater the SMD. In an inverse analysis strategy, Iqbal et al. (2018) [43], in addition to determining correlations, compared the means of blood Mn levels in four groups ordinarily classified based on MMSE scores. Mn levels were significantly higher (*p* < 0.001) in severe (*n* = 25) (MMSE score < 10) (blood Mn levels 92.08 ± 6.8 μg/L) and moderate cognitively impaired group (*n* = 86) (MMSE scores range 10–20) (blood Mn levels 77.8 ± 2.4 μg/L) as compared to the age-matched healthy control group (*n* = 90) (MMSE scores ranged 25–30) (blood Mn levels 52.8 ± 2.8 μg/L). The mildly (*n* = 72) (MMSE scores range 21–24) (blood Mn levels 64.97± 3.76 μg/L) cognitively impaired group also had significantly elevated levels of Mn compared to the age-matched healthy control group (*p* < 0.05). As they did not report MMSE scores for exposed and unexposed Mn, these results could not be included in our SMD strategy, but would clearly support a negative association in relation to cognitive function. The results for blood Mn (above the 75th percentile) from the study published by Santos Burgoa et al. (2001) and for air Mn (above the 0.1 cut-off point μg/m^3^,) from the study published by Solís Vivanco et al. (2009), reporting OR > 1 for worse scores on their cognitive tests analyzed, would indirectly support these results [32,35]. Lastly, although their results could not be included in the meta-analysis either, the article published by Rafiee et al. (2019) with healthy Iranian volunteers, reported 0.201 and 0.204 more seconds in the TMT-A and B respectively for each increase of 1 µg/g hair Mn, also supporting this negative association (the higher levels, the worse cognitive function) [46].

### 4.3. Motor Function Correlation

In terms of motor function our results also support the existence of a statistically significant negative correlation of a minor magnitude, based in this case on 36 determinations from five tests reported in three articles [28,34,41]. Thus, motor function and Mn levels share around 2% of common variability, and similarly, at higher Mn levels, lower scores in motor function tests. These three studies are more homogeneous, at least in terms of their study populations (all without cognitive impairment). The study published by Viana et al. (2014), in healthy volunteers from Simões Filho, Brazil, reported motor correlation results for the Grooved Pegboard (determined in seconds) [41]. The study by Standridge et al. (2008) is also located in Marietta, Ohio, US (same area as the studies by Bowler et al.) but includes different healthy volunteers. The test used (Postural Balance testing) is also different from those reported in the Bowler et al. studies [34]. The study population in Bowler et al. (2016) is the same as the study published a year before (Bowler et al., 2015), that is, it is the same joint population without cognitive impairment belonging to the two populations highly exposed to environmental Mn from industrial sources in Ohio, US [27,41]. Finally, in the publications by Kim et al. (2011) (CATSYS 2000) and Guarneros et al. (2013) (Sniffin’ sticks test), the specific correlation data were not shown but they did specify nonsignificant negative correlations in favor of the meta-analysis results [36,39]. As in the case of cognitive function, further studies are needed to evaluate the clinical impact of these findings.

### 4.4. Motor Function Standardized Mean Difference (SMD) between Groups

We found seven articles providing 71 determinations corresponding to eleven different tests, supporting a worse motor function in those with higher environmental exposure to Mn, but also of a very small effect size based on Cohen’s criteria [21]. The study of Guarneros et al. (2014) only assessed olfactory function through the “Sniffin’ sticks test” [39]. Olfactory function could be interpreted as a neurological function independently of cognitive or motor functions [10]. However, due to the latest evidence suggesting that among older adults, olfactory function is associated with mobility, balance, fine motor function, and manual dexterity, and with challenging upper and lower extremity motor function tasks (independent of cognitive function) [48], we decided to include it in the SMD motor function meta-analysis. As the results of this study provided a negative SMD of almost moderate magnitude based on Cohen’s criteria, and also were associated with some heterogeneity, we performed a sensitivity analysis excluding it, and excluding also the determination in the “Sniffin’ sticks test” provided by Lucchini et al. (2014) [40]. This sensitivity analysis showed similar results regarding overall heterogeneity and effect, with a negative SMD that was also statistically significant both under the fixed and random effects model. Lastly, the results for air Mn from the study published by Rodríguez-Agudelo et al. (2006) in the form of OR > 1 for worse scores in their analyzed motor tests, would indirectly support these results [33].

### 4.5. Methodological Issues Relating to Meta-Analysis

One of the main limitations of meta-analysis is the presence of a publication bias [25]. In order to minimize this bias, systematic searches were carried out in the main bibliographic databases, and from the references of the identified studies. In addition, the search strategy was complemented by specific searches of grey literature. Due to our systematic bibliographic search strategy, the omission of published studies seems unlikely. No studies were excluded due to the language of publication.

Regarding the publication bias in the correlation between cognitive function and exposure to Mn, the funnel plot visually presented asymmetry, including studies on the left when incorporating the Duval and Tweedie (trim and fill) procedure, which however had little impact on the overall adjusted effect [47]. The Egger test marked the intercept value at −0.10 with a nonsignificant p. This lack of agreement between the trim and fill procedure and the *p*-value would not be contradictory, in the sense that while a significant p supports the existence of bias, a nonsignificant p would not rule it out as it can be explained for example by a lack of statistical power. Nevertheless, by including studies from the left, the publication bias, if any, would favor the hypothesis of a larger negative correlation, although the procedure itself in this case shows that the change in the overall adjusted effect is not relevant. Regarding the SMD between groups for cognitive function, the funnel plot visually did not present asymmetry and no study was included when incorporating the Duval and Tweedie (trim and fill) procedure. All this supports the validity of our results, in the sense that there is no publication bias, or at least not in the way that the impact on cognitive function is considerable.

In relation to the motor function and correlation approach, the funnel plot visually presented a slight asymmetry, and the Egger test marked the intercept value at −1.50; with a *p* value of 0.058 in the unilateral contrast test. However, the Duval and Tweedie (trim and fill) procedure, did not incorporate any studies. For the SMD, the asymmetry in the funnel plot was associated with an Egger T test with very significant *p* values (*p* < 0.001), which would support the existence of a bias, but which, however, did not correspond either with the inclusion of any study using the Duval and Tweedie (trim and fill) procedure. The same occurred in the sensitivity analysis by excluding the “Sniffin’ sticks test” determinations. Thus, there would also be no conclusive evidence of the existence of a publication bias in motor function, also supporting the validity of our estimates.

In addition to the possibility of publication bias, another limitation of the present meta-analysis would be the low quality of some primary studies, and the small sample size of most of them. Regarding the design and quality of the primary studies, all studies included in our systematic review had a cross-sectional design, with cognitive or motor functions assessed at the same time as exposure, i.e., in none of the designs there was a follow-up period to assess the impact of short, medium or long-term exposure on motor or cognitive functions. This reinforces the need for prospective studies in adults, with a follow-up period in which the appropriate time sequence between exposure (cause) and effect on cognitive and motor functions should be preserved. In relation to the control of bias such as confounding, two studies attempted to control for this bias in the design phase by matching exposed and unexposed groups for gender and as closely as possible, for age and level of schooling [39]; and cognitive impairment volunteers and healthy controls by age [43]. Seven of the 11 studies tried to control confounding by using multivariate analysis, with a greater or fewer number of covariates included in the model. In four studies, the articles did not reflect any method to control for this bias in the analysis phase [28,38,39,43]. The lack of control for confounding bias both in the design or analysis phase could have an effect on the validity of the primary results and therefore also on the validity of the meta-analysis results.

Regarding the study published by Iqbal et al., in addition to the lack of use of multivariate analysis, it should be noted that the reported blood Mn levels in cognitive impairment volunteers (*n* = 183) and healthy controls (*n* = 90) are particularly high in comparison with those of other studies (means of 92.08; 77.8; 64.97; and 52.8 μg/L for each of the groups respectively), which could be explained by a selection bias (having selected abnormally exposed volunteers) or by a bias in the characterization of the exposure (by a poor validity in the determinations of blood Mn). Overall, this study obtained the lowest quality among analyzed in our quality approach, and it was the only one obtained a grade of “poor”. This study contributed only with a single determination in the meta-analysis section of correlation and cognitive function. By removing this determination in a sensitivity analysis, the results did not change; so it was decided to include it, presenting the overall results of the four studies providing 56 determinations in the meta-analysis.

In relation to the choice of model for meta-analysis, the heterogeneity of the results of the studies in the different analyses was variable, suggesting the choice of a fixed or random effects model for the synthesis of results adapted to the specific heterogeneity for each particular analysis strategy, showing the results under the random effects model in the case of moderate & high heterogeneity (I^2^ > 50%), since under the random effects model the studies providing greater heterogeneity would have a lower relative weight. Nevertheless, due to the a priori sources of heterogeneity identified, it was considered appropriate to additionally show the results under both models, at least in the presented figures, even for those strategies with low heterogeneity.

Lastly, some racial differences in blood Mn levels have been suggested in the US population from the National Health and Nutrition Examination Survey: blood Mn medians of 11.1, 8.53, 9.26, 10.7 and 12.0 µg/L for Mexican Americans, non-Hispanic blacks, non-Hispanic whites, all Hispanics and Asian, respectively [49], and it cannot be ruled out that there may exist some differences in the enzymatic kinetics of Mn detoxification between racial groups as is the case with other substances [50,51]. However, none of the studies included in our review specified Mn levels as a function of race, nor did they report associations between Mn levels and cognitive or motor functions stratifying by race, so a sensitivity analysis as a function of race was not feasible in our meta-analysis.

### 4.6. Exposure to Airborne Manganese in the Context of Health-Derived Guidelines

Several studies included in this systematic review have been conducted in areas near air Mn sources, such as Mn ore mines [39], Mn ore processing plants [28,42], and Mn ferroalloys plants [28,34,36,37,40,41,42]. When air Mn was assessed in these areas by measuring or modelling the PM10 or PM2.5-bound Mn concentration, Mn levels were within the range or slightly above the health-derived guidelines given by different organizations, such as the World Health Organization (WHO), which recommends an annual average guideline of 150 ng Mn/m^3^ [52], the US EPA, which established a reference concentration (RfC) of 50 ng/m^3^ (annual basis) [53], or the Agency for Toxic Substances and Disease Registry (ATSDR), which developed a minimum risk level (MRL) for Mn of 300 ng/m^3^ [51]. Thus, mean modelled Mn values of 180 ng/m^3^ in PM10 and 50 ng/m^3^ in PM2.5, have been reported in Marietta [28,36,37,42], and of 310 ng/m^3^ in PM10 and 30 ng/m^3^ in PM2.5 in East Liverpool, Ohio, US [28,42]. In northern Italy (Valcamonica), mean Mn levels of 26.4 ng/m^3^ have been measured using personal exposure PM10 samplers [40]; and a mean of 151 ng/m^3^ in PM2.5 was measured in Simões Filho (Brazil), the same area considered by Viana et al. (2014) study [41], in an epidemiological study developed in children [54].

At the moment, since no human biomonitoring values derived on the basis of toxicological and epidemiological studies are available for Mn in human matrices commonly used as biomarkers of exposure, such as whole blood, hair, nails or saliva [10], there is a need to regulate the airborne Mn level. In this context, several authors consider that current regulatory air Mn guidelines are extremely conservative because of the potential non-linear biological response to Mn exposure, suggesting limits between 1 to 10 μg/m^3^ [55,56]. The results shown in this meta-analysis regarding the potential effect of environmental Mn exposure on worsening the cognitive and motor functions, would support the need to derive limit or threshold values for this metal in the regulation of different countries around the world, as has been done for other metal(loid)s such as Pb, Ni, Cd, As, or Hg.

## 5. Conclusions

In conclusion, our meta-analysis shows a statistically significant negative correlation between cognitive and motor functions (the higher the Mn levels, the poorer the scores). Regarding the SMD approach, our results also support worse cognitive and motor functions in the exposed, although only for motor function statistical significance was obtained. This would support the need to regulate airborne Mn levels. However, further follow-up studies with a mostly systematic methodology, using a standardized, more homogeneous battery of neurological/neuropsychological tests are needed to deepen the effects derived from environmental airborne Mn exposure and their clinical relevance.

## Figures and Tables

**Table 1:** Inclusion and exclusion criteria.

**Inclusion Criteria**
• Primary epidemiological articles (original papers) in adults (≥18 years). • Mn as a heavy metal. • Environmental exposure determined in air and/or biomarkers. • Assessment of cognitive or motor functions. • Language: written in English or Spanish.
**Exclusion Criteria**
• Case reports or reviews. • Developed in rats, other animals or in human cell models. • Children (<18 years). • Mn referred to the enzyme “Manganese superoxide dismutase” (not as a heavy metal). • Mn as “trace element” or “micronutrient”, evaluating for example its role in a “micronutrient supplementation”. • Occupational evaluation only but not environmental exposure. • Environmental exposure only assessed by ingestion (e.g., water). • Evaluation of chronic Mn intoxication by intravenous methcathinone solution. • Determination of exposure using biomarkers but without assessing cognitive or motor functions. • Assessment of liver damage only (no motor or cognitive function). • Only in patients with neurological disease (i.e., Parkinson’s disease) without a control group.

**Table 2:** Characteristics of studies included in the systematic review by chronological order of publication.

Author/(Year)/Country	Design. Study Population	Exposure	Neurological and Cognitive Tests	Neuromotor Evaluation Test (Motor/Tremor)	Control of Biases. Results
**Mergler et al. (1999)/Canada ^d^** [30]	Cross-sectional. 273 healthy population (without cognitive impairment) from Southwest Quebec, (after removing persons with sequelae of neurological illness and persons with heavy alcohol consumption), where a former Mn alloy production plant existed.	Biomarkers of Mn exposure (Blood). Arithmetic mean was used for categorical analyses between exposed and non-exposed (<7.5 µg/L, >= 7.5 µg/L). Same study as Beuter et al. (1999).	Memory Assessment Scale, Rey-15-item visual memory and reproduction, Wechsler Intelligence Scale for Adults, third version (WAIS III) Digit Span test, Cancellation H, Trail Making Test * (TMT), Stroop color/word test, Near visuality acuity chart, AMTI acusway system.	Eurythmokinesimetry (EKM) *, Purdue Pegboard Test, Diadocchokinesimetry (DIADO), TREMOR system of Danish Product Development (DBP).	Results stratified by age and gender. Confounding controlling by multivariate analysis (educational level, tobacco and alcohol consumption and others). Analyses of the individual measures revealed that people in the higher Mn category performed less well on the pointing task, EKM, manifesting more irregularity and higher Fitt’s constant and a tendency to make more multiple contacts on the target. On DIADO, those in the higher Mn category displayed slower velocity. For the other measures of motor performance, handarm tremor and tapping movements, showed no relation with Mn.
**Beuter et al. (1999)/Canada ^e^** [31]	Cross-sectional. Same study population as Mergler et al. (1999).	Same exposure as Mergler et al. (1999).	Same tests as Mergler et al. (1999), but presenting results with different analysis strategy.	Same tests as Mergler et al. (1999), but presenting results with different analysis strategy.	Confounding controlling by multivariate analysis (age, gender, educational level, tobacco and alcohol consumption and others). Mn exposure was found to be associated with a decrease in ability to perform regular, rapid and precise pointing movements and a decrease in ability to attain high maximum rotation speeds in rapid alternated movement, and an increase in regularity of tremor oscillations.
**Santos Burgoa et al. (2001)/Mexico ^e^** [32]	Cross-sectional. Two communities living within a Mn mining district in central Mexico: Community A (*n* = 44) was 2 km from the primary ore refining plant, residing in the uphill area surrounding the plant. Community B (*n* = 27) was 25 km downhill and downstream from the point source. The name of the communities is not detailed, but probably is the same state as Guarneros et al. (2013); Rodríguez-Agudelo et al. (2006); and Solís Vivanco et al. (2009) studies.	Biomarkers of Mn exposure (Blood). Median Blood Mn 15 μg/L; range (7.5–88). The upper quartile started at 20 μg/L; the upper 10% was above 25 μg/L.	The Hooper visual organization test (HVOT), TMT *, WAIS III Digit span, Animal naming, Mini-Mental State Examination (MMSE).	The neuropsychological scheme (motor behaviour).	Multivariate analysis including most frequently as covariates: age, schooling, community, alcohol; and occasionally age and sex. Mn increased the risk of deficient cognitive performance 11.7 times (Mini-Mental score of less than 17). The models with the highest explanation of the effects are those related to motor strength, coordination, and cognitive performance. The motor test employed was fingertip touching. The most relevant of these are the results of the Mini-Mental Examination. A lack of trend for the Mini-Mental test with increasing Mn concentrations, while the estimated risk ratios for each tertile of Mn for reduced Mini-Mental score, and other tests displaying a U-shaped dose–response curve.
**Rodríguez-Agudelo et al. (2006)/Mexico ^e^** [33]	Cross-sectional. 288 healthy participants (168 women and 120 men) from eight communities at various distances from Mn extraction or processing facilities in the district of Molango (Hidalgo state) were studied. Same district as Santos-Burgoa et al.. (2001) and Guarneros et al. (2013) study, and same study population as Solís Vivanco et al. (2009).	Air Mn evaluation. Range: 0.003 to 5.86 µg/ m^3^. Geometric mean = 0.10 µg/m^3^ (median 0.13). A cut-off point of 0.05 and 0.1 µg/m^3^ was used to dichotomously categorize Mn exposure. Biomarkers of Mn exposure (Blood). Blood Mn range: 5.0 µg/L to 31.0 µg/L (geometric mean: 9.44 µg/L).	_	Ostrosky-Solís’s neuropsychological battery.	Multivariate analysis including as possible covariates alcoholism, gender, age, socioeconomic status, blood lead, and scholarship if *p* < 0.1. Considering cumulative exposure index in quartiles (to have variability below and above the cutting point), there was an association between air Mn concentrations and motor tests that assessed the coordination of two movements: OR = 3.69; 95%CI (0.9 to 15.13) and position changes in hand movements, reaching statistical significance: OR = 3.09; (95%CI 1.07 to 8.92). An association with tests evaluating conflictive reactions (task that explores verbal regulations of movements) was also found: OR = 2.30; 95%CI (1.00 to 5.28). No associations were found between blood Mn and poorer motor tests results.
**Standridge et al. (2008)/United States (US) ^c,d^** [34]	Cross-sectional. Healthy population (*n* = 29 without cognitive impairment) from 19 to 68 years (mean = 50) from Marietta, Ohio (same area as Bowler et al. studies), a town near a ferroMn refinery.	Biomarkers of Mn exposure (Hair and Blood). Mean hair Mn 4.4 μg/g; range (1.2–12.4). Mean blood Mn of 9.4 μg/L; range (4.2–21.7).	_	Postural Balance Testing *	Multivariate analysis including gender, age and height/weight ratio (HT/WT). Caffeine, tobacco and alcohol included if *p* ≤ 0.10. Pearson correlation coefficients between measures of postural balance and natural logarithm (Ln) transformed hair Mn were all positive (the higher levels, the worse motor function) and reached statistical significance for sway length (SL) under eyes open (EO) and eyes closed (EC) on the platform. Following covariate adjustment within the linear regression analysis, Ln hair Mn reached statistical significance with sway area (SA) and SL under EO and EC test conditions.
**Solís Vivanco et al. (2009)/Mexico ^e^** [35]	Cross-sectional. Same study population as Rodríguez-Agudelo et al. 2006 and same district as Guarneros et al. 2013 study, where there are important Mn extraction and processing facilities.	Air Mn evaluation. Biomarkers of Mn exposure (Blood). See Rodríguez-Agudelo et al. 2006 data for details.	MMSE, Digit Span, Word Association Test, Clock Test, Word List test, Semicomplex Desing test.	_	Multivariate analysis including age, education, gender, tobacco and alcohol consumption, and blood Pb concentration. When using the 0.1 μg/m^3^ cut-off point of air Mn, there was a risk of poor performance on the digit span test (attention impairment): OR = 1.75; 95%CI (1.01 to 3.06). When using the 0.05 μg/m^3^ cut-off point there was no risk of poor performance on any test (e.g., OR digit span test = 1.24; 95%CI (0.67 to 2.29). There was no association between blood Mn concentration and cognitive function (e.g., OR MMSE = 1.17, 95%CI (0.99 to 1.38).
**Kim et al. (2011)/US ^d^** [36]	Cross-sectional. Healthy participants (without cognitive impairment) from “Marietta”, Washington County, Ohio (*n* = 100, exposed to high levels of Mn) are compared with “Mount Vernon”, Knox County, Ohio (*n* = 90, non-exposed to high levels of Mn).	Air Mn evaluation. Modeled air Mn (Mn-Air) reported only for Marietta. Mean ±SD, 0.18 µg/m^3^ ± 0.13 µg/m^3^. Median = 0.16 µg/m^3^. Range 0.04–0.96 µg/m^3^. Biomarkers of Mn exposure (Blood). Mean in Blood ±SD = 9.65 µg/L ± 3.21 µg/L. Range: 4.91–24.60 µg/L (Marietta, exposed). Mean ±SD = 9.48 µg/L ± 3.16 µg/L. Range: 3.75–18.90 µg/L (Mount Vernon, non-exposed).	Unified Parkinson’s Disease Rating Scale (UPDRS)-Activities of daily living (ADL) *.	Coordination Ability Test System (CATSYS)*, UPDRS (motor and bradykinesia)*.	Multivariate analysis using different models. Model 1: adjustment for age, sex, ethnicity, smoking, alcohol, educational level, household income, and insurance status. Model 2 and 3 incorporates more covariates in addition. The Mn-exposed group (Marietta) showed significantly higher postural sway scores under eyes-open conditions according to CATSYS assessment than the comparison group (Mount Vernon), but the effect sizes were small to medium (0.23–0.42). The overall means of the UPDRS Motor and Bradykinesia scores were significantly higher in the exposed group than in the comparison group. However, the effect sizes were small (Motor: 0.22; Bradykinesia: 0.20). UPDRS Motor or Bradykinesia scores did not correlate with exposure indices such as Mn-B, or modeled air-Mn (data not shown). The risks of abnormal UPDRS Motor and Bradykinesia scores (scores >0) were in the exposed group respectively 2.43- and 2.90-fold higher than in the comparison group after adjustment for confounding variables.
**Bowler et al. (2012)/US ^b,d^** [37]	Cross-sectional. Same study population as Kim et al. (2011).	Same exposure as Kim et al. (2011).	Medical symptoms questionnaire (MSQ), UPDRS-ADL *, the Symptom Checklist-90-Revised (SCL-90-R), the Environmental Worry Scale (EWS), The Health-Related Quality of Life (HRQOL) Scale, Similarities subtest from WAIS III, The Rey 15-Item Test, Victoria Symptom Validity Test.	Grooved Pegboard *, Grip Strength (Dynamometer), Finger Tapping Test, UPDRS (tremor and motor)*.	Multivariate analysis incorporating age, sex, diabetes, education, health insurance status, and psychiatric medication as a function of the test used as dependent variable. The Mn-exposed participants showed a slightly higher average T score (mean±SD, 54.1±9.0) than comparison participants (51.6±7.0) (*p* = 0.035) with an effect size of 0.308. Scores on two of the UPDRS scales differed; the exposed group had higher levels of bradykinesia (*p* = 0.04) and motor disturbance (*p* = 0.034). However, these effect sizes were small (0.196 and 0.222). WAIS III no significant difference was found (*p* = 0.915) between mean scores of those in the exposed group (10.8±3.1) and those in the comparison group (11.2±2.7). The Finger Tapping scores were dichotomized and 40% had worse function for the dominant hand, and 46% had worse function for the nondominant hand. For the three UPDRS variables, the crude prevalence of impairment ranged from 19% to 53%. There was no statistically significant association detected between generalized anxiety and the two Finger Tapping Tests.
**Ghazali et al. (2013)/Malaysia ^a^** [38]	Cross-sectional. 54 elderlies from Selangor, aged 60 and above. Based on cut-off score of 24 for MMSE and 26 for MoCA, the subjects were considered as having normal cognitive function from MMSE score (64.8% ≥ 24, 35.2% < 24), but found to be cognitively impaired based on MoCA score (7.4% ≥ 26, 92.6% < 26).	Biomarkers of Mn exposure (fingernails). Levels of heavy metals and trace elements (μg/g) in fingernails and reference range were showed. Mn Mean ±SD = 1.00 µg/g ±0.23 µg/g. Reference Range:0.10–1.48 µg/g.	MMSE and Montreal Cognitive Assessment (MoCA).	_	Bivariate analysis only. Concentrations of Mn in fingernail were found to be inversely correlated with MoCA score r = −0.496, *p* < 0.001) and MMSE score (r = −0.159, *p* = 0.250).
**Guarneros et al. (2013)/Mexico ^d^** [39]	Cross-sectional. Subjects from a Mn mining district living <1 km from a Mn processing plant (Tolago/Chiconcoac), in the central Mexican Molango state (*n* = 30), were compared to non-exposed subjects living 50 km from the closest source of exposure (*n* = 30) (same state as the rest of Mexican studies).	Biomarkers of Mn exposure (hair). The exposed subjects had significantly higher concentrations of Mn in hair (MnH) than the control subjects: median scores = 9.73 μg/g versus 1.01 μg/g, *p* < 0.001.	_	Sniffin’ Sticks Test battery (olfactory function as surrogate of early motor function decline).	Exposed and non-exposed groups were matched for gender. Bivariate analysis only. As overall performance for each subject in the Sniffin’ Sticks Test is a 3 subtest battery, the results of the 3 subtests were summed to give a composite threshold–discrimination–identification (TDI) score (maximum of 16 + 16 + 13 = 45). A tendential negative correlation was found between MnH and the performance of subjects within each group on each of the olfactory tests of threshold, discrimination, identification, and TDI scores, but specific correlation values are not reported. Median scores in the overall results of the 3 subtests, were higher in exposed (*p* < 0.001).
**Lucchini et al. (2014)/Italy ^b,d^** [40]	255 elderly healthy subjects (≥60 years, without cognitive impairment) out of a total of 365 originally enrolled, from two regions, one Industrial, next to closed Mn alloy plants (Valcamonica, *n* = 153) exposed to significantly higher environmental levels than the reference region (Garda Lake reference area, *n* = 102).	Air Mn evaluation. Mean airborne M*n* = 26.41 ng/m^3^ (median 18.42) in Valcamonica and 20.96 ng/m^3^ (median 17.62) in the reference area. Biomarkers of Mn exposure (Blood, Urine). Blood Mean Valcamonica = 8.4 Range: 3.6–19.5 µg/L. Blood Mean Garda Lake = 10.2 Range: 3.6–21.6 µg/L. Urine Mean Valcamonica = 0.3 Range: 0.1–6 µg/L. Urine Mean Garda Lake = 0.4. Range: 0.1–9.4 µg/L.	MMSE, Story Recall Test, The Raven’s Colored Progressive Matrices (CPM) test, TMT *, WAIS III Digit Span, WAIS III Digit Symbol.	Luria Nebraska Neuropsychological Battery (LNNB), Finger Tapping Test, Simple Visual Reaction Time *, CATSYS *, Sniffin’ Sticks Test battery.	Multivariate analysis including age, gender, tobacco, alcohol, distance from the source and Pb Blood levels. Results also stratified by geographic area. A negative significant association between the motor coordination test of the LNNB, and airborne Mn (*p* = 0.0237) and the distance from the nearest ferroMn plant point source (*p* = 0.0035) was found. For the odor identification score of the Sniffin’ Sticks Test, an association was observed with soil Mn (*p* = 0.0006). Significant dose–responses resulted also for the Raven’s Colored Progressive Matrices with the distance from exposure point source (*p* = 0.0025) and Mn in soil (*p* = 0.09), and for the TMT, with urinary Mn (*p* = 0.0074).
**Viana et al. (2014)/Brazil ^a,b,c,d^** [41]	Cross-sectional. Healthy population (without cognitive impairment), from two communities of the town of Simões Filho, Bahia: Cotegipe and Santa Luzia villages. These communities are situated at an approximate distance of 1.5 and 2.5 km, respectively, from the ferroMn alloy plant.	Biomarkers of Mn exposure (scalp hair, axillary hair, fingernails and saliva) (µg/g). Cotegipe Mn exposure: Mn scalp hair (MnH) Media*n* = 2.7; range (0.6–44,6). Mn axillary hair (MnAxH) Media*n* = 5.8 µg/g; range (3.8 µg/g–17.2 µg/g). Mn fingernails (MnFN) Media*n* = 4.0; range (0.7–16.1). Mn saliva (MnSal) media*n* = 3.0; range (0.4–43.3) Santa Luzia Mn exposure: MnH Media*n* = 10.5; range (0.9–42,0). MnAxH Media*n* = 21.8; range (4.4–85.6). MnFN Media*n* = 6.5; range (1.1–22.2). MnSal media*n* = 3.7; range (0.6–81.6).	WAIS III, Rey Auditory Verbal Learning Test (RAVLT), Visual Attention Computerized Test, third version (TAVIS-3) *, TMT *, WAIS III Digit Span, Corsi Block-Tapping Task.	Grooved Pegboard *	Multivariate analysis studying as possible covariates: gender, local of residence, time in years of residence in the communities, drinking habits, age, and family income. Significant correlations were observed between MnH levels and WAIS III IQ scores (r = −0.349, *p* = 0.002), Corsi Block-Tapping Task visual working memory (r = −0.251, *p* = 0.024) and with motor function for the dominant hand according to the GPT (r = 0.223, *p* = 0.045). MnAxH was negatively associated with IQ scores (r = −0.495, *p* = 0.043), visual working memory (r = −0.717, *p* = 0.001), Digit Span from the WAIS III verbal working memory (r = −0.303, *p* = 0.009); and with motor function for the dominant (r = 0.530, *p* = 0.024) and nondominant hand (r = 0.618, *p* = 0.005). MnFN were negatively associated with visual working memory (r = −0.717, *p* = 0.009) and with motor function for the dominant hand (r = 0.231, *p* = 0.05). MnSal was not significantly correlated with any of the neuropsychological functions evaluated. Statistically nonsignificant differences in medians, were reported between Cotegipe and Santa Luzia communities.
**Bowler et al. (2015)/US ^a^** [42]	Cross-sectional. Healthy population (without cognitive impairment), belonging to two towns (Marietta, *n* = 100 and East Liverpool, *n* = 86, from Ohio) both highly exposed to environmental Mn from industrial sources. The Marietta exposure group is the same in Bowler et al. (2012, 2015 and 2016) studies.	Air Mn evaluation. Mean in Air ±SD = 0.2 µg/m^3^ ± 0.2 µg/m^3^. Media*n* = 0.2 (Marietta). Mean in Air ±SD = 0.9 µg/m^3^ ± 1.2 µg/m^3^. Media*n* = 0.3 (East Liverpool). Biomarkers of Mn exposure (Blood) not presented.	Stroop Color Word test, Rey Osterrieth complex figure, TMT *, Neuropsychological Assesment Battery (NAB), WAIS III Digit Span, WAIS III Digit Symbol, WAIS III similarities, animal naming, Victoria Sympton Validity Test, Auditory Consonant Trigrams (ACT).	_	Multivariate analysis using town of residence and education (for tests not already adjusted for education) and age when appropriate. Controlling for ‘‘town’’ (as reported by authors) effectively and parsimoniously controls for any differences between them (e.g., age, income, ethnicity, years of residence). No significant differences appeared for any of the neuropsychological test variables using independent sample t-tests. Significant inverse relationships occurred between modeled air-Mn concentrations and test performance for cognitive measures of visuospatial memory (Rey-O Immediate and Delayed) and verbal skills (WAIS Similarities and Animal Naming). Significant relationships (*p* < 0.05) were found between modeled air-Mn exposure and performance on working and visuospatial memory (e.g., Rey-O Immediate β = −0.19, Rey-O Delayed β = −0.16) and verbal skills (e.g., WAIS Similarities β = −0.19), after controlling for education and town of residence.
**Bowler et al. (2016)/US ^c^** [28]	Cross-sectional. Bowler et al. (2015 and 2016) studies are the same studies, but one shows cognitive function and the other motor function respectively.	Same exposure as Bowler et al. (2015).	_	Finger Tapping Test, Hand Dynamometer, Grooved Pegboard *, CATSYS*.	Unadjusted Bayesian path analysis models used. Significant town differences were seen for all means comparisons in tremor test z-scores, and motor function test T-scores with the exception of Grooved Pegboard, nondominant. Air-Mn exposure was significantly correlated for the combined towns, with the tremor test (CATSYS) for intensity, center frequency and HI. Finger Tapping T–scores were also significantly negatively correlated with air-Mn, as were the Grooved Pegboard nondominant hand T–scores.
**Iqbal et al. (2018)/Pakistan ^a^** [43]	Cross-sectional. 183 patients diagnosed with cognitive impairment (MMSE score ≤24); mild (*n* = 72) (MMSE scores range 21–24), moderate (*n* = 86) (MMSE scores range 10–20) and severe (*n* = 25) (MMSE score < 10), were compared to age-matched healthy controls (*n* = 90) (MMSE scores ranged 25–30).	Biomarkers of Mn exposure (Blood) Mean ±SD is reported for each group (μg/L). Mn levels were significantly higher in severe (92.08 ± 6.8 μg/L), moderate (77.8 ± 2.4 μg/L) and mild (64.97 ± 3.76 μg/L) cognitively impaired group as compared to the age-matched healthy control group (52.8 ± 2.8 μg/L), *p* < 0.001, *p* < 0.001 and *p* < 0.05 respectively.	MMSE.	_	Cognitive impairment patients matched by ages with healthy controls. Bivariate analysis only. Results showed that Mn and the rest of elements studied were significantly higher in the cognitively impaired patients and increasing concentration was strongly correlated with the increase in severity of the disease. Person’s correlation test revealed negative correlations between the metal concentration and MMSE scores. The maximum correlation was observed with Al (r = −0.638; *p* < 0.001) followed by Cu (r = −0.610; *p* < 0.001), Pb (r = −0.554; *p* < 0.001), Cd (r = −0.418; *p* < 0.001), Mn (r = −0.417; *p* < 0.001) and Zn (r = −0.329; *p* < 0.001) respectively.
**Cabral Pinto et al. (2018)/Portugal ^e^** [44]	Cross-sectional. 103 permanent residents from the industrial city of Estarrejal (>55 years old). 40.2% of the subjects had a normal performance on neurological tests assessing cognitive status. 18.3% showed a mild cognitive impairment compatible with Mild Cognitive Impairment (MCI) condition (considering the cut-off for MCI established in Portuguese validation studies and Clinical Dementia Rating Scale (CDR) = 0.5) and 36.6% had a cognitive performance suggestive of dementia condition (CDR and MMSE and MoCA scores below the respective thresholds).	Biomarkers of potentially toxic elements exposure (urine) including aluminium, cadmium, zinc and Mn exposure (among others). Mean of Mn concentratio*n* = 46.4 ± 217 μg/g, Mode = 0.83. Max 1694. P5-P95 = 0.39–57. Reference for healthy people (0.11–1.32). Also groundwater levels assessed.	MMSE, MoCA, CDR, Geriatric Depression Scale (GDS).	_	Multivariate linear regression models showed that aluminium (R^2^ = 38%), cadmium (R^2^ = 11%) and zinc (R^2^ = 6%) were good predictors of the scores of the MMSE cognitive test. Mn was not shown as a good predictor (the specific R^2^ result for Mn is not reported). Specific covariates included in the multivariate models to control confounding not reported.
**Kornblith et al. (2018)/US ^e^** [45]	Cross-sectional. Same study population as Bowler et al. (2015 and 2016) studies: residents of Marietta (*n* = 99) and East Liverpool (*n* = 83).	See Bowler et al. (2015) for means and medians details.	UPDRS-ADL *, Animal naming, Stroop color word, TMT *, Rey Osterrieth Complex Figure, ACT.	CATSYS*, UPDRS (tremor and motor)*.	Two-step cluster analyses were used. Four distinct symptom clusters were identified in this sample: The largest identified group (Cluster 1: Non-Impaired) contained 60% of the sample and was characterized by average scores (within one standard deviation of the overall sample mean) on measures of gait disturbance, bradykinesia/rigidity, and tremor, and the absence of EF impairment. The second-largest group (Cluster 3: Executive Dysfunction) contained 20% of the sample and consisted of average scores on measures of tremor, gait disturbance and bradykinesia/rigidity, but all members met criteria for EF impairment. The third-largest group (Cluster 2: Tremor) contained 11% of the sample and was characterized by high tremor and average bradykinesia and rigidity. The smallest group (Cluster 4: No Tremor) contained 7% of the sample and had high levels of gait disturbance and bradykinesia/rigidity with relatively lower levels of tremor.
**Rafiee et al. (2019)/Iran ^e^** [46]	Cross-sectional. 200 healthy volunteered participants (110 men and 90 women), aged 14–70 years, without cognitive impairment from Tehran.	Chronic exposure to metals (Cd, Be, Co, Hg, Sn, V, Al, Ba, Cr, Cu, Fe, Li, Mn, Ni, Pb, and Zn) and metalloids (As, B, Sb) through hair samples. Biomarkers of Mn exposure (Hair). Median MnH 3.05 μg/g; range (1.2–12.4). Mean MnH 3.86 μg/L± 3.37, range (0.8–18.4).	TMT *		Multivariate analysis using the following selected variables after studying their effect as confounders: age, gender, self-reported residential traffic exposure, existence of dental amalgam implants, cigarette smoking, water-pipe smoking and insecticide use. Mn levels in hair were significantly associated with poorer participants’ performance scores in the TMT test (more time in seconds), (*p* < 0.05). 0.201 and 0.204 more seconds per one 1 µg/g of Mn in TMT-A and B score respectively

SD: Standard deviation. P5: 5th percentile. P95: 95th percentile. ^a^ Included in the meta-analysis of the cognitive function. Correlation section. ^b^ Included in the meta-analysis of cognitive function. Standardized mean difference (SMD) section. ^c^ Included in the meta-analysis of the motor function. Correlation section. ^d^ Included in the meta-analysis of motor function. SMD section. ^e^ Not included in the meta-analysis. * For these tests, the higher the score, the worse the function. For the rest of the tests, the lower the score, the worse the function.

**Table 3:** Correlation between cognitive function and exposure to Mn. Overall heterogeneity and as a function of the cognitive test used.

Cognitive Function	N of Studies	N of Determinations	Heterogeneity					
Correlation			Q	df	p (Chi^2^)	I^2^ (%)	Tau^2^	Tau
ACT	1	4	0.67	3.00	0.879	0.00	0.00	0.00
Corsi Block-Tapping Task	1	4	27.60	3.00	0.000	89.13	0.10	0.32
Mini-Mental State Examination	2	2	3.45	1.00	0.06	71.04	0.03	0.17
Montreal Cognitive Assessment	1	1	0.00	0.00	1.00	0.00	0.00	0.00
NAB	1	3	0.49	2.00	0.781	0.00	0.00	0.00
RAVLT	1	4	1.59	3.00	0.661	0.00	0.00	0.00
ROCF	1	3	0.40	2.00	0.817	0.00	0.00	0.00
Stroop Color Word Test	1	3	1.65	2.00	0.437	0.00	0.00	0.00
TAVIS-3	1	8	7.66	7.00	0.364	8.59	0.00	0.03
Trail Making Test	2	10	57.71	9.00	0.000	84.40	0.06	0.24
WAIS III	2	14	29.19	13.00	0.006	55.47	0.01	0.10
All studies and determinations	4	56	233.92	55.00	0.000	76.49	0.03	0.16

ACT: Auditory Consonant Trigrams. NAB: Neuropsychological Assessment Battery. RAVLT: Rey Auditory Verbal Learning Test. ROCF: Rey Osterrieth Complex Figure test. TAVIS-3: Visual Attention Computerized Test, third version. WAIS III: Weschler Intelligence Scale for Adults, third version.

**Table 4:** Standardized mean differences (SMDs). Overall heterogeneity and as a function of the cognitive test used.

Cognitive Function	N of Studies	N of Determinations	Heterogeneity					
SMD			Q	df	p (Chi^2^)	I^2^ (%)	Tau^2^	Tau
Corsi Block-Tapping Test	1	1	0.0	0.00	1.000	0.00	0.00	0.00
CPM	1	2	0.00	1.00	0.958	0.00	0.02	0.00
MMSE	1	2	0.12	1.00	0.729	0.00	0.02	0.00
RAVLT	1	1	0.00	0.00	1.000	0.00	0.00	0.00
StoryRecall Test	1	3	1.07	2.00	0.586	0.00	0.02	0.00
TAVIS-3	1	2	1.18	1.00	0.277	15.29	0.08	0.09
Trail Making Test	2	6	4.46	5.00	0.485	0.00	0.01	0.00
UPDRS	1	1	0.00	0.00	1.000	0.00	0.00	0.00
WAIS III	3	8	9.11	7.00	0.245	23.20	0.01	0.08
All tests	3	26	25.67	25.00	0.425	2.62	0.01	0.02

CPM: Raven’s Colored Progressive Matrices Test. MMSE: Mini-Mental State Examination. RAVLT: Rey Auditory Verbal Learning Test. ROCF: Rey Osterrieth Complex Figure test. TAVIS-3: Visual Attention Computerized Test, third version. UPDRS: Unified Parkinson’s Disease Rating Scale. WAIS III: Weschler Intelligence Scale for Adults, third version.

**Table 5:** Correlation between motor function and exposure to Mn. Overall heterogeneity and as a function of the motor test used.

Motor Function	N of Studies	N of Determinations	Heterogeneity					
Correlation			Q	df	p (Chi^2^)	I^2^ (%)	Tau^2^	Tau
CATSYS	1	6	99.607	5.00	0.000	94.98	0.10	0.32
Dynamometer	1	2	0.01	1.00	0.924	0.00	0.00	0.00
Finger Tapping	1	2	1.00	1.00	0.316	0.34	0.00	0.00
Grooved Pegboard	2	10	9.75	9.00	0.371	7.74	0.00	0.03
Postural Balance	1	16	16.40	15.00	0.356	8.53	0.00	0.05
All studies and determinations	3	36	139.97	35.00	0.000	74.99	0.03	0.18

CATSYS: Coordination Ability Test System.

**Table 6:** Standardized mean differences (SMDs). Overall heterogeneity and as a function of the motor test used.

Motor Function	N of Studies	N of Determinations	Heterogeneity					
SMD			Q	df	p (Chi^2^)	I^2^ (%)	Tau^2^	Tau
CATSYS	2	32	31.05	31.00	0.464	0.15	0.00	0.00
Dynamometer	1	2	0.34	1.00	0.557	0.00	0.00	0.00
EKM	1	4	0.82	3.00	0.846	0.00	0.00	0.00
Finger Tapping	2	4	1.34	3.00	0.719	0.00	0.00	0.00
Grooved Pegboard	2	4	2.2	3.00	0.530	0.00	0.00	0.00
Luria Nebraska	1	6	3.55	5.00	0.616	0.00	0.00	0.00
Postural Balance	1	8	7.11	7.00	0.417	1.59	0.02	0.04
Purdue Pegboard	1	2	13.10	1.00	0.000	92.36	0.41	0.64
Simple Visual Reaction Time	1	2	0.18	1.00	0.668	0.00	0.00	0.00
Sniffin’ sticks	2	5	5.78	4.00	0.216	30.83	0.02	0.15
UPDRS	1	2	0.01	1.00	0.903	0.00	0.00	0.00
All test	7	71	146.69	70.00	0.000	52.28	0.02	0.16

CATSYS: Coordination Ability Test System. EKM: Eurythmokinesimeter. UPDRS: Unified Parkinson’s Disease Rating Scale.

## Supplementary Materials

The following are available online at https://www.mdpi.com/article/10.3390/ijerph18084075/s1, Table S1. NHLBI Quality Assessment Tool for Observational Cohort and Cross-Sectional Studies. Studies included in meta-analysis. Table S2. NHLBI Quality Assessment Tool for Observational Cohort and Cross-Sectional Studies. Studies not included in the meta-analysis. Table S3. Correlation between cognitive function and exposure to Mn. Overall heterogeneity and as a function of the cognitive domain assessed. Table S4. Correlation between cognitive function and exposure to Mn. Overall heterogeneity and as a function of the type of exposure to Mn evaluated. Table S5. Standardized mean differences (SMDs). Cognitive function. Overall heterogeneity and as a function of the cognitive domain assessed. Table S6. Standardized mean differences (SMDs). Cognitive function. Overall heterogeneity and as a function of the type of exposure to Mn evaluated. Table S7. Correlation between motor function and exposure to Mn. Overall heterogeneity and as a function of the motor domain assessed. Table S8. Correlation between motor function and exposure to Mn. Overall heterogeneity and as a function of the type of exposure to Mn evaluated. Table S9. Standardized mean differences (SMDs). Motor function. Overall heterogeneity and as a function of the motor domain assessed. Table S10. Standardized mean differences (SMDs). Motor function. Overall heterogeneity and as a function of the type of exposure to Mn evaluated. Figure S1. Correlation between cognitive function and Mn exposure, as a function of the cognitive test used. Figure S2. Correlation between cognitive function and Mn exposure, as a function of the cognitive domain assessed. Figure S3. Correlation between cognitive function and Mn exposure, as a function of the type of exposure to Mn evaluated. Figure S4. Cognitive function. Standardized mean differences (SMDs) between groups, prioritizing the use of medians versus means (when both medians and means were reported). All tests and all exposures to Mn. Figure S5. Cognitive function. Standardized mean differences (SMDs) between groups, as a function of the cognitive test used. Figure S6. Cognitive function. Standardized mean differences (SMDs) between groups, as a function of the cognitive domain assessed. Figure S7. Cognitive function. Standardized mean differences (SMDs) between groups, as a function of the type of exposure to Mn evaluated. Figure S8. Correlation between motor function and Mn exposure, as a function of the motor test used. Figure S9. Correlation between motor function and Mn exposure, as a function of the motor domain assessed. Figure S10. Correlation between motor function and Mn exposure, as a function of the type of exposure to Mn evaluated. Figure S11. Motor function. Standardized mean differences (SMDs) between groups, prioritizing the use of medians versus means (when both medians and means were reported). All tests and all exposures to Mn. Figure S12. Motor function. Standardized mean differences (SMDs) between groups, as a function of the motor test used. Figure S13. Motor function. Standardized mean differences (SMDs) between groups, as a function of the motor domain assessed. Figure S14. Motor function. Standardized mean differences (SMDs) between groups, as a function of the type of exposure to Mn evaluated.

## Abbreviations

ADL	Activities of daily living
ACT	Auditory Consonant Trigrams
CDR	Clinical Dementia Rating Scale
CATSYS	Coordination Ability Test System
DIADO	Diadocchokinesimetry
EKM	Eurythmokinesimetry
GDS	Geriatric Depression Scale
HRQOL	Health Related Quality of Life
HVOT	Hooper visual organization test
MSQ	Medical symptoms questionnaires
LNNB	Luria Nebraska Neuropsychological Battery
MMSE	Mini-Mental State Examination
Mn-Air	Mn in ambient air
MnH	Mn in hair
MnAxH	Mn in axillary hair
MnFN	Mn in fingernails
MnSal	Mn in saliva
MoCA	Montreal Cognitive Assessment
NAB	Neuropsychological Assessment Battery
CPM	Raven’s Colored Progressive Matrices
RAVLT	Rey Auditory Verbal Learning test
ROCF	Rey Osterrieth Complex Figure
SCL-90R	Symptom Checklist 90 revised
TMT	Trail Making Test
HI	Tremor Harmonic index
DBP	Tremor system of Danish Product Development
UPDRS	Unified Parkinson’s Disease Rating Scale
TAVIS-3	Visual Attention Computerized Test, third version
WAIS III	Wechsler Intelligence Scale for Adults, third version
