# Supplementary material for: Impact of Environmental Airborne Manganese Exposure on Cognitive and Motor Functions in Adults: A Systematic Review and Meta-Analysis
Source: Int J Environ Res Public Health. 2021 Apr 13;18(8):4075. doi: 10.3390/ijerph18084075 (PMC8068914; doi:10.3390/ijerph18084075)
Supplement: Supplementary file 1 [file ijerph-18-04075-s001.zip › ijerph-1157582-supplementary.pdf]

International Journal of
Environmental Research and Public Health

Online supplementary material

Online supplementary Tables and Figures

Online supplementary Table S1. *NHLBI Quality Assessment Tool for Observational Cohort and Cross-Sectional Studies. Studies included in meta-analysis.*

	**Bowler et al. (2012)** [37]	**Bowler et al. (2015)** [42]	**Bowler et al. (2016)** [28]	**Ghazali et al. (2013)** [38]	**Iqbal et al. (2018)** [43]	**Kim et al. (2011)** [36]	**Lucchini et al. (2014)** [40]	**Mergler et al. (1999)** [30]	**Guarneros et al. (2013)** [39]	**Viana et al. (2014)** [41]	**Standridge et al. (2008)** [34]
1. Was the research question or objective in this paper clearly stated?	Yes	Yes	Yes	Yes	Yes	Yes	Yes	Yes	Yes	Yes	Yes
2. Was the study population clearly specified and defined?	Yes	Yes	Yes	No	No	Yes	Yes	Yes	No	Yes	Yes
3. Was the participation rate of eligible persons at least 50%?	Yes	Yes	Yes	CD	CD	Yes	Yes	Yes	CD	No	CD
4. Were all the subjects selected or recruited from the same or similar populations (including the same time period)? Were inclusion and exclusion criteria for being in the study prespecified and applied uniformly to all participants?	Yes	Yes	Yes	CD	CD	Yes	Yes	Yes	CD	Yes	Yes
5. Was a sample size justification, power description, or variance and effect estimates provided?	No	No	No	No	No	No	No	No	No	No	No
6. For the analyses in this paper, were the exposure(s) of interest measured prior to the outcome(s) being measured?	No	No	No	No	No	No	No	No	No	No	No
7. Was the timeframe sufficient so that one could reasonably expect to see an association between exposure and outcome if it existed?	No	No	No	No	No	No	No	No	No	No	No
8. For exposures that can vary in amount or level, did the study examine different levels of the exposure as related to the outcome (e.g., categories of exposure, or exposure measured as continuous variable)?	Yes	Yes	Yes	Yes	Yes	Yes	Yes	Yes	Yes	Yes	Yes
9. Were the exposure measures (independent variables) clearly defined, valid, reliable, and	Yes	Yes	Yes	Yes	CD	Yes	Yes	Yes	Yes	Yes	Yes

implemented consistently across all study participants?											
10. Was the exposure(s) assessed more than once over time?	No	No	No	No	No	No	No	No	No	No	No
11. Were the outcome measures (dependent variables) clearly defined, valid, reliable, and implemented consistently across all study participants?	Yes	Yes	Yes	Yes	CD	Yes	Yes	Yes	Yes	Yes	Yes
12. Were the outcome assessors blinded to the exposure status of participants?	NR	NR	NR	NR	NR	NR	NR	NR	NR	NR	NR
13. Was loss to follow-up after baseline 20% or less?	NA	NA	NA	NA	NA	NA	NA	NA	NA	NA	NA
14. Were key potential confounding variables measured and adjusted statistically for their impact on the relationship between exposure(s) and outcome(s)?	Yes	Yes	No	No	No	Yes	Yes	Yes	No	Yes	Yes
Rating (Good, Fair, or Poor)	Fair-Good	Fair-Good	Fair	Fair	Poor	Good	Good	Good	Fair	Fair-Good	Fair-Good

Available at: http://www.nhlbi.nih.gov/health-pro/guidelines/in-develop/cardiovascular-risk-reduction/tools/cohort. CD, cannot determine; NA, not applicable; NR, not reported.

Online supplementary Table S2. *NHLBI Quality Assessment Tool for Observational Cohort and Cross-Sectional Studies. Studies non-included in meta-analysis.*

	**Beuter et al. (1999)** [31]	**Santos Burgoa et al. (2001)** [32]	**Rodríguez-Agudelo et al. (2006)** [33]	**Solís Vivanco et al. (2009)** [35]	**Cabral Pinto et al. (2018)** [44]	**Kornblith et al. (2018)** [45]	**Rafiee et al. (2019)** [46]
1. Was the research question or objective in this paper clearly stated?	Yes	Yes	Yes	Yes	Yes	Yes	Yes
2. Was the study population clearly specified and defined?	Yes	Yes	Yes	Yes	No	Yes	Yes
3. Was the participation rate of eligible persons at least 50%?	Yes	CD	Yes	Yes	CD	Yes	CD
4. Were all the subjects selected or recruited from the same or similar populations (including the same time period)? Were inclusion and exclusion criteria for being in the study prespecified and applied uniformly to all participants?	Yes	CD	Yes	Yes	CD	Yes	CD
5. Was a sample size justification, power description, or variance and effect estimates provided?	No	No	Yes	Yes	No	No	No
6. For the analyses in this paper, were the exposure(s) of interest measured prior to the outcome(s) being measured?	No	No	No	No	No	No	No
7. Was the timeframe sufficient so that one could reasonably expect to see an association between exposure and outcome if it existed?	No	No	No	No	No	No	No
8. For exposures that can vary in amount or level, did the study examine different levels of the exposure as related to the outcome (e.g., categories of exposure, or exposure measured as continuous variable)?	Yes	Yes	Yes	Yes	Yes	Yes	Yes
9. Were the exposure measures (independent variables) clearly defined, valid, reliable, and implemented consistently across all study participants?	Yes	Yes	Yes	Yes	Yes	Yes	Yes
10. Was the exposure(s) assessed more than once over time?	No	No	No	No	No	No	No
11. Were the outcome measures (dependent variables) clearly defined, valid, reliable, and implemented consistently across all study participants?	Yes	Yes	Yes	Yes	Yes	Yes	Yes
12. Were the outcome assessors blinded to the exposure status of participants?	NR	NR	NR	NR	NR	NR	NR
13. Was loss to follow-up after baseline 20% or less?	NA	NA	NA	NA	NA	NA	NA
14. Were key potential confounding variables measured and adjusted statistically for their impact on the relationship between exposure(s) and outcome(s)?	Yes	Yes	Yes	Yes	CD	NA	Yes
Rating (Good, Fair, or Poor)	Good	Fair	Good	Good	Fair	Fair	Fair

Available at: http://www.nhlbi.nih.gov/health-pro/guidelines/in-develop/cardiovascular-risk-reduction/tools/cohort. CD, cannot determine; NA, not applicable; NR, not reported.

Online supplementary Table S3. *Correlation between cognitive function and exposure to Mn. Overall heterogeneity and as a function of the cognitive domain assessed.*

Cognitive function Correlation	N of studies	N of determinations	Heterogeneity					
			Q	df	p (Chi²)	I² (%)	Tau²	Tau
Attention	2	18	94.66	7.00	0.000	82.04	0.05	0.22
Attention and working memory	2	7	14.13	6.00	0.028	57.54	0.01	0.11
Executive functions	1	3	1.65	2.00	0.437	0.00	0.00	0.00
Fluency	1	1	0.00	0.00	1.000	0.00	0.00	0.00
General cognitive functioning	3	6	21.99	5.00	0.001	77.26	0.02	0.15
Intelligence	1	4	8.58	3.00	0.035	65.06	0.02	0.15
Memory	2	15	64.63	14.00	0.000	78.34	0.03	0.17
Proccesing speed	1	1	0.00	0.00	1.000	0.00	0.00	0.00
Verbal comprehension	1	1	0.00	0.00	0.000	0.00	0.00	0.00
All domains	3	56	233.92	55.00	0.000	76.49	0.03	0.16

Online supplementary Table S4. *Correlation between cognitive function and exposure to Mn. Overall heterogeneity and as a function of the type of exposure to Mn evaluated.*

Cognitive function Correlation	N of studies	N of determinations	Heterogeneity					
			Q	df	p (Chi²)	I² (%)	Tau²	Tau
Air Mn	1	21	16.37	20.00	0.693	0.00	0.00	0.00
Axillary Hair Mn	1	8	106.45	7.00	0.000	93.42	0.18	0.42
Blood Mn	1	1	0.00	0.00	1.000	0.00	0.00	0.00
Hand Fingernails Mn	3	10	21.92	10.00	0.009	58.94	0.02	0.14
Saliva Mn	1	8	2.36	7.00	0.938	0.00	0.00	0.00
Scalp Hair Mn	1	8	21.35	7.00	0.003	67.21	0.03	0.16
All types of exposure to Mn evaluated	4	56	233.92	55.00	0.000	76.49	0.03	0.16

Online supplementary Table S5. *Standardized mean differences (SMDs). Cognitive function. Overall heterogeneity and as a function of the cognitive domain assessed.*

Cognitive function. SMD	N of studies	N of determinations	Heterogeneity					
			Q	df	p (Chi²)	I² (%)	Tau²	Tau
Attention	2	8	6.03	7.00	0.536	0.00	0.00	0.00
Attention and working memory	2	4	0.33	3.00	0.955	0.00	0.00	0.00
General cognitive functioning	2	5	2.08	4.00	0.722	0.00	0.00	0.00
Intelligence	1	1	0.00	0.00	1.000	0.00	0.00	0.00
Memory	2	5	8.11	4.00	0.088	50.70	0.02	0.15
Processing speed	1	2	0.58	1.00	0.448	0.00	0.00	0.00
Verbal comprehension	1	1	0.00	0.00	1.000	0.00	0.00	0.00
All domains	3	26	25.67	25.00	0.425	2.62	0.00	0.02

Online supplementary Table S6. *Standardized mean differences (SMDs). Cognitive function. Overall heterogeneity and as a function of the type of exposure to Mn evaluated.*

Cognitivefunction SMD	N of studies	N of determinations	Heterogeneity					
			Q	df	p (Chi²)	I² (%)	Tau²	Tau
Air Mn &blood Mn	1	2	0.02	1.00	0.874	0.00	0.00	0.00
Air Mn, blood & urine Mn	1	16	7.36	15.00	0.947	0.00	0.00	0.00
Scalp Hair, axillary hair, fingernails & saliva Mn	1	8	23.84	7.00	0.021	57.46	0.07	0.26
All types of exposure to Mn evaluated	3	26	25.67	25.00	0.425	2.62	0.00	0.02

Online supplementary Table S7. *Correlation between motor function and exposure to Mn. Overall heterogeneity and as a function of the motor domain assessed.*

Motor function Correlation	N of studies	N of determinations	Heterogeneity					
			Q	df	p (Chi²)	I² (%)	Tau²	Tau
Eye-hand coordination and motor speed	2	10	9.75	9.00	0.371	7.74	0.00	0.03
Hand strength	1	2	0.01	1.00	0.924	0.00	0.00	0.00
Postural balance	1	16	16.40	15.00	0.356	8.53	0.00	0.05
Self-directed manual speed	1	2	1.00	1.00	0.000	0.34	0.00	0.00
Tremor	1	6	99.61	5.00	0.000	94.98	0.10	0.32
All domains	3	36	139.97	35.00	0.000	74.99	0.03	0.18

Online supplementary Table S8. *Correlation between motor function and exposure to Mn. Overall heterogeneity and as a function of the type of exposure to Mn evaluated.*

Motor function Correlation	N of studies	N of determinations	Heterogeneity					
			Q	df	p (Chi²)	I² (%)	Tau²	Tau
Air Mn	1	12	108.83	11.00	0.000	89.89	0.05	0.22
Axillary Hair	1	2	0.13	1.00	0.719	0.00	0.00	0.00
Blood	1	8	2.15	7.00	0.951	0.00	0.00	0.00
Hand Fingernails	1	2	0.64	1.00	0.425	0.00	0.00	0.00
Saliva	1	2	0.06	1.00	0.809	0.00	0.00	0.00
Scalp Hair	2	10	5.37	79.00	0.801	0.00	0.00	0.00
All types of exposure to Mn evaluated	3	36	139.97	35.00	0.000	74.99	0.03	0.18

Online supplementary Table S9. *Standardized mean differences (SMDs). Motor function. Overall heterogeneity and as a function of the motor domain assessed.*

Motor function SMD	N of studies	N of determinations	Heterogeneity					
			Q	df	p (Chi²)	I² (%)	Tau 2	Tau
Bradykinesia	1	1	0.00	0.00	1.000	0.00	0.00	0.00
Eye-hand coordination and motor speed	2	4	0.82	3.00	0.846	0.00	0.00	0.00
Eye-hand coordination	1	4	2.21	3.00	0.530	0.00	0.00	0.00
Finger and hand dexterity	1	2	13.09	1.00	0.000	92.36	0.41	0.64
Hand strength	1	2	0.34	1.00	0.557	0.00	0.00	0.00
Motor coordination	1	6	3.55	5.00	0.616	0.00	0.00	0.00
Motor evaluation	1	1	0.00	0.00	1.00	0.00	0.00	0.00
Olfactory performance	2	5	5.78	4.00	0.216	30.83	0.02	0.15
Postural balance	1	8	7.11	7.00	0.417	1.59	0.00	0.04
Postural sway	2	24	23.85	23.00	0.412	3.56	0.00	0.03
Reaction time	1	2	0.18	1.00	0.668	0.00	0.00	0.00
Self-directed manual speed	2	4	1.34	3.00	0.719	0.00	0.00	0.00
Tremor	1	8	3.43	7.00	0.842	0.00	0.00	0.00
All domains	7	71	146.69	70.00	0.000	52.28	0.02	0.16

Online supplementary Table S10. *Standardized mean differences (SMDs). Motor function. Overall heterogeneity and as a function of the type of exposure to Mn evaluated.*

Motor function SMD	N of studies	N of determinations	Heterogeneity					
			Q	df	p (Chi²)	I² (%)	Tau²	Tau
Air Mn &blood Mn	2	20	21.86	19.00	0.291	13.10	0.00	0.06
Air Mn, blood &urine Mn	1	31	35.47	30.00	0.226	15.42	0.00	0.06
Blood Mn	1	6	22.74	5.00	0.000	78.01	0.11	0.33
Blood &HairMn	1	8	7.11	7.00	0.417	1.59	0.00	0.04
Hair Mn	1	4	0.44	3.00	0.932	0.00	0.00	0.00
Scalp hair, Axillary hair, Fingernails & Saliva Mn	1	2	1.06	1.00	0.303	5.92	0.00	0.05
All types of exposure to Mn evaluated	7	71	146.69	70.00	0.000	52.28	0.02	0.16

Model	Group by Subgroup within study	Study name	Subgroup within study	Correlation	Lower limit	Upper limit	Z-Value	p-Value
	Auditory Consonant Trigrams	Bowler et al., 2015 Auditory Consonant Trigrams 18' delay z-score. Air Mn	Auditory Consonant Trigrams	-0.130	-0.269	0.014	-1.769	0.077
	Auditory Consonant Trigrams	Bowler et al., 2015 Auditory Consonant Trigrams 3' delay z-score. Air Mn	Auditory Consonant Trigrams	-0.070	-0.212	0.075	-0.948	0.343
	Auditory Consonant Trigrams	Bowler et al., 2015 Auditory Consonant Trigrams 9' delay z-score. Air Mn	Auditory Consonant Trigrams	-0.130	-0.269	0.014	-1.769	0.077
	Auditory Consonant Trigrams	Bowler et al., 2015 Auditory Consonant Trigrams mean of 3', 90', 18' z. Air Mn	Auditory Consonant Trigrams	-0.150	-0.288	-0.006	-2.045	0.041
Fixed	Auditory Consonant Trigrams			-0.120	-0.191	-0.048	-3.265	0.001
Random	Auditory Consonant Trigrams			-0.120	-0.191	-0.048	-3.265	0.001
	Corsi Block-Tapping Task	Viana et al., 2014 Corsi Block-Tapping Task. Axilary Hair Mn	Corsi Block-Tapping Task	-0.717	-0.808	-0.592	-8.012	0.000
	Corsi Block-Tapping Task	Viana et al., 2014 Corsi Block-Tapping Task. Hand Fingernails Mn	Corsi Block-Tapping Task	-0.303	-0.488	-0.092	-2.780	0.005
	Corsi Block-Tapping Task	Viana et al., 2014 Corsi Block-Tapping Task. Saliva Mn	Corsi Block-Tapping Task	-0.135	-0.342	0.084	-1.207	0.227
	Corsi Block-Tapping Task	Viana et al., 2014 Corsi Block-Tapping Task. Scalp Hair Mn	Corsi Block-Tapping Task	-0.251	-0.444	-0.036	-2.280	0.023
Fixed	Corsi Block-Tapping Task			-0.381	-0.471	-0.283	-7.140	0.000
Random	Corsi Block-Tapping Task			-0.381	-0.627	-0.067	-2.354	0.019
	Mini-Mental State Examination	Ghazali et al., 2013 Mini-Mental State Examination. Hand Fingernails Mn	Mini-Mental State Examination	-0.159	-0.409	0.114	-1.145	0.252
	Mini-Mental State Examination	Iqbal et al., 2018 Mini-Mental State Examination. Blood Mn	Mini-Mental State Examination	-0.417	-0.510	-0.314	-7.297	0.000
Fixed	Mini-Mental State Examination			-0.379	-0.469	-0.282	-7.148	0.000
Random	Mini-Mental State Examination			-0.319	-0.539	-0.058	-2.375	0.018
	Montreal Cognitive Assesment	Ghazali et al., 2013 Montreal Cognitive Assessment. Hand Fingernails Mn	Montreal Cognitive Assesment	-0.496	-0.674	-0.263	-3.885	0.000
Fixed	Montreal Cognitive Assesment			-0.496	-0.674	-0.263	-3.885	0.000
Random	Montreal Cognitive Assesment			-0.496	-0.674	-0.263	-3.885	0.000
	NAB	Bowler et al., 2015 NAB, Daily Living Memory Delayed Recall T-score. Air Mn	NAB	-0.170	-0.306	-0.027	-2.322	0.020
	NAB	Bowler et al., 2015 NAB, Daily Living Memory Immediate Recall T-score. Air Mn	NAB	-0.150	-0.288	-0.006	-2.045	0.041
	NAB	Bowler et al., 2015 Neuropsychological Assesment Battery (NAB), Memory index. Air Mn	NAB	-0.100	-0.240	0.045	-1.357	0.175
Fixed	NAB			-0.140	-0.221	-0.057	-3.305	0.001
Random	NAB			-0.140	-0.221	-0.057	-3.305	0.001
	RAVLT	Viana et al., 2014 RAVLT. Axilary Hair Mn	RAVLT	0.068	-0.151	0.281	0.605	0.545
	RAVLT	Viana et al., 2014 RAVLT. Hand Fingernails Mn	RAVLT	-0.029	-0.244	0.189	-0.258	0.797
	RAVLT	Viana et al., 2014 RAVLT. Saliva Mn	RAVLT	0.012	-0.206	0.228	0.107	0.915
	RAVLT	Viana et al., 2014 Rey Auditory Verbal Learning Test (RAVLT). Scalp Hair Mn	RAVLT	0.159	-0.060	0.363	1.425	0.154
Fixed	RAVLT			0.053	-0.057	0.162	0.940	0.347
Random	RAVLT			0.053	-0.057	0.162	0.940	0.347
	ROCF	Bowler et al., 2015 Rey Osterrieth Complex Figure test (ROCF), copy raw score. Air Mn	ROCF	-0.150	-0.288	-0.006	-2.045	0.041
	ROCF	Bowler et al., 2015 ROCF, Delayed T. Air Mn	ROCF	-0.200	-0.334	-0.058	-2.743	0.006
	ROCF	Bowler et al., 2015 ROCF, Immedate T. Air Mn	ROCF	-0.210	-0.344	-0.068	-2.884	0.004
Fixed	ROCF			-0.187	-0.266	-0.105	-4.429	0.000
Random	ROCF			-0.187	-0.266	-0.105	-4.429	0.000
	Stroop Color Word test	Bowler et al., 2015 Stroop Color Word test T-score. Air Mn	Stroop Color Word test	-0.160	-0.297	-0.017	-2.183	0.029
	Stroop Color Word test	Bowler et al., 2015 Stroop Color Word test, Color score. Air Mn	Stroop Color Word test	-0.030	-0.173	0.114	-0.406	0.685
	Stroop Color Word test	Bowler et al., 2015 Stroop Color Word test, Word score. Air Mn	Stroop Color Word test	-0.120	-0.259	0.024	-1.631	0.103
Fixed	Stroop Color Word test			-0.104	-0.185	-0.020	-2.437	0.015
Random	Stroop Color Word test			-0.104	-0.185	-0.020	-2.437	0.015
	TAVIS-3	Viana et al., 2014 TAVIS-3, Selective attention. Axilary Hair Mn	TAVIS-3	0.280	0.067	0.469	2.557	0.011
	TAVIS-3	Viana et al., 2014 TAVIS-3, Selective attention. Hand Fingernails Mn	TAVIS-3	0.008	-0.209	0.225	0.071	0.943
	TAVIS-3	Viana et al., 2014 TAVIS-3, Selective attention. Saliva Mn	TAVIS-3	-0.069	-0.282	0.150	-0.614	0.539
	TAVIS-3	Viana et al., 2014 TAVIS-3, Sustained attention. Axilary Hair Mn	TAVIS-3	-0.047	-0.261	0.172	-0.418	0.676
	TAVIS-3	Viana et al., 2014 TAVIS-3, Sustained attention. Hand Fingernails Mn	TAVIS-3	0.114	-0.106	0.323	1.018	0.309
	TAVIS-3	Viana et al., 2014 TAVIS-3, Sustained attention. Saliva Mn	TAVIS-3	0.026	-0.192	0.242	0.231	0.817
	TAVIS-3	Viana et al., 2014 TAVIS-3, Sustained attention. Scalp Hair Mn	TAVIS-3	0.122	-0.098	0.330	1.090	0.276
	TAVIS-3	Viana et al., 2014 Visual Attention Computerized Test, third version (TAVIS-3), Selective attention. Scalp Hair Mn	TAVIS-3	0.147	-0.072	0.353	1.316	0.188
Fixed	TAVIS-3			0.074	-0.004	0.151	1.856	0.063
Random	TAVIS-3			0.074	-0.008	0.154	1.775	0.076
	Trail Making Test	Bowler et al., 2015 Trail Making Test A, T-score. Air Mn	Trail Making Test	0.010	-0.134	0.154	0.135	0.892
	Trail Making Test	Bowler et al., 2015 Trail Making Test B, T-score. Air Mn	Trail Making Test	-0.110	-0.250	0.034	-1.494	0.135
	Trail Making Test	Viana et al., 2014 Trail Making Test A (seconds). Axilary Hair Mn	Trail Making Test	-0.697	-0.794	-0.566	-7.657	0.000
	Trail Making Test	Viana et al., 2014 Trail Making Test A (seconds). Hand Fingernails Mn	Trail Making Test	-0.205	-0.404	0.013	-1.848	0.065
	Trail Making Test	Viana et al., 2014 Trail Making Test A (seconds). Saliva Mn	Trail Making Test	-0.125	-0.333	0.095	-1.117	0.264
	Trail Making Test	Viana et al., 2014 Trail Making Test A (seconds). Scalp Hair Mn	Trail Making Test	-0.130	-0.337	0.090	-1.162	0.245
	Trail Making Test	Viana et al., 2014 Trail Making Test B (seconds). Axilary Hair Mn	Trail Making Test	-0.479	-0.630	-0.292	-4.637	0.000
	Trail Making Test	Viana et al., 2014 Trail Making Test B (seconds). Hand Fingernails Mn	Trail Making Test	-0.328	-0.509	-0.119	-3.027	0.002
	Trail Making Test	Viana et al., 2014 Trail Making Test B (seconds). Saliva Mn	Trail Making Test	-0.057	-0.271	0.162	-0.507	0.612
	Trail Making Test	Viana et al., 2014 Trail Making Test B (seconds). Scalp Hair Mn	Trail Making Test	-0.065	-0.278	0.154	-0.579	0.563
Fixed	Trail Making Test			-0.199	-0.257	-0.138	-6.359	0.000
Random	Trail Making Test			-0.232	-0.377	-0.077	-2.905	0.004
	WAIS III	Bowler et al., 2015 WAIS III, Animal Naming Scaled Score. Air Mn	WAIS III	-0.190	-0.325	-0.047	-2.602	0.009
	WAIS III	Bowler et al., 2015 WAIS III, Digit backward z-score. Air Mn	WAIS III	-0.160	-0.297	-0.017	-2.183	0.029
	WAIS III	Bowler et al., 2015 WAIS III, Digit forward z-score. Air Mn	WAIS III	-0.110	-0.250	0.034	-1.494	0.135
	WAIS III	Bowler et al., 2015 WAIS III, Digit Symbol Scaled Score. Air Mn	WAIS III	-0.120	-0.259	0.024	-1.631	0.103
	WAIS III	Bowler et al., 2015 WAIS III, Similarities Scaled Score. Air Mn	WAIS III	-0.300	-0.426	-0.163	-4.187	0.000
	WAIS III	Bowler et al., 2015 Weschler Intelligence Scale for Adults, third version (WAIS III), Digit Span Scaled Score. Air Mn	WAIS III	-0.190	-0.325	-0.047	-2.602	0.009
	WAIS III	Viana et al., 2014 WAIS III, Digit Span. Axilary Hair Mn	WAIS III	-0.523	-0.665	-0.345	-5.159	0.000
	WAIS III	Viana et al., 2014 WAIS III, Digit Span. Hand Fingernails Mn	WAIS III	-0.112	-0.321	0.108	-1.000	0.317
	WAIS III	Viana et al., 2014 WAIS III, Digit Span. Saliva Mn	WAIS III	-0.137	-0.344	0.082	-1.225	0.220
	WAIS III	Viana et al., 2014 WAIS III, Digit Span. Scalp Hair Mn	WAIS III	-0.155	-0.360	0.064	-1.389	0.165
	WAIS III	Viana et al., 2014 WAIS III. Axilary Hair Mn	WAIS III	-0.495	-0.643	-0.311	-4.823	0.000
	WAIS III	Viana et al., 2014 WAIS III. Hand Fingernails Mn	WAIS III	-0.193	-0.394	0.025	-1.737	0.082
	WAIS III	Viana et al., 2014 WAIS III. Saliva Mn	WAIS III	-0.113	-0.322	0.107	-1.009	0.313
	WAIS III	Viana et al., 2014 WAIS III. Scalp Hair Mn	WAIS III	-0.349	-0.526	-0.143	-3.238	0.001
Fixed	WAIS III			-0.212	-0.257	-0.167	-8.965	0.000
Random	WAIS III			-0.222	-0.290	-0.152	-6.119	0.000
Fixed	Overall			-0.165	-0.188	-0.141	-13.636	0.000
Random	Overall			-0.122	-0.152	-0.093	-8.046	0.000

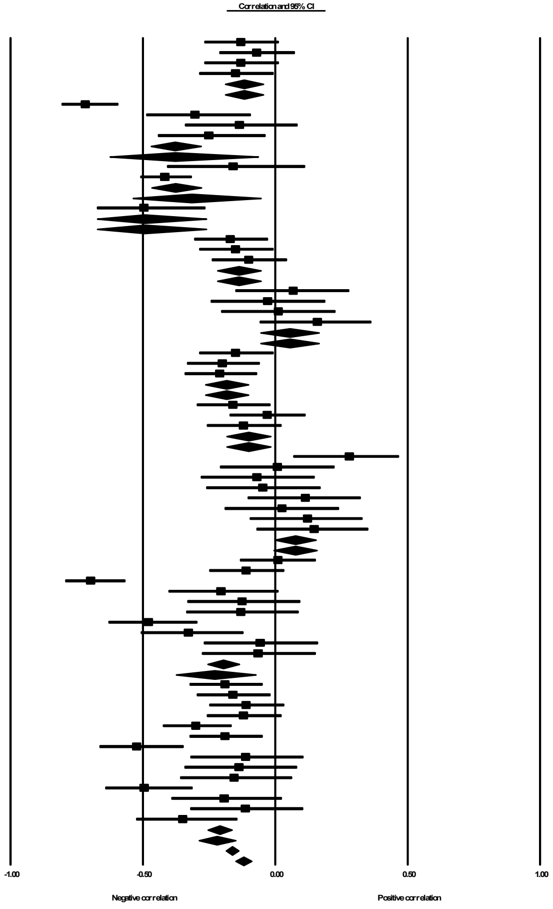

Online supplementary Figure S1. *Correlation between cognitive function and Mn exposure, as a function of the cognitive test used. Note: a negative correlation indicates that the higher the Mn levels, the worse the cognitive function. A random effects model is used to combine studies within each subgroup. A fixed effect model is used to combine subgroups and yield the overall effect. The study-to-study variance (tau-squared) is NOT assumed to be the same for all subgroups - this value is computed within subgroups and NOT pooled across subgroups.*

Model	Group by Subgroup within study	Study name	Subgroup within study	Statistics for each study: Correlation	Lower limit	Upper limit	Z-Value	p-Value
	Attention	Bowler et al., 2015 Trail Making Test A, T-score. Air Mn	Attention	0.010	-0.134	0.154	0.135	0.892
	Attention	Bowler et al., 2015 Trail Making Test B, T-score. Air Mn	Attention	-0.110	-0.250	0.034	-1.494	0.135
	Attention	Viana et al., 2014 Trail Making Test A (seconds). Scalp Hair Mn	Attention	-0.130	-0.337	0.090	-1.162	0.245
	Attention	Viana et al., 2014 Trail Making Test B (seconds). Scalp Hair Mn	Attention	-0.065	-0.278	0.154	-0.579	0.563
	Attention	Viana et al., 2014 Visual Attention Computerized Test, third version (TAVIS-3), Selective attention. Scalp Hair Mn	Attention	0.147	-0.072	0.353	1.316	0.188
	Attention	Viana et al., 2014 TAVIS-3, Sustained attention. Scalp Hair Mn	Attention	0.122	-0.098	0.330	1.090	0.276
	Attention	Viana et al., 2014 Trail Making Test A (seconds). Axilary Hair Mn	Attention	-0.697	-0.794	-0.566	-7.657	0.000
	Attention	Viana et al., 2014 Trail Making Test B (seconds). Axilary Hair Mn	Attention	-0.479	-0.630	-0.292	-4.637	0.000
	Attention	Viana et al., 2014 TAVIS-3, Selective attention. Axilary Hair Mn	Attention	0.280	0.067	0.469	2.557	0.011
	Attention	Viana et al., 2014 TAVIS-3, Sustained attention. Axilary Hair Mn	Attention	-0.047	-0.261	0.172	-0.418	0.676
	Attention	Viana et al., 2014 Trail Making Test A (seconds). Hand Fingernails Mn	Attention	-0.205	-0.404	0.013	-1.848	0.065
	Attention	Viana et al., 2014 Trail Making Test B (seconds). Hand Fingernails Mn	Attention	-0.328	-0.509	-0.119	-3.027	0.002
	Attention	Viana et al., 2014 TAVIS-3, Selective attention. Hand Fingernails Mn	Attention	0.008	-0.209	0.225	0.071	0.943
	Attention	Viana et al., 2014 TAVIS-3, Sustained attention. Hand Fingernails Mn	Attention	0.114	-0.106	0.323	1.018	0.309
	Attention	Viana et al., 2014 Trail Making Test A (seconds). Saliva Mn	Attention	-0.125	-0.333	0.095	-1.117	0.264
	Attention	Viana et al., 2014 Trail Making Test B (seconds). Saliva Mn	Attention	-0.057	-0.271	0.162	-0.507	0.612
	Attention	Viana et al., 2014 TAVIS-3, Selective attention. Saliva Mn	Attention	-0.069	-0.282	0.150	-0.614	0.539
	Attention	Viana et al., 2014 TAVIS-3, Sustained attention. Saliva Mn	Attention	0.026	-0.192	0.242	0.231	0.817
Fixed	Attention			-0.094	-0.142	-0.046	-3.820	0.000
Random	Attention			-0.100	-0.213	0.015	-1.702	0.089
	Attention and Working Memory	Bowler et al., 2015 Weschler Intelligence Scale for Adults, third version (WAIS III), Digit Span Scaled Score. Air Mn	Attention and Working Memory	-0.190	-0.325	-0.047	-2.602	0.009
	Attention and Working Memory	Bowler et al., 2015 WAIS III, Digit forward z-score. Air Mn	Attention and Working Memory	-0.110	-0.250	0.034	-1.494	0.135
	Attention and Working Memory	Bowler et al., 2015 WAIS III, Digit backward z-score. Air Mn	Attention and Working Memory	-0.160	-0.297	-0.017	-2.183	0.029
	Attention and Working Memory	Viana et al., 2014 WAIS III, Digit Span. Scalp Hair Mn	Attention and Working Memory	-0.155	-0.360	0.064	-1.389	0.165
	Attention and Working Memory	Viana et al., 2014 WAIS III, Digit Span. Axilary Hair Mn	Attention and Working Memory	-0.523	-0.665	-0.345	-5.159	0.000
	Attention and Working Memory	Viana et al., 2014 WAIS III, Digit Span. Hand Fingernails Mn	Attention and Working Memory	-0.112	-0.321	0.108	-1.000	0.317
	Attention and Working Memory	Viana et al., 2014 WAIS III, Digit Span. Saliva Mn	Attention and Working Memory	-0.137	-0.344	0.082	-1.225	0.220
Fixed	Attention and Working Memory			-0.186	-0.250	-0.121	-5.539	0.000
Random	Attention and Working Memory			-0.197	-0.295	-0.094	-3.705	0.000
	Executive Functions	Bowler et al., 2015 Stroop Color Word test T-score. Air Mn	Executive Functions	-0.160	-0.297	-0.017	-2.183	0.029
	Executive Functions	Bowler et al., 2015 Stroop Color Word test, Word score. Air Mn	Executive Functions	-0.120	-0.259	0.024	-1.631	0.103
	Executive Functions	Bowler et al., 2015 Stroop Color Word test, Color score. Air Mn	Executive Functions	-0.030	-0.173	0.114	-0.406	0.685
Fixed	Executive Functions			-0.104	-0.185	-0.020	-2.437	0.015
Random	Executive Functions			-0.104	-0.185	-0.020	-2.437	0.015
	Fluency	Bowler et al., 2015 WAIS III, Animal Naming Scaled Score. Air Mn	Fluency	-0.190	-0.325	-0.047	-2.602	0.009
Fixed	Fluency			-0.190	-0.325	-0.047	-2.602	0.009
Random	Fluency			-0.190	-0.325	-0.047	-2.602	0.009
	General Cognitive Functioning	Bowler et al., 2015 Neuropsychological Assesment Battery (NAB), Memory index. Air Mn	General Cognitive Functioning	-0.100	-0.240	0.045	-1.357	0.175
	General Cognitive Functioning	Bowler et al., 2015 NAB, Daily Living Memory Immediate Recall T-score. Air Mn	General Cognitive Functioning	-0.150	-0.288	-0.006	-2.045	0.041
	General Cognitive Functioning	Bowler et al., 2015 NAB, Daily Living Memory Delayed Recall T-score. Air Mn	General Cognitive Functioning	-0.170	-0.306	-0.027	-2.322	0.020
	General Cognitive Functioning	Ghazali et al., 2013 Mini-Mental State Examination (MMSE). Hand Fingernails Mn	General Cognitive Functioning	-0.159	-0.409	0.114	-1.145	0.252
	General Cognitive Functioning	Ghazali et al., 2013 Montreal Cognitive Assessment (MoCA). Hand Fingernails Mn	General Cognitive Functioning	-0.496	-0.674	-0.263	-3.885	0.000
	General Cognitive Functioning	Iqbal et al., 2018 MMSE. Blood Mn	General Cognitive Functioning	-0.417	-0.510	-0.314	-7.297	0.000
Fixed	General Cognitive Functioning			-0.248	-0.308	-0.186	-7.686	0.000
Random	General Cognitive Functioning			-0.248	-0.377	-0.110	-3.467	0.001
	Intelligence	Viana et al., 2014 WAIS III. Scalp Hair Mn	Intelligence	-0.349	-0.526	-0.143	-3.238	0.001
	Intelligence	Viana et al., 2014 WAIS III. Axilary Hair Mn	Intelligence	-0.495	-0.643	-0.311	-4.823	0.000
	Intelligence	Viana et al., 2014 WAIS III. Hand Fingernails Mn	Intelligence	-0.193	-0.394	0.025	-1.737	0.082
	Intelligence	Viana et al., 2014 WAIS III. Saliva Mn	Intelligence	-0.113	-0.322	0.107	-1.009	0.313
Fixed	Intelligence			-0.295	-0.392	-0.191	-5.404	0.000
Random	Intelligence			-0.295	-0.455	-0.117	-3.194	0.001
	Memory	Bowler et al., 2015 Rey Osterrieth Complex Figure test (ROCF), copy raw score. Air Mn	Memory	-0.150	-0.288	-0.006	-2.045	0.041
	Memory	Bowler et al., 2015 ROCF, Immediate T. Air Mn	Memory	-0.210	-0.344	-0.068	-2.884	0.004
	Memory	Bowler et al., 2015 ROCF, Delayed T. Air Mn	Memory	-0.200	-0.334	-0.058	-2.743	0.006
	Memory	Bowler et al., 2015 Auditory Consonant Trigrams 3' delay z-score. Air Mn	Memory	-0.070	-0.212	0.075	-0.948	0.343
	Memory	Bowler et al., 2015 Auditory Consonant Trigrams 9' delay z-score. Air Mn	Memory	-0.130	-0.269	0.014	-1.769	0.077
	Memory	Bowler et al., 2015 Auditory Consonant Trigrams 18' delay z-score. Air Mn	Memory	-0.130	-0.269	0.014	-1.769	0.077
	Memory	Bowler et al., 2015 Auditory Consonant Trigrams mean of 3' , 90' , 18' z. Air Mn	Memory	-0.150	-0.288	-0.006	-2.045	0.041
	Memory	Viana et al., 2014 Corsi Block-Tapping Task. Scalp Hair Mn	Memory	-0.251	-0.444	-0.036	-2.280	0.023
	Memory	Viana et al., 2014 Rey Auditory Verbal Learning Test (RAVLT). Scalp Hair Mn	Memory	0.159	-0.060	0.363	1.425	0.154
	Memory	Viana et al., 2014 Corsi Block-Tapping Task. Axilary Hair Mn	Memory	-0.717	-0.808	-0.592	-8.012	0.000
	Memory	Viana et al., 2014 RAVLT. Axilary Hair Mn	Memory	0.068	-0.151	0.281	0.605	0.545
	Memory	Viana et al., 2014 Corsi Block-Tapping Task. Hand Fingernails Mn	Memory	-0.303	-0.488	-0.092	-2.780	0.005
	Memory	Viana et al., 2014 RAVLT. Hand Fingernails Mn	Memory	-0.029	-0.244	0.189	-0.258	0.797
	Memory	Viana et al., 2014 Corsi Block-Tapping Task. Saliva Mn	Memory	-0.135	-0.342	0.084	-1.207	0.227
	Memory	Viana et al., 2014 RAVLT. Saliva Mn	Memory	0.012	-0.206	0.228	0.107	0.915
Fixed	Memory			-0.157	-0.200	-0.113	-6.912	0.000
Random	Memory			-0.160	-0.254	-0.064	-3.230	0.001
	Proccesing Speed	Bowler et al., 2015 WAIS III, Digit Symbol Scaled Score. Air Mn	Proccesing Speed	-0.120	-0.259	0.024	-1.631	0.103
Fixed	Proccesing Speed			-0.120	-0.259	0.024	-1.631	0.103
Random	Proccesing Speed			-0.120	-0.259	0.024	-1.631	0.103
	Verbal Comprehension	Bowler et al., 2015 WAIS III, Similarities Scaled Score. Air Mn	Verbal Comprehension	-0.300	-0.426	-0.163	-4.187	0.000
Fixed	Verbal Comprehension			-0.300	-0.426	-0.163	-4.187	0.000
Random	Verbal Comprehension			-0.300	-0.426	-0.163	-4.187	0.000
Fixed	Overall			-0.165	-0.188	-0.141	-13.636	0.000
Random	Overall			-0.169	-0.207	-0.130	-8.408	0.000

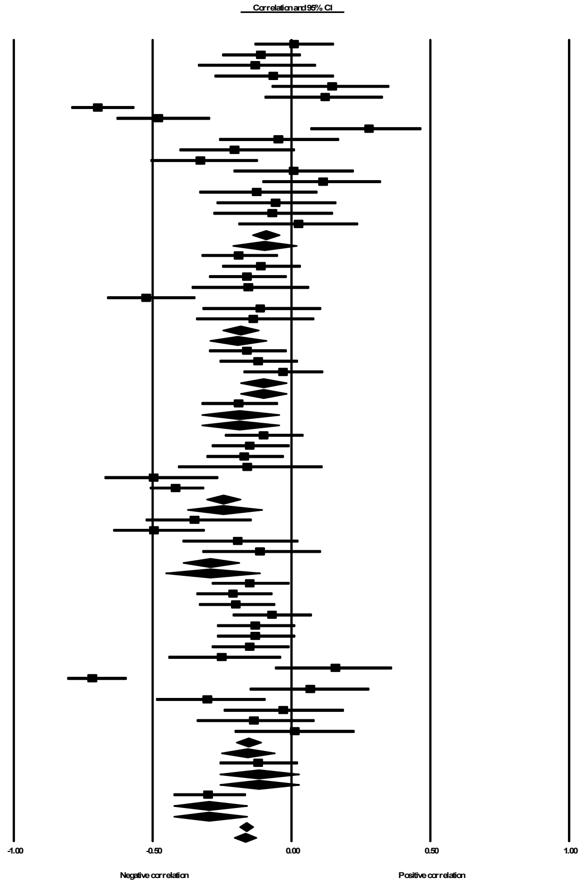

Online supplementary Figure S2. *Correlation between cognitive function and Mn exposure, as a function of the cognitive domain assessed. Note: a negative correlation indicates that the higher the Mn levels, the worse the cognitive function. A random effects model is used to combine studies within each subgroup. A fixed effect model is used to combine subgroups and yield the overall effect. The study-to-study variance (tau-squared) is NOT assumed to be the same for all subgroups - this value is computed within subgroups and NOT pooled across subgroups.*

Model	Group by Subgroup within study	Study name	Subgroup within study	Correlation	Lower limit	Upper limit	Z-Value	p-Value
	Air Mn	Bowler et al., 2015 Auditory Consonant Trigrams 18' delay z-score. Air Mn	Air Mn	-0.130	-0.269	0.014	-1.769	0.077
	Air Mn	Bowler et al., 2015 Auditory Consonant Trigrams 3' delay z-score. Air Mn	Air Mn	-0.070	-0.212	0.075	-0.948	0.343
	Air Mn	Bowler et al., 2015 Auditory Consonant Trigrams 9' delay z-score. Air Mn	Air Mn	-0.130	-0.269	0.014	-1.769	0.077
	Air Mn	Bowler et al., 2015 Auditory Consonant Trigrams mean of 3' , 90' , 18' z. Air Mn	Air Mn	-0.150	-0.288	-0.006	-2.045	0.041
	Air Mn	Bowler et al., 2015 NAB, Daily Living Memory Delayed Recall T-score. Air Mn	Air Mn	-0.170	-0.306	-0.027	-2.322	0.020
	Air Mn	Bowler et al., 2015 NAB, Daily Living Memory Immediate Recall T-score. Air Mn	Air Mn	-0.150	-0.288	-0.006	-2.045	0.041
	Air Mn	Bowler et al., 2015 Neuropsychological Assesment Battery (NAB), Memory index. Air Mn	Air Mn	-0.100	-0.240	0.045	-1.357	0.175
	Air Mn	Bowler et al., 2015 Rey Osterrieth Complex Figure test (ROCF), copy raw score. Air Mn	Air Mn	-0.150	-0.288	-0.006	-2.045	0.041
	Air Mn	Bowler et al., 2015 ROCF, Delayed T. Air Mn	Air Mn	-0.200	-0.334	-0.058	-2.743	0.006
	Air Mn	Bowler et al., 2015 ROCF, Inmedate T. Air Mn	Air Mn	-0.210	-0.344	-0.068	-2.884	0.004
	Air Mn	Bowler et al., 2015 Stroop Color Word test T-score. Air Mn	Air Mn	-0.160	-0.297	-0.017	-2.183	0.029
	Air Mn	Bowler et al., 2015 Stroop Color Word test, Color score. Air Mn	Air Mn	-0.030	-0.173	0.114	-0.406	0.685
	Air Mn	Bowler et al., 2015 Stroop Color Word test, Word score. Air Mn	Air Mn	-0.120	-0.259	0.024	-1.631	0.103
	Air Mn	Bowler et al., 2015 Trail Making Test A, T-score. Air Mn	Air Mn	0.010	-0.134	0.154	0.135	0.892
	Air Mn	Bowler et al., 2015 Trail Making Test B, T-score. Air Mn	Air Mn	-0.110	-0.250	0.034	-1.494	0.135
	Air Mn	Bowler et al., 2015 WAIS III, Animal Naming Scaled Score. Air Mn	Air Mn	-0.190	-0.325	-0.047	-2.602	0.009
	Air Mn	Bowler et al., 2015 WAIS III, Digit backward z-score. Air Mn	Air Mn	-0.160	-0.297	-0.017	-2.183	0.029
	Air Mn	Bowler et al., 2015 WAIS III, Digit forward z-score. Air Mn	Air Mn	-0.110	-0.250	0.034	-1.494	0.135
	Air Mn	Bowler et al., 2015 WAIS III, Digit Symbol Scaled Score. Air Mn	Air Mn	-0.120	-0.259	0.024	-1.631	0.103
	Air Mn	Bowler et al., 2015 WAIS III, Similarities Scaled Score. Air Mn	Air Mn	-0.300	-0.426	-0.163	-4.187	0.000
	Air Mn	Bowler et al., 2015 Weschler Intelligence Scale for Adults, third version (WAIS III), Digit Span Scaled Score. Air Mn	Air Mn	-0.190	-0.325	-0.047	-2.602	0.009
Fixed	Air Mn			-0.141	-0.171	-0.109	-8.773	0.000
Random	Air Mn			-0.141	-0.171	-0.109	-8.773	0.000
	Axilary Hair Mn	Viana et al., 2014 Corsi Block-Tapping Task. Axilary Hair Mn	Axilary Hair Mn	-0.717	-0.808	-0.592	-8.012	0.000
	Axilary Hair Mn	Viana et al., 2014 RAVLT. Axilary Hair Mn	Axilary Hair Mn	0.068	-0.151	0.281	0.605	0.545
	Axilary Hair Mn	Viana et al., 2014 TAVIS-3, Selective attention. Axilary Hair Mn	Axilary Hair Mn	0.280	0.067	0.469	2.557	0.011
	Axilary Hair Mn	Viana et al., 2014 TAVIS-3, Sustained attention. Axilary Hair Mn	Axilary Hair Mn	-0.047	-0.261	0.172	-0.418	0.676
	Axilary Hair Mn	Viana et al., 2014 Trail Making Test A (seconds). Axilary Hair Mn	Axilary Hair Mn	-0.697	-0.794	-0.566	-7.657	0.000
	Axilary Hair Mn	Viana et al., 2014 Trail Making Test B (seconds). Axilary Hair Mn	Axilary Hair Mn	-0.479	-0.630	-0.292	-4.637	0.000
	Axilary Hair Mn	Viana et al., 2014 WAIS III, Digit Span. Axilary Hair Mn	Axilary Hair Mn	-0.523	-0.665	-0.345	-5.159	0.000
	Axilary Hair Mn	Viana et al., 2014 WAIS III. Axilary Hair Mn	Axilary Hair Mn	-0.495	-0.643	-0.311	-4.823	0.000
Fixed	Axilary Hair Mn			-0.369	-0.434	-0.300	-9.738	0.000
Random	Axilary Hair Mn			-0.369	-0.599	-0.083	-2.497	0.013
	Blood Mn	Iqbal et al., 2018 Mini-Mental State Examination. Blood Mn	Blood Mn	-0.417	-0.510	-0.314	-7.297	0.000
Fixed	Blood Mn			-0.417	-0.510	-0.314	-7.297	0.000
Random	Blood Mn			-0.417	-0.510	-0.314	-7.297	0.000
	Hand Fingernails Mn	Ghazali et al., 2013 Mini-Mental State Examination. Hand Fingernails Mn	Hand Fingernails Mn	-0.159	-0.409	0.114	-1.145	0.252
	Hand Fingernails Mn	Ghazali et al., 2013 Montreal Cognitive Assessment. Hand Fingernails Mn	Hand Fingernails Mn	-0.496	-0.674	-0.263	-3.885	0.000
	Hand Fingernails Mn	Viana et al., 2014 Corsi Block-Tapping Task. Hand Fingernails Mn	Hand Fingernails Mn	-0.303	-0.488	-0.092	-2.780	0.005
	Hand Fingernails Mn	Viana et al., 2014 RAVLT. Hand Fingernails Mn	Hand Fingernails Mn	-0.029	-0.244	0.189	-0.258	0.797
	Hand Fingernails Mn	Viana et al., 2014 TAVIS-3, Selective attention. Hand Fingernails Mn	Hand Fingernails Mn	0.008	-0.209	0.225	0.071	0.943
	Hand Fingernails Mn	Viana et al., 2014 TAVIS-3, Sustained attention. Hand Fingernails Mn	Hand Fingernails Mn	0.114	-0.106	0.323	1.018	0.309
	Hand Fingernails Mn	Viana et al., 2014 Trail Making Test A (seconds). Hand Fingernails Mn	Hand Fingernails Mn	-0.205	-0.404	0.013	-1.848	0.065
	Hand Fingernails Mn	Viana et al., 2014 Trail Making Test B (seconds). Hand Fingernails Mn	Hand Fingernails Mn	-0.328	-0.509	-0.119	-3.027	0.002
	Hand Fingernails Mn	Viana et al., 2014 WAIS III, Digit Span. Hand Fingernails Mn	Hand Fingernails Mn	-0.112	-0.321	0.108	-1.000	0.317
	Hand Fingernails Mn	Viana et al., 2014 WAIS III. Hand Fingernails Mn	Hand Fingernails Mn	-0.193	-0.394	0.025	-1.737	0.082
Fixed	Hand Fingernails Mn			-0.163	-0.233	-0.092	-4.463	0.000
Random	Hand Fingernails Mn			-0.170	-0.278	-0.058	-2.967	0.003
	Saliva Mn	Viana et al., 2014 Corsi Block-Tapping Task. Saliva Mn	Saliva Mn	-0.135	-0.342	0.084	-1.207	0.227
	Saliva Mn	Viana et al., 2014 RAVLT. Saliva Mn	Saliva Mn	0.012	-0.206	0.228	0.107	0.915
	Saliva Mn	Viana et al., 2014 TAVIS-3, Selective attention. Saliva Mn	Saliva Mn	-0.069	-0.282	0.150	-0.614	0.539
	Saliva Mn	Viana et al., 2014 TAVIS-3, Sustained attention. Saliva Mn	Saliva Mn	0.026	-0.192	0.242	0.231	0.817
	Saliva Mn	Viana et al., 2014 Trail Making Test A (seconds). Saliva Mn	Saliva Mn	-0.125	-0.333	0.095	-1.117	0.264
	Saliva Mn	Viana et al., 2014 Trail Making Test B (seconds). Saliva Mn	Saliva Mn	-0.057	-0.271	0.162	-0.507	0.612
	Saliva Mn	Viana et al., 2014 WAIS III, Digit Span. Saliva Mn	Saliva Mn	-0.137	-0.344	0.082	-1.225	0.220
	Saliva Mn	Viana et al., 2014 WAIS III. Saliva Mn	Saliva Mn	-0.113	-0.322	0.107	-1.009	0.313
Fixed	Saliva Mn			-0.075	-0.152	0.003	-1.889	0.059
Random	Saliva Mn			-0.075	-0.152	0.003	-1.889	0.059
	Scalp Hair Mn	Viana et al., 2014 Corsi Block-Tapping Task. Scalp Hair Mn	Scalp Hair Mn	-0.251	-0.444	-0.036	-2.280	0.023
	Scalp Hair Mn	Viana et al., 2014 Rey Auditory Verbal Learning Test (RAVLT). Scalp Hair Mn	Scalp Hair Mn	0.159	-0.060	0.363	1.425	0.154
	Scalp Hair Mn	Viana et al., 2014 TAVIS-3, Sustained attention. Scalp Hair Mn	Scalp Hair Mn	0.122	-0.098	0.330	1.090	0.276
	Scalp Hair Mn	Viana et al., 2014 Trail Making Test A (seconds). Scalp Hair Mn	Scalp Hair Mn	-0.130	-0.337	0.090	-1.162	0.245
	Scalp Hair Mn	Viana et al., 2014 Trail Making Test B (seconds). Scalp Hair Mn	Scalp Hair Mn	-0.065	-0.278	0.154	-0.579	0.563
	Scalp Hair Mn	Viana et al., 2014 Visual Attention Computerized Test, third version (TAVIS-3), Selective attention. Scalp Hair Mn	Scalp Hair Mn	0.147	-0.072	0.353	1.316	0.188
	Scalp Hair Mn	Viana et al., 2014 WAIS III, Digit Span. Scalp Hair Mn	Scalp Hair Mn	-0.155	-0.360	0.064	-1.389	0.165
	Scalp Hair Mn	Viana et al., 2014 WAIS III. Scalp Hair Mn	Scalp Hair Mn	-0.349	-0.526	-0.143	-3.238	0.001
Fixed	Scalp Hair Mn			-0.068	-0.145	0.010	-1.703	0.089
Random	Scalp Hair Mn			-0.068	-0.201	0.068	-0.975	0.330
Fixed	Overall			-0.165	-0.188	-0.141	-13.636	0.000
Random	Overall			-0.149	-0.175	-0.122	-10.896	0.000

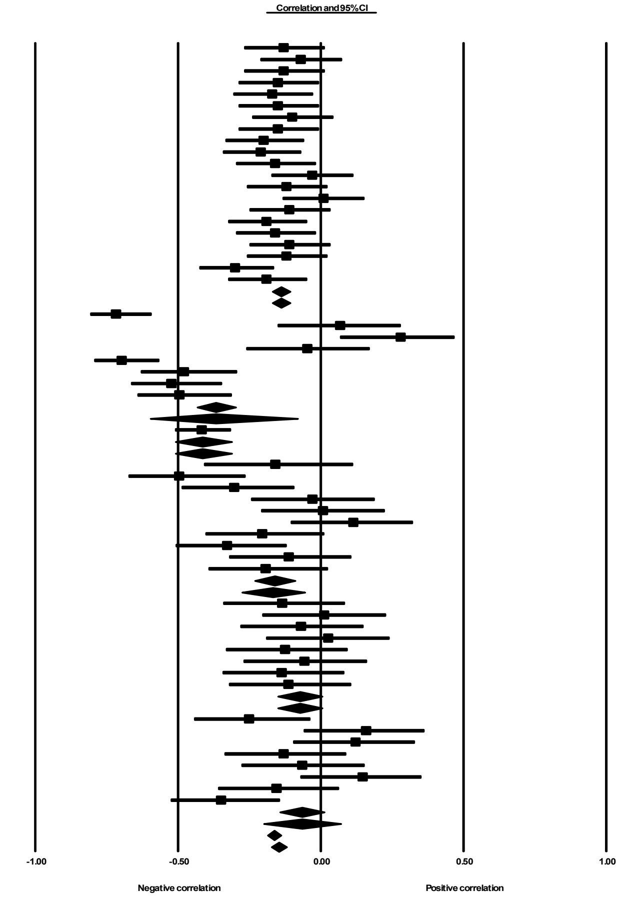

Online supplementary Figure S3. *Correlation between cognitive function and Mn exposure, as a function of the type of exposure to Mn evaluated. Note: a negative correlation indicates that the higher the Mn levels, the worse the cognitive function. A random effects model is used to combine studies within each subgroup. A fixed effect model is used to combine subgroups and yield the overall effect. The study-to-study variance (tau-squared) is NOT assumed to be the same for all subgroups - this value is computed within subgroups and NOT pooled across subgroups.*

Model	Study name	Std diff in means	Standard error	Variance	Lower limit	Upper limit	Z-Value	p-Value
	Bowler et al., 2012 Weschler Intelligence Scale for Adults, third version (WAIS III), Similarities Scaled Score	-0.137	0.145	0.021	-0.422	0.148	-0.943	0.346
	Bowler et al., 2012 Unified Parkinson's Disease Rating Scale (UPDRS), Activities of daily living (ADL)	-0.104	0.146	0.021	-0.390	0.181	-0.716	0.474
	Lucchini et al., 2014 Trail Making Test A, corrected	-0.018	0.128	0.016	-0.268	0.233	-0.137	0.891
	Lucchini et al., 2014 Trail Making Test B, corrected	-0.087	0.128	0.016	-0.338	0.164	-0.680	0.496
	Lucchini et al., 2014 Trail Making Test A, equiv score	0.000	0.128	0.016	-0.251	0.251	0.000	1.000
	Lucchini et al., 2014 Trail Making Test B, equiv score	0.000	0.128	0.016	-0.251	0.251	0.000	1.000
	Lucchini et al., 2014 WAIS III, Digit Span forward	0.000	0.129	0.017	-0.253	0.253	0.000	1.000
	Lucchini et al., 2014 WAIS III, Digit Span backward	0.000	0.129	0.017	-0.253	0.253	0.000	1.000
	Lucchini et al., 2014 WAIS III, Digit Span, correct total score	0.000	0.129	0.017	-0.253	0.253	0.000	1.000
	Lucchini et al., 2014 WAIS III, Digit Symbol, raw score	-0.199	0.129	0.017	-0.452	0.054	-1.544	0.123
	Lucchini et al., 2014 WAIS III, Digit Symbol, correct score	-0.298	0.129	0.017	-0.552	-0.045	-2.305	0.021
	Lucchini et al., 2014 Story Recall Test, raw score	-0.049	0.128	0.016	-0.300	0.202	-0.384	0.701
	Lucchini et al., 2014 Story Recall Test, correct score	0.000	0.128	0.016	-0.251	0.251	0.000	1.000
	Lucchini et al., 2014 Story Recall Test, equiv score	0.000	0.128	0.016	-0.251	0.251	0.000	1.000
	Lucchini et al., 2014 Raven's Colored Progressive Matrices (CPM) Test, raw score	-0.211	0.128	0.016	-0.462	0.040	-1.650	0.099
	Lucchini et al., 2014 CPM Test, percentile	-0.025	0.128	0.016	-0.276	0.226	-0.194	0.847
	Lucchini et al., 2014 Mini-Mental State Examination (MMSE), raw score	0.000	0.128	0.016	-0.251	0.251	0.000	1.000
	Lucchini et al., 2014 MMSE, correct score	0.068	0.128	0.016	-0.183	0.319	0.531	0.595
	Viana et al., 2014 Trail Making Test A (seconds)	0.371	0.223	0.050	-0.067	0.809	1.662	0.096
	Viana et al., 2014 Trail Making Test B (seconds)	-0.187	0.228	0.052	-0.634	0.260	-0.819	0.413
	Viana et al., 2014 WAIS III	0.655	0.228	0.052	0.208	1.103	2.869	0.004
	Viana et al., 2014 WAIS III, Digit Span	0.062	0.221	0.049	-0.371	0.494	0.279	0.780
	Viana et al., 2014 Corsi Block-Tapping Task	0.396	0.220	0.048	-0.034	0.826	1.803	0.071
	Viana et al., 2014 Rey Auditory Verbal Learning Test (RAVLT)	-0.433	0.232	0.054	-0.888	0.021	-1.868	0.062
	Viana et al., 2014 Visual Attention Computerized Test, third version (TAVIS-3), Selective attention	0.000	0.221	0.049	-0.434	0.434	0.000	1.000
	Viana et al., 2014 TAVIS-3, Sustained attention	-0.342	0.222	0.049	-0.776	0.093	-1.539	0.124
Fixed		-0.041	0.029	0.001	-0.097	0.015	-1.426	0.154
Random		-0.038	0.033	0.001	-0.102	0.025	-1.178	0.239

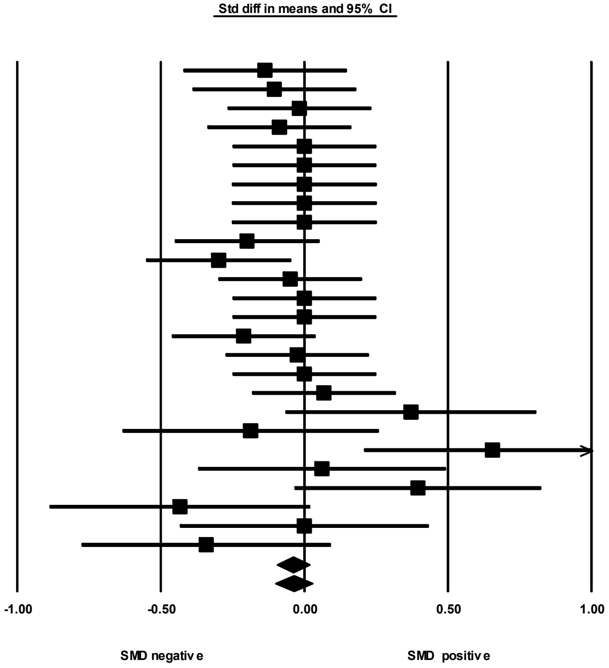

Online supplementary Figure S4. *Cognitive function. Standardized mean differences (SMDs) between groups, prioritizing the use of medians versus means (when both medians and means were reported). All tests and all exposures to Mn. Note: a negative SMD indicates that the group with higher Mn levels had worse cognitive function on average.*

Model	Group by Subgroup within study	Study name	Subgroup within study	Std diff in means	Standard error	Variance	Lower limit	Upper limit	Z-Value	p-Value
	Corsi Block-Tapping Task	Viana et al., 2014 Corsi Block-Tapping Task	Corsi Block-Tapping Task	0.396	0.221	0.049	-0.037	0.829	1.792	0.073
Fixed	Corsi Block-Tapping Task			0.396	0.221	0.049	-0.037	0.829	1.792	0.073
Random	Corsi Block-Tapping Task			0.396	0.221	0.049	-0.037	0.829	1.792	0.073
	CPM	Lucchini et al., 2014 Raven's Colored Progressive Matrices (CPM) Test, raw score	CPM	-0.122	0.128	0.016	-0.373	0.128	-0.957	0.338
	CPM	Lucchini et al., 2014 CPM Test, percentile	CPM	-0.132	0.128	0.016	-0.383	0.119	-1.032	0.302
Fixed	CPM			-0.127	0.090	0.008	-0.305	0.050	-1.407	0.159
Random	CPM			-0.127	0.090	0.008	-0.305	0.050	-1.407	0.159
	MMSE	Lucchini et al., 2014 Mini-Mental State Examination (MMSE), raw score	MMSE	0.013	0.128	0.016	-0.238	0.263	0.101	0.920
	MMSE	Lucchini et al., 2014 MMSE, correct score	MMSE	0.075	0.128	0.016	-0.175	0.326	0.590	0.555
Fixed	MMSE			0.044	0.090	0.008	-0.133	0.221	0.489	0.625
Random	MMSE			0.044	0.090	0.008	-0.133	0.221	0.489	0.625
	RAVLT	Viana et al., 2014 Rey Auditory Verbal Learning Test (RAVLT)	RAVLT	-0.433	0.221	0.049	-0.867	0.000	-1.958	0.050
Fixed	RAVLT			-0.433	0.221	0.049	-0.867	0.000	-1.958	0.050
Random	RAVLT			-0.433	0.221	0.049	-0.867	0.000	-1.958	0.050
	Story Recall Test	Lucchini et al., 2014 Story Recall Test, raw score	Story Recall Test	-0.142	0.128	0.016	-0.393	0.109	-1.107	0.268
	Story Recall Test	Lucchini et al., 2014 Story Recall Test, correct score	Story Recall Test	-0.013	0.128	0.016	-0.264	0.238	-0.102	0.919
	Story Recall Test	Lucchini et al., 2014 Story Recall Test, equiv score	Story Recall Test	0.040	0.128	0.016	-0.211	0.291	0.313	0.754
Fixed	Story Recall Test			-0.038	0.074	0.005	-0.183	0.107	-0.517	0.605
Random	Story Recall Test			-0.038	0.074	0.005	-0.183	0.107	-0.517	0.605
	TAVIS-3	Viana et al., 2014 Visual Attention Computerized Test, third version (TAVIS-3), Selective attention	TAVIS-3	0.000	0.221	0.049	-0.434	0.434	0.000	1.000
	TAVIS-3	Viana et al., 2014 TAVIS-3, Sustained attention	TAVIS-3	-0.342	0.223	0.050	-0.779	0.096	-1.531	0.126
Fixed	TAVIS-3			-0.170	0.157	0.025	-0.478	0.138	-1.079	0.281
Random	TAVIS-3			-0.170	0.171	0.029	-0.504	0.165	-0.994	0.320
	Trail Making Test	Lucchini et al., 2014 Trail Making Test A, corrected	Trail Making Test	-0.064	0.128	0.016	-0.315	0.187	-0.500	0.617
	Trail Making Test	Lucchini et al., 2014 Trail Making Test B, corrected	Trail Making Test	-0.099	0.128	0.016	-0.350	0.151	-0.776	0.438
	Trail Making Test	Lucchini et al., 2014 Trail Making Test A, equiv score	Trail Making Test	-0.140	0.128	0.016	-0.391	0.111	-1.094	0.274
	Trail Making Test	Lucchini et al., 2014 Trail Making Test B, equiv score	Trail Making Test	-0.060	0.128	0.016	-0.311	0.191	-0.469	0.639
	Trail Making Test	Viana et al., 2014 Trail Making Test A (seconds)	Trail Making Test	0.371	0.225	0.051	-0.070	0.812	1.650	0.099
	Trail Making Test	Viana et al., 2014 Trail Making Test B (seconds)	Trail Making Test	-0.187	0.229	0.052	-0.635	0.261	-0.818	0.413
Fixed	Trail Making Test			-0.065	0.059	0.004	-0.181	0.051	-1.096	0.273
Random	Trail Making Test			-0.065	0.059	0.004	-0.181	0.051	-1.096	0.273
	UPDRS	Bowler et al., 2012 Unified Parkinson's Disease Rating Scale (UPDRS), Activities of daily living (ADL)	UPDRS	-0.104	0.146	0.021	-0.390	0.181	-0.716	0.474
Fixed	UPDRS			-0.104	0.146	0.021	-0.390	0.181	-0.716	0.474
Random	UPDRS			-0.104	0.146	0.021	-0.390	0.181	-0.716	0.474
	WAIS III	Bowler et al., 2012 Weschler Intelligence Scale for Adults, third version (WAIS III), Similarities Scaled Score	WAIS III	-0.137	0.145	0.021	-0.422	0.148	-0.943	0.346
	WAIS III	Lucchini et al., 2014 WAIS III, Digit Span forward	WAIS III	0.071	0.129	0.017	-0.182	0.324	0.551	0.582
	WAIS III	Lucchini et al., 2014 WAIS III, Digit Span backward	WAIS III	-0.011	0.129	0.017	-0.264	0.242	-0.085	0.932
	WAIS III	Lucchini et al., 2014 WAIS III, Digit Span, correct total score	WAIS III	-0.016	0.129	0.017	-0.268	0.237	-0.121	0.904
	WAIS III	Lucchini et al., 2014 WAIS III, Digit Symbol, raw score	WAIS III	-0.115	0.129	0.017	-0.368	0.138	-0.890	0.373
	WAIS III	Lucchini et al., 2014 WAIS III, Digit Symbol, correct score	WAIS III	-0.254	0.129	0.017	-0.507	0.000	-1.960	0.050
	WAIS III	Viana et al., 2014 WAIS III	WAIS III	0.438	0.224	0.050	-0.000	0.877	1.958	0.050
	WAIS III	Viana et al., 2014 WAIS III, Digit Span	WAIS III	0.062	0.219	0.048	-0.367	0.490	0.281	0.778
Fixed	WAIS III			-0.041	0.051	0.003	-0.140	0.059	-0.802	0.422
Random	WAIS III			-0.035	0.059	0.003	-0.150	0.080	-0.595	0.552
Fixed	Overall			-0.052	0.029	0.001	-0.108	0.004	-1.824	0.068
Random	Overall			-0.051	0.030	0.001	-0.109	0.008	-1.706	0.088

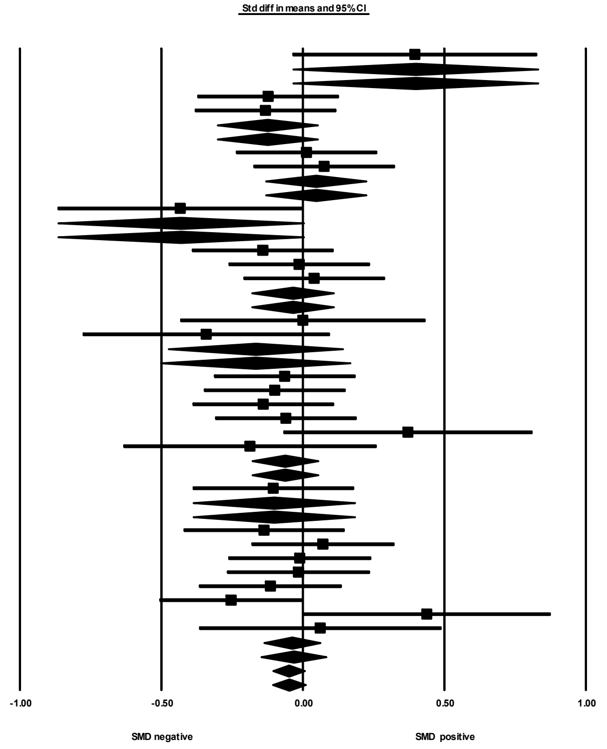

Online supplementary Figure S5. *Cognitive function. Standardized mean differences (SMDs) between groups, as a function of the cognitive test used. Note: a negative SMD indicates that the group with higher Mn levels had worse cognitive function on average. A random effects model is used to combine studies within each subgroup. A fixed effect model is used to combine subgroups and yield the overall effect. The study-to-study variance (tau-squared) is NOT assumed to be the same for all subgroups - this value is computed within subgroups and NOT pooled across subgroups.*

Model	Group by Subgroup within study	Study name	Subgroup within study	Std diff in means	Standard error	Variance	Lower limit	Upper limit	Z-Value	p-Value
	Attention	Lucchini et al., 2014 Trail Making Test A, corrected	Attention	-0.064	0.128	0.016	-0.315	0.187	-0.500	0.617
	Attention	Lucchini et al., 2014 Trail Making Test B, corrected	Attention	-0.099	0.128	0.016	-0.350	0.151	-0.776	0.438
	Attention	Lucchini et al., 2014 Trail Making Test A, equiv score	Attention	-0.140	0.128	0.016	-0.391	0.111	-1.094	0.274
	Attention	Lucchini et al., 2014 Trail Making Test B, equiv score	Attention	-0.060	0.128	0.016	-0.311	0.191	-0.469	0.639
	Attention	Viana et al., 2014 Trail Making Test A (seconds)	Attention	0.371	0.225	0.051	-0.070	0.812	1.650	0.099
	Attention	Viana et al., 2014 Trail Making Test B (seconds)	Attention	-0.187	0.229	0.052	-0.635	0.261	-0.818	0.413
	Attention	Viana et al., 2014 Visual Attention Computerized Test, third version (TAVIS-3), Selective attention	Attention	0.000	0.221	0.049	-0.434	0.434	0.000	1.000
	Attention	Viana et al., 2014 TAVIS-3, Sustained attention	Attention	-0.342	0.223	0.050	-0.779	0.096	-1.531	0.126
Fixed	Attention			-0.078	0.056	0.003	-0.187	0.031	-1.406	0.160
Random	Attention			-0.078	0.056	0.003	-0.187	0.031	-1.406	0.160
	Attention and Working Memory	Lucchini et al., 2014 WAIS III, Digit Span forward	Attention and Working Memory	0.071	0.129	0.017	-0.182	0.324	0.551	0.582
	Attention and Working Memory	Lucchini et al., 2014 WAIS III, Digit Span backward	Attention and Working Memory	-0.011	0.129	0.017	-0.264	0.242	-0.085	0.932
	Attention and Working Memory	Lucchini et al., 2014 WAIS III, Digit Span, correct total score	Attention and Working Memory	-0.016	0.129	0.017	-0.268	0.237	-0.121	0.904
	Attention and Working Memory	Viana et al., 2014 WAIS III, Digit Span	Attention and Working Memory	0.062	0.219	0.048	-0.367	0.490	0.281	0.778
Fixed	Attention and Working Memory			0.020	0.071	0.005	-0.119	0.158	0.279	0.780
Random	Attention and Working Memory			0.020	0.071	0.005	-0.119	0.158	0.279	0.780
	General Cognitive Functioning	Bowler et al., 2012 Unified Parkinson's Disease Rating Scale (UPDRS), Activities of daily living (ADL)	General Cognitive Functioning	-0.104	0.146	0.021	-0.390	0.181	-0.716	0.474
	General Cognitive Functioning	Lucchini et al., 2014 Raven's Colored Progressive Matrices (CPM) Test, raw score	General Cognitive Functioning	-0.122	0.128	0.016	-0.373	0.128	-0.957	0.338
	General Cognitive Functioning	Lucchini et al., 2014 CPM Test, percentile	General Cognitive Functioning	-0.132	0.128	0.016	-0.383	0.119	-1.032	0.302
	General Cognitive Functioning	Lucchini et al., 2014 Mini-Mental State Examination (MMSE), raw score	General Cognitive Functioning	0.013	0.128	0.016	-0.238	0.263	0.101	0.920
	General Cognitive Functioning	Lucchini et al., 2014 MMSE, correct score	General Cognitive Functioning	0.075	0.128	0.016	-0.175	0.326	0.590	0.555
Fixed	General Cognitive Functioning			-0.052	0.059	0.003	-0.166	0.063	-0.882	0.378
Random	General Cognitive Functioning			-0.052	0.059	0.003	-0.166	0.063	-0.882	0.378
	Intelligence	Viana et al., 2014 WAIS III	Intelligence	0.438	0.224	0.050	-0.000	0.877	1.958	0.050
Fixed	Intelligence			0.438	0.224	0.050	-0.000	0.877	1.958	0.050
Random	Intelligence			0.438	0.224	0.050	-0.000	0.877	1.958	0.050
	Memory	Lucchini et al., 2014 Story Recall Test, raw score	Memory	-0.142	0.128	0.016	-0.393	0.109	-1.107	0.268
	Memory	Lucchini et al., 2014 Story Recall Test, correct score	Memory	-0.013	0.128	0.016	-0.264	0.238	-0.102	0.919
	Memory	Lucchini et al., 2014 Story Recall Test, equiv score	Memory	0.040	0.128	0.016	-0.211	0.291	0.313	0.754
	Memory	Viana et al., 2014 Corsi Block-Tapping Task	Memory	0.396	0.221	0.049	-0.037	0.829	1.792	0.073
	Memory	Viana et al., 2014 Rey Auditory Verbal Learning Test (RAVLT)	Memory	-0.433	0.221	0.049	-0.867	0.000	-1.958	0.050
Fixed	Memory			-0.034	0.067	0.004	-0.165	0.096	-0.516	0.606
Random	Memory			-0.033	0.099	0.010	-0.227	0.162	-0.330	0.741
	Proccesing Speed	Lucchini et al., 2014 WAIS III, Digit Symbol, raw score	Proccesing Speed	-0.115	0.129	0.017	-0.368	0.138	-0.890	0.373
	Proccesing Speed	Lucchini et al., 2014 WAIS III, Digit Symbol, correct score	Proccesing Speed	-0.254	0.129	0.017	-0.507	0.000	-1.960	0.050
Fixed	Proccesing Speed			-0.184	0.091	0.008	-0.363	-0.005	-2.014	0.044
Random	Proccesing Speed			-0.184	0.091	0.008	-0.363	-0.005	-2.014	0.044
	Verbal Comprehension	Bowler et al., 2012 Weschler Intelligence Scale for Adults, third version (WAIS III), Similarities Scaled Score	Verbal Comprehension	-0.137	0.145	0.021	-0.422	0.148	-0.943	0.346
Fixed	Verbal Comprehension			-0.137	0.145	0.021	-0.422	0.148	-0.943	0.346
Random	Verbal Comprehension			-0.137	0.145	0.021	-0.422	0.148	-0.943	0.346
Fixed	Overall			-0.052	0.029	0.001	-0.108	0.004	-1.824	0.068
Random	Overall			-0.054	0.030	0.001	-0.113	0.005	-1.790	0.073

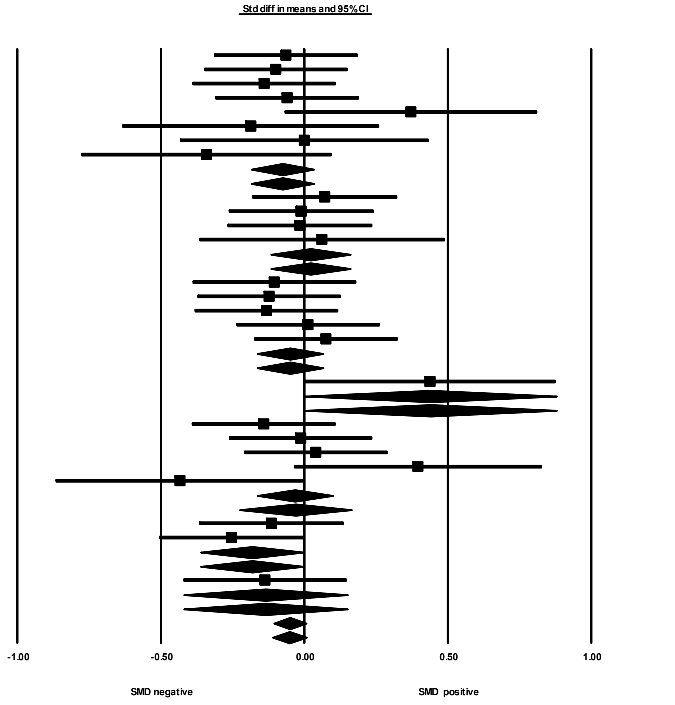

Online supplementary Figure S6. *Cognitive function. Standardized mean differences (SMDs) between groups, as a function of the cognitive domain assessed. Note: a negative SMD indicates that the group with higher Mn levels had worse cognitive function on average. A random effects model is used to combine studies within each subgroup. A fixed effect model is used to combine subgroups and yield the overall effect. The study-to-study variance (tau-squared) is NOT assumed to be the same for all subgroups - this value is computed within subgroups and NOT pooled across subgroups.*

Model	Group by Subgroup within study	Study name	Subgroup within study	Std diff in means	Standard error	Variance	Lower limit	Upper limit	Z-Value	p-Value
	Air Mn & Blood	Bowler et al., 2012 Weschler Intelligence Scale for Adults, third version (WAIS III), Similarities Scaled Score	Air Mn & Blood	-0.137	0.145	0.021	-0.422	0.148	-0.943	0.346
	Air Mn & Blood	Bowler et al., 2012 Unified Parkinson's Disease Rating Scale (UPDRS), Activities of daily living (ADL)	Air Mn & Blood	-0.104	0.146	0.021	-0.390	0.181	-0.716	0.474
Fixed	Air Mn & Blood			-0.121	0.103	0.011	-0.323	0.081	-1.173	0.241
Random	Air Mn & Blood			-0.121	0.103	0.011	-0.323	0.081	-1.173	0.241
	Air Mn, Blood & Urine	Lucchini et al., 2014 Trail Making Test A, corrected	Air Mn, Blood & Urine	-0.064	0.128	0.016	-0.315	0.187	-0.500	0.617
	Air Mn, Blood & Urine	Lucchini et al., 2014 Trail Making Test B, corrected	Air Mn, Blood & Urine	-0.099	0.128	0.016	-0.350	0.151	-0.776	0.438
	Air Mn, Blood & Urine	Lucchini et al., 2014 Trail Making Test A, equiv score	Air Mn, Blood & Urine	-0.140	0.128	0.016	-0.391	0.111	-1.094	0.274
	Air Mn, Blood & Urine	Lucchini et al., 2014 Trail Making Test B, equiv score	Air Mn, Blood & Urine	-0.060	0.128	0.016	-0.311	0.191	-0.469	0.639
	Air Mn, Blood & Urine	Lucchini et al., 2014 WAIS III, Digit Span forward	Air Mn, Blood & Urine	0.071	0.129	0.017	-0.182	0.324	0.551	0.582
	Air Mn, Blood & Urine	Lucchini et al., 2014 WAIS III, Digit Span backward	Air Mn, Blood & Urine	-0.011	0.129	0.017	-0.264	0.242	-0.085	0.932
	Air Mn, Blood & Urine	Lucchini et al., 2014 WAIS III, Digit Span, correct total score	Air Mn, Blood & Urine	-0.016	0.129	0.017	-0.268	0.237	-0.121	0.904
	Air Mn, Blood & Urine	Lucchini et al., 2014 WAIS III, Digit Symbol, raw score	Air Mn, Blood & Urine	-0.115	0.129	0.017	-0.368	0.138	-0.890	0.373
	Air Mn, Blood & Urine	Lucchini et al., 2014 WAIS III, Digit Symbol, correct score	Air Mn, Blood & Urine	-0.254	0.129	0.017	-0.507	0.000	-1.960	0.050
	Air Mn, Blood & Urine	Lucchini et al., 2014 Story Recall Test, raw score	Air Mn, Blood & Urine	-0.142	0.128	0.016	-0.393	0.109	-1.107	0.268
	Air Mn, Blood & Urine	Lucchini et al., 2014 Story Recall Test, correct score	Air Mn, Blood & Urine	-0.013	0.128	0.016	-0.264	0.238	-0.102	0.919
	Air Mn, Blood & Urine	Lucchini et al., 2014 Story Recall Test, equiv score	Air Mn, Blood & Urine	0.040	0.128	0.016	-0.211	0.291	0.313	0.754
	Air Mn, Blood & Urine	Lucchini et al., 2014 Raven's Colored Progressive Matrices (CPM) Test, raw score	Air Mn, Blood & Urine	-0.122	0.128	0.016	-0.373	0.128	-0.957	0.338
	Air Mn, Blood & Urine	Lucchini et al., 2014 CPM Test, percentile	Air Mn, Blood & Urine	-0.132	0.128	0.016	-0.383	0.119	-1.032	0.302
	Air Mn, Blood & Urine	Lucchini et al., 2014 Mini-Mental State Examination (MMSE), raw score	Air Mn, Blood & Urine	0.013	0.128	0.016	-0.238	0.263	0.101	0.920
	Air Mn, Blood & Urine	Lucchini et al., 2014 MMSE, correct score	Air Mn, Blood & Urine	0.075	0.128	0.016	-0.175	0.326	0.590	0.555
Fixed	Air Mn, Blood & Urine			-0.060	0.032	0.001	-0.123	0.002	-1.883	0.060
Random	Air Mn, Blood & Urine			-0.060	0.032	0.001	-0.123	0.002	-1.883	0.060
	Scalp hair, Axilary Hair, Fingernails & Saliva	Viana et al., 2014 Trail Making Test A (seconds)	Scalp hair, Axilary Hair, Fingernails & Saliva	0.371	0.225	0.051	-0.070	0.812	1.650	0.099
	Scalp hair, Axilary Hair, Fingernails & Saliva	Viana et al., 2014 Trail Making Test B (seconds)	Scalp hair, Axilary Hair, Fingernails & Saliva	-0.187	0.229	0.052	-0.635	0.261	-0.818	0.413
	Scalp hair, Axilary Hair, Fingernails & Saliva	Viana et al., 2014 WAIS III	Scalp hair, Axilary Hair, Fingernails & Saliva	0.438	0.224	0.050	-0.000	0.877	1.958	0.050
	Scalp hair, Axilary Hair, Fingernails & Saliva	Viana et al., 2014 WAIS III, Digit Span	Scalp hair, Axilary Hair, Fingernails & Saliva	0.062	0.219	0.048	-0.367	0.490	0.281	0.778
	Scalp hair, Axilary Hair, Fingernails & Saliva	Viana et al., 2014 Corsi Block-Tapping Task	Scalp hair, Axilary Hair, Fingernails & Saliva	0.396	0.221	0.049	-0.037	0.829	1.792	0.073
	Scalp hair, Axilary Hair, Fingernails & Saliva	Viana et al., 2014 Rey Auditory Verbal Learning Test (RAVLT)	Scalp hair, Axilary Hair, Fingernails & Saliva	-0.433	0.221	0.049	-0.867	0.000	-1.958	0.050
	Scalp hair, Axilary Hair, Fingernails & Saliva	Viana et al., 2014 Visual Attention Computerized Test, third version (TAVIS-3), Selective attention	Scalp hair, Axilary Hair, Fingernails & Saliva	0.000	0.221	0.049	-0.434	0.434	0.000	1.000
	Scalp hair, Axilary Hair, Fingernails & Saliva	Viana et al., 2014 TAVIS-3, Sustained attention	Scalp hair, Axilary Hair, Fingernails & Saliva	-0.342	0.223	0.050	-0.779	0.096	-1.531	0.126
Fixed	Scalp hair, Axilary Hair, Fingernails & Saliva			0.038	0.079	0.006	-0.116	0.193	0.488	0.625
Random	Scalp hair, Axilary Hair, Fingernails & Saliva			0.038	0.121	0.015	-0.198	0.275	0.317	0.751
Fixed	Overall			-0.052	0.029	0.001	-0.108	0.004	-1.824	0.068
Random	Overall			-0.059	0.030	0.001	-0.118	-0.001	-2.003	0.045

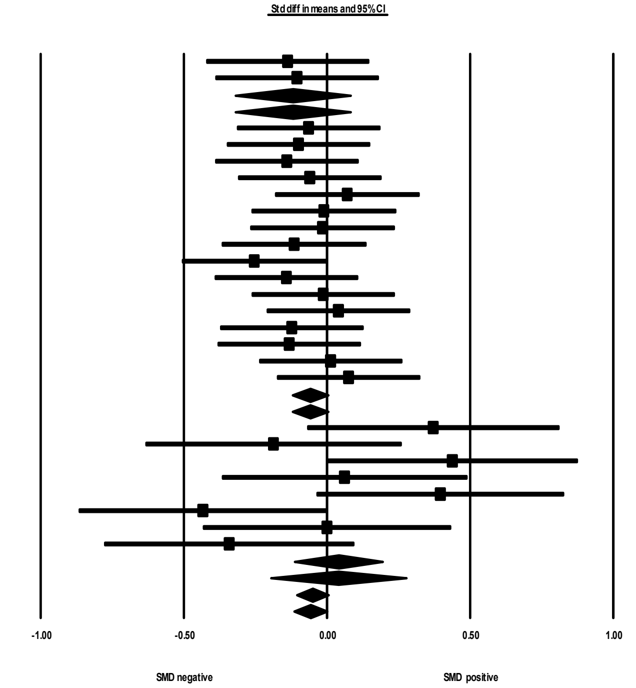

Online supplementary Figure S7. *Cognitive function. Standardized mean differences (SMDs) between groups, as a function of the type of exposure to Mn evaluated. Note: a negative SMD indicates that the group with higher Mn levels had worse cognitive function on average. A random effects model is used to combine studies within each subgroup. A fixed effect model is used to combine subgroups and yield the overall effect. The study-to-study variance (tau-squared) is NOT assumed to be the same for all subgroups - this value is computed within subgroups and NOT pooled across subgroups.*

Model	Group by Subgroup within study	Study name	Subgroup within study	Correlation	Lower limit	Upper limit	Z-Value	p-Value
	CATSYS	Bowler et al., 2016 CATSYS Tremor Intensity, dom. Air Mn	CATSYS	-0.170	-0.306	-0.027	-2.322	0.020
	CATSYS	Bowler et al., 2016 CATSYS Tremor Center frequency, dom. Air Mn	CATSYS	-0.340	-0.461	-0.206	-4.790	0.000
	CATSYS	Bowler et al., 2016 CATSYS Tremor Harmonic index, dom. Air Mn	CATSYS	0.330	0.195	0.452	4.638	0.000
	CATSYS	Bowler et al., 2016 CATSYS Tremor Intensity, non-dom. Air Mn	CATSYS	-0.170	-0.306	-0.027	-2.322	0.020
	CATSYS	Bowler et al., 2016 CATSYS Tremor Center frequency, non-dom. Air Mn	CATSYS	-0.330	-0.452	-0.195	-4.638	0.000
	CATSYS	Bowler et al., 2016 CATSYS Tremor Harmonic index, non-dom. Air Mn	CATSYS	0.360	0.228	0.479	5.098	0.000
Fixed	CATSYS			-0.053	-0.112	0.006	-1.770	0.077
Random	CATSYS			-0.053	-0.307	0.208	-0.397	0.692
	Dynamometer	Bowler et al., 2016 Dynamometer dom T-score. Air Mn	Dynamometer	-0.060	-0.202	0.085	-0.813	0.416
	Dynamometer	Bowler et al., 2016 Dynamometer non-dom T-score. Air Mn	Dynamometer	-0.050	-0.192	0.095	-0.677	0.498
Fixed	Dynamometer			-0.055	-0.156	0.047	-1.053	0.292
Random	Dynamometer			-0.055	-0.156	0.047	-1.053	0.292
	Finger Tapping	Bowler et al., 2016 Finger Tapping dom T-score. Air Mn	Finger Tapping	-0.260	-0.389	-0.121	-3.600	0.000
	Finger Tapping	Bowler et al., 2016 Finger Tapping non-dom T-score. Air Mn	Finger Tapping	-0.160	-0.297	-0.017	-2.183	0.029
Fixed	Finger Tapping			-0.211	-0.306	-0.111	-4.089	0.000
Random	Finger Tapping			-0.211	-0.306	-0.111	-4.082	0.000
	Grooved Pegboard	Bowler et al., 2016 Grooved Pegboard dom T-score. Air Mn	Grooved Pegboard	-0.100	-0.240	0.045	-1.357	0.175
	Grooved Pegboard	Bowler et al., 2016 Grooved Pegboard non-dom T-score. Air Mn	Grooved Pegboard	-0.150	-0.288	-0.006	-2.045	0.041
	Grooved Pegboard	Viana et al., 2014 Grooved Pegboard dom (seconds). Scalp Hair Mn	Grooved Pegboard	-0.223	-0.421	-0.005	-2.003	0.045
	Grooved Pegboard	Viana et al., 2014 Grooved Pegboard non-dom (seconds). Scalp Hair Mn	Grooved Pegboard	-0.127	-0.336	0.094	-1.128	0.259
	Grooved Pegboard	Viana et al., 2014 Grooved Pegboard dom (seconds). Axilary Hair Mn	Grooved Pegboard	-0.530	-0.799	-0.084	-2.286	0.022
	Grooved Pegboard	Viana et al., 2014 Grooved Pegboard non-dom (seconds). Axilary Hair Mn	Grooved Pegboard	-0.618	-0.842	-0.212	-2.795	0.005
	Grooved Pegboard	Viana et al., 2014 Grooved Pegboard dom (seconds). Hand Fingernails Mn	Grooved Pegboard	-0.231	-0.438	-0.001	-1.968	0.049
	Grooved Pegboard	Viana et al., 2014 Grooved Pegboard non-dom (seconds). Hand Fingernails Mn	Grooved Pegboard	-0.100	-0.323	0.133	-0.839	0.401
	Grooved Pegboard	Viana et al., 2014 Grooved Pegboard dom (seconds). Saliva Mn	Grooved Pegboard	-0.125	-0.333	0.095	-1.117	0.264
	Grooved Pegboard	Viana et al., 2014 Grooved Pegboard non-dom (seconds). Saliva Mn	Grooved Pegboard	-0.087	-0.298	0.133	-0.775	0.438
Fixed	Grooved Pegboard			-0.156	-0.221	-0.090	-4.584	0.000
Random	Grooved Pegboard			-0.158	-0.227	-0.088	-4.398	0.000
	Postural balance	Standridge et al., 2008 Postural balance testing (eyes open on the platform), Ln Sway area (cm2). Hair Mn	Postural balance	-0.240	-0.501	0.061	-1.567	0.117
	Postural balance	Standridge et al., 2008 Postural balance testing (eyes closed on the platform), Ln Sway area (cm2). Hair Mn	Postural balance	-0.290	-0.540	0.008	-1.912	0.056
	Postural balance	Standridge et al., 2008 Postural balance testing (eyes open on a 4 inch thick foampad), Ln Sway area (cm2). Hair Mn	Postural balance	-0.320	-0.563	-0.026	-2.124	0.034
	Postural balance	Standridge et al., 2008 Postural balance testing (eyes closed on a 4 inch thick foampad), Ln Sway area (cm2). Hair Mn	Postural balance	-0.240	-0.501	0.061	-1.567	0.117
	Postural balance	Standridge et al., 2008 Postural balance testing (eyes open on the platform), Ln Sway length (cm). Hair Mn	Postural balance	-0.420	-0.637	-0.141	-2.867	0.004
	Postural balance	Standridge et al., 2008 Postural balance testing (eyes closed on the platform), Ln Sway length (cm). Hair Mn	Postural balance	-0.450	-0.659	-0.177	-3.104	0.002
	Postural balance	Standridge et al., 2008 Postural balance testing (eyes open on a 4 inch thick foampad), Ln Sway lenght (cm). Hair Mn	Postural balance	-0.210	-0.477	0.093	-1.365	0.172
	Postural balance	Standridge et al., 2008 Postural balance testing (eyes closed on a 4 inch thick foampad), Ln Sway lenght (cm). Hair Mn	Postural balance	-0.220	-0.485	0.082	-1.432	0.152
	Postural balance	Standridge et al., 2008 Postural balance testing (eyes open on the platform), Ln Sway area (cm2). Blood Mn	Postural balance	-0.160	-0.436	0.144	-1.033	0.301
	Postural balance	Standridge et al., 2008 Postural balance testing (eyes closed on the platform), Ln Sway area (cm2). Blood Mn	Postural balance	-0.110	-0.394	0.193	-0.707	0.479
	Postural balance	Standridge et al., 2008 Postural balance testing (eyes open on a 4 inch thick foampad), Ln Sway area (cm2). Blood Mn	Postural balance	-0.110	-0.394	0.193	-0.707	0.479
	Postural balance	Standridge et al., 2008 Postural balance testing (eyes closed on a 4 inch thick foampad), Ln Sway area (cm2). Blood Mn	Postural balance	0.060	-0.241	0.351	0.385	0.700
	Postural balance	Standridge et al., 2008 Postural balance testing (eyes open on the platform), Ln Sway length (cm). Blood Mn	Postural balance	-0.110	-0.394	0.193	-0.707	0.479
	Postural balance	Standridge et al., 2008 Postural balance testing (eyes closed on the platform), Ln Sway length (cm). Blood Mn	Postural balance	-0.050	-0.342	0.251	-0.320	0.749
	Postural balance	Standridge et al., 2008 Postural balance testing (eyes open on a 4 inch thick foampad), Ln Sway lenght (cm). Blood Mn	Postural balance	0.020	-0.279	0.315	0.128	0.898
	Postural balance	Standridge et al., 2008 Postural balance testing (eyes closed on a 4 inch thick foampad), Ln Sway lenght (cm). Blood Mn	Postural balance	0.060	-0.241	0.351	0.385	0.700
Fixed	Postural balance			-0.179	-0.252	-0.104	-4.629	0.000
Random	Postural balance			-0.179	-0.255	-0.100	-4.427	0.000
Fixed	Overall			-0.122	-0.155	-0.089	-7.086	0.000
Random	Overall			-0.153	-0.194	-0.111	-7.140	0.000

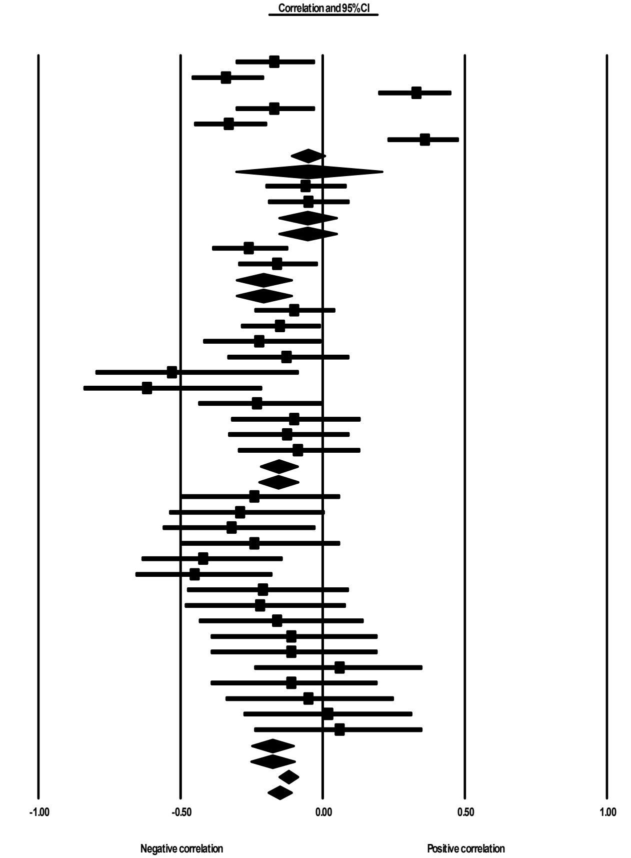

Online supplementary Figure S8. *Correlation between motor function and Mn exposure, as a function of the motor test used.Note: a negative correlation indicates that the higher the Mn levels, the worse the motor function. A random effects model is used to combine studies within each subgroup. A fixed effect model is used to combine subgroups and yield the overall effect. The study-to-study variance (tau-squared) is NOT assumed to be the same for all subgroups - this value is computed within subgroups and NOT pooled across subgroups.*

Model		Study name	Subgroup within study	Correlation	Lower limit	Upper limit	Z-Value	p-Value
	Eye-hand coordination and motor speed	Bowler et al., 2016 Grooved Pegboard dom T-score. Air Mn	Eye-hand coordination and motor speed	-0.100	-0.240	0.045	-1.357	0.175
	Eye-hand coordination and motor speed	Bowler et al., 2016 Grooved Pegboard non-dom T-score. Air Mn	Eye-hand coordination and motor speed	-0.150	-0.288	-0.006	-2.045	0.041
	Eye-hand coordination and motor speed	Viana et al., 2014 Grooved Pegboard dom (seconds). Scalp Hair Mn	Eye-hand coordination and motor speed	-0.223	-0.421	-0.005	-2.003	0.045
	Eye-hand coordination and motor speed	Viana et al., 2014 Grooved Pegboard non-dom (seconds). Scalp Hair Mn	Eye-hand coordination and motor speed	-0.127	-0.336	0.094	-1.128	0.259
	Eye-hand coordination and motor speed	Viana et al., 2014 Grooved Pegboard dom (seconds). Axilary Hair Mn	Eye-hand coordination and motor speed	-0.530	-0.799	-0.084	-2.286	0.022
	Eye-hand coordination and motor speed	Viana et al., 2014 Grooved Pegboard non-dom (seconds). Axilary Hair Mn	Eye-hand coordination and motor speed	-0.618	-0.842	-0.212	-2.795	0.005
	Eye-hand coordination and motor speed	Viana et al., 2014 Grooved Pegboard dom (seconds). Hand Fingernails Mn	Eye-hand coordination and motor speed	-0.231	-0.438	-0.001	-1.968	0.049
	Eye-hand coordination and motor speed	Viana et al., 2014 Grooved Pegboard non-dom (seconds). Hand Fingernails Mn	Eye-hand coordination and motor speed	-0.100	-0.323	0.133	-0.839	0.401
	Eye-hand coordination and motor speed	Viana et al., 2014 Grooved Pegboard dom (seconds). Saliva Mn	Eye-hand coordination and motor speed	-0.125	-0.333	0.095	-1.117	0.264
	Eye-hand coordination and motor speed	Viana et al., 2014 Grooved Pegboard non-dom (seconds). Saliva Mn	Eye-hand coordination and motor speed	-0.087	-0.298	0.133	-0.775	0.438
Fixed	Eye-hand coordination and motor speed			-0.156	-0.221	-0.090	-4.584	0.000
Random	Eye-hand coordination and motor speed			-0.158	-0.227	-0.088	-4.398	0.000
	Hand strength	Bowler et al., 2016 Dynamometer dom T-score. Air Mn	Hand strength	-0.060	-0.202	0.085	-0.813	0.416
	Hand strength	Bowler et al., 2016 Dynamometer non-dom T-score. Air Mn	Hand strength	-0.050	-0.192	0.095	-0.677	0.498
Fixed	Hand strength			-0.055	-0.156	0.047	-1.053	0.292
Random	Hand strength			-0.055	-0.156	0.047	-1.053	0.292
	Postural balance	Standridge et al., 2008 Postural balance testing (eyes open on the platform), Ln Sway area (cm2). Hair Mn	Postural balance	-0.240	-0.501	0.061	-1.567	0.117
	Postural balance	Standridge et al., 2008 Postural balance testing (eyes closed on the platform), Ln Sway area (cm2). Hair Mn	Postural balance	-0.290	-0.540	0.008	-1.912	0.056
	Postural balance	Standridge et al., 2008 Postural balance testing (eyes open on a 4 inch thick foampad), Ln Sway area (cm2). Hair Mn	Postural balance	-0.320	-0.563	-0.026	-2.124	0.034
	Postural balance	Standridge et al., 2008 Postural balance testing (eyes closed on a 4 inch thick foampad), Ln Sway area (cm2). Hair Mn	Postural balance	-0.240	-0.501	0.061	-1.567	0.117
	Postural balance	Standridge et al., 2008 Postural balance testing (eyes open on the platform), Ln Sway length (cm). Hair Mn	Postural balance	-0.420	-0.637	-0.141	-2.867	0.004
	Postural balance	Standridge et al., 2008 Postural balance testing (eyes closed on the platform), Ln Sway length (cm). Hair Mn	Postural balance	-0.450	-0.659	-0.177	-3.104	0.002
	Postural balance	Standridge et al., 2008 Postural balance testing (eyes open on a 4 inch thick foampad), Ln Sway lenght (cm). Hair Mn	Postural balance	-0.210	-0.477	0.093	-1.365	0.172
	Postural balance	Standridge et al., 2008 Postural balance testing (eyes closed on a 4 inch thick foampad), Ln Sway lenght (cm). Hair Mn	Postural balance	-0.220	-0.485	0.082	-1.432	0.152
	Postural balance	Standridge et al., 2008 Postural balance testing (eyes open on the platform), Ln Sway area (cm2). Blood Mn	Postural balance	-0.160	-0.436	0.144	-1.033	0.301
	Postural balance	Standridge et al., 2008 Postural balance testing (eyes closed on the platform), Ln Sway area (cm2). Blood Mn	Postural balance	-0.110	-0.394	0.193	-0.707	0.479
	Postural balance	Standridge et al., 2008 Postural balance testing (eyes open on a 4 inch thick foampad), Ln Sway area (cm2). Blood Mn	Postural balance	-0.110	-0.394	0.193	-0.707	0.479
	Postural balance	Standridge et al., 2008 Postural balance testing (eyes closed on a 4 inch thick foampad), Ln Sway area (cm2). Blood Mn	Postural balance	0.060	-0.241	0.351	0.385	0.700
	Postural balance	Standridge et al., 2008 Postural balance testing (eyes open on the platform), Ln Sway length (cm). Blood Mn	Postural balance	-0.110	-0.394	0.193	-0.707	0.479
	Postural balance	Standridge et al., 2008 Postural balance testing (eyes closed on the platform), Ln Sway length (cm). Blood Mn	Postural balance	-0.050	-0.342	0.251	-0.320	0.749
	Postural balance	Standridge et al., 2008 Postural balance testing (eyes open on a 4 inch thick foampad), Ln Sway lenght (cm). Blood Mn	Postural balance	0.020	-0.279	0.315	0.128	0.898
	Postural balance	Standridge et al., 2008 Postural balance testing (eyes closed on a 4 inch thick foampad), Ln Sway lenght (cm). Blood Mn	Postural balance	0.060	-0.241	0.351	0.385	0.700
Fixed	Postural balance			-0.179	-0.252	-0.104	-4.629	0.000
Random	Postural balance			-0.179	-0.255	-0.100	-4.427	0.000
	Self-directed manual speed	Bowler et al., 2016 Finger Tapping dom T-score. Air Mn	Self-directed manual speed	-0.260	-0.389	-0.121	-3.600	0.000
	Self-directed manual speed	Bowler et al., 2016 Finger Tapping non-dom T-score. Air Mn	Self-directed manual speed	-0.160	-0.297	-0.017	-2.183	0.029
Fixed	Self-directed manual speed			-0.211	-0.306	-0.111	-4.089	0.000
Random	Self-directed manual speed			-0.211	-0.306	-0.111	-4.082	0.000
	Tremor	Bowler et al., 2016 CATSYS Tremor Intensity, dom. Air Mn	Tremor	-0.170	-0.306	-0.027	-2.322	0.020
	Tremor	Bowler et al., 2016 CATSYS Tremor Center frequency, dom. Air Mn	Tremor	-0.340	-0.461	-0.206	-4.790	0.000
	Tremor	Bowler et al., 2016 CATSYS Tremor Harmonic index, dom. Air Mn	Tremor	0.330	0.195	0.452	4.638	0.000
	Tremor	Bowler et al., 2016 CATSYS Tremor Intensity, non-dom. Air Mn	Tremor	-0.170	-0.306	-0.027	-2.322	0.020
	Tremor	Bowler et al., 2016 CATSYS Tremor Center frequency, non-dom. Air Mn	Tremor	-0.330	-0.452	-0.195	-4.638	0.000
	Tremor	Bowler et al., 2016 CATSYS Tremor Harmonic index, non-dom. Air Mn	Tremor	0.360	0.228	0.479	5.098	0.000
Fixed	Tremor			-0.053	-0.112	0.006	-1.770	0.077
Random	Tremor			-0.053	-0.307	0.208	-0.397	0.692
Fixed	Overall			-0.122	-0.155	-0.089	-7.086	0.000
Random	Overall			-0.153	-0.194	-0.111	-7.140	0.000

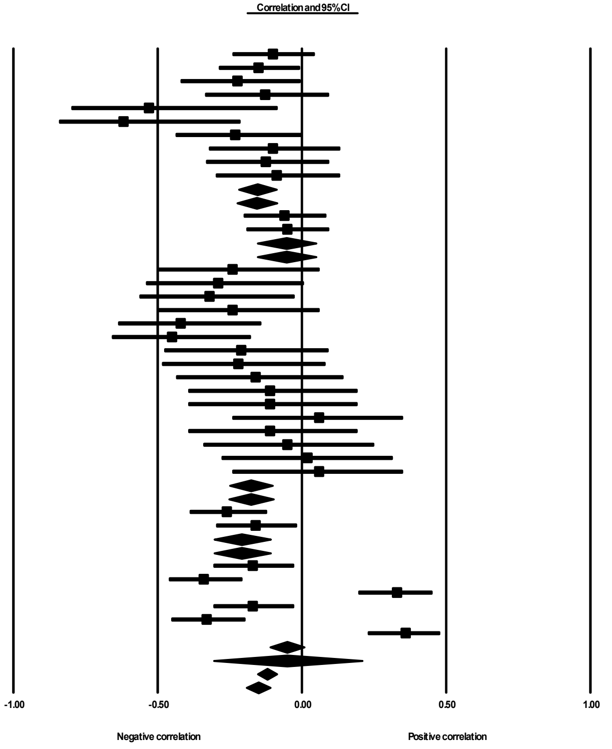

Online supplementary Figure S9. *Correlation between motor function and Mn exposure, as a function of the motor domain assessed.Note: a negative correlation indicates that the higher the Mn levels, the worse the motor function. A random effects model is used to combine studies within each subgroup. A fixed effect model is used to combine subgroups and yield the overall effect. The study-to-study variance (tau-squared) is NOT assumed to be the same for all subgroups - this value is computed within subgroups and NOT pooled across subgroups.*

Model	Group by Subgroup within study	Study name	Subgroup within study	Correlation	Lower limit	Upper limit	Z-Value	p-Value
	Air Mn	Bowler et al., 2016 Finger Tapping dom T-score. Air Mn	Air Mn	-0.260	-0.389	-0.121	-3.600	0.000
	Air Mn	Bowler et al., 2016 Finger Tapping non-dom T-score. Air Mn	Air Mn	-0.160	-0.297	-0.017	-2.183	0.029
	Air Mn	Bowler et al., 2016 Dynamometer dom T-score. Air Mn	Air Mn	-0.060	-0.202	0.085	-0.813	0.416
	Air Mn	Bowler et al., 2016 Dynamometer non-dom T-score. Air Mn	Air Mn	-0.050	-0.192	0.095	-0.677	0.498
	Air Mn	Bowler et al., 2016 Grooved Pegboard dom T-score. Air Mn	Air Mn	-0.100	-0.240	0.045	-1.357	0.175
	Air Mn	Bowler et al., 2016 Grooved Pegboard non-dom T-score. Air Mn	Air Mn	-0.150	-0.288	-0.006	-2.045	0.041
	Air Mn	Bowler et al., 2016 CATSYS Tremor Intensity, dom. Air Mn	Air Mn	-0.170	-0.306	-0.027	-2.322	0.020
	Air Mn	Bowler et al., 2016 CATSYS Tremor Center frequency, dom. Air Mn	Air Mn	-0.340	-0.461	-0.206	-4.790	0.000
	Air Mn	Bowler et al., 2016 CATSYS Tremor Harmonic index, dom. Air Mn	Air Mn	0.330	0.195	0.452	4.638	0.000
	Air Mn	Bowler et al., 2016 CATSYS Tremor Intensity, non-dom. Air Mn	Air Mn	-0.170	-0.306	-0.027	-2.322	0.020
	Air Mn	Bowler et al., 2016 CATSYS Tremor Center frequency, non-dom. Air Mn	Air Mn	-0.330	-0.452	-0.195	-4.638	0.000
	Air Mn	Bowler et al., 2016 CATSYS Tremor Harmonic index, non-dom. Air Mn	Air Mn	0.360	0.228	0.479	5.098	0.000
Fixed	Air Mn			-0.092	-0.133	-0.051	-4.333	0.000
Random	Air Mn			-0.092	-0.220	0.039	-1.378	0.168
	Axilary Hair Mn	Viana et al., 2014 Grooved Pegboard dom (seconds). Axilary Hair Mn	Axilary Hair Mn	-0.530	-0.799	-0.084	-2.286	0.022
	Axilary Hair Mn	Viana et al., 2014 Grooved Pegboard non-dom (seconds). Axilary Hair Mn	Axilary Hair Mn	-0.618	-0.842	-0.212	-2.795	0.005
Fixed	Axilary Hair Mn			-0.576	-0.767	-0.290	-3.593	0.000
Random	Axilary Hair Mn			-0.576	-0.767	-0.290	-3.593	0.000
	Blood Mn	Standridge et al., 2008 Postural balance testing (eyes open on the platform), Ln Sway area (cm2). Blood Mn	Blood Mn	-0.160	-0.436	0.144	-1.033	0.301
	Blood Mn	Standridge et al., 2008 Postural balance testing (eyes closed on the platform), Ln Sway area (cm2). Blood Mn	Blood Mn	-0.110	-0.394	0.193	-0.707	0.479
	Blood Mn	Standridge et al., 2008 Postural balance testing (eyes open on a 4 inch thick foam pad), Ln Sway area (cm2). Blood Mn	Blood Mn	-0.110	-0.394	0.193	-0.707	0.479
	Blood Mn	Standridge et al., 2008 Postural balance testing (eyes closed on a 4 inch thick foam pad), Ln Sway area (cm2). Blood Mn	Blood Mn	0.060	-0.241	0.351	0.385	0.700
	Blood Mn	Standridge et al., 2008 Postural balance testing (eyes open on the platform), Ln Sway length (cm). Blood Mn	Blood Mn	-0.110	-0.394	0.193	-0.707	0.479
	Blood Mn	Standridge et al., 2008 Postural balance testing (eyes closed on the platform), Ln Sway length (cm). Blood Mn	Blood Mn	-0.050	-0.342	0.251	-0.320	0.749
	Blood Mn	Standridge et al., 2008 Postural balance testing (eyes open on a 4 inch thick foam pad), Ln Sway lenght (cm). Blood Mn	Blood Mn	0.020	-0.279	0.315	0.128	0.898
	Blood Mn	Standridge et al., 2008 Postural balance testing (eyes closed on a 4 inch thick foam pad), Ln Sway lenght (cm). Blood Mn	Blood Mn	0.060	-0.241	0.351	0.385	0.700
Fixed	Blood Mn			-0.050	-0.157	0.058	-0.911	0.362
Random	Blood Mn			-0.050	-0.157	0.058	-0.911	0.362
	Hand Fingernails Mn	Viana et al., 2014 Grooved Pegboard dom (seconds). Hand Fingernails Mn	Hand Fingernails Mn	-0.231	-0.438	-0.001	-1.968	0.049
	Hand Fingernails Mn	Viana et al., 2014 Grooved Pegboard non-dom (seconds). Hand Fingernails Mn	Hand Fingernails Mn	-0.100	-0.323	0.133	-0.839	0.401
Fixed	Hand Fingernails Mn			-0.166	-0.322	-0.002	-1.985	0.047
Random	Hand Fingernails Mn			-0.166	-0.322	-0.002	-1.985	0.047
	Saliva Mn	Viana et al., 2014 Grooved Pegboard dom (seconds). Saliva Mn	Saliva Mn	-0.125	-0.333	0.095	-1.117	0.264
	Saliva Mn	Viana et al., 2014 Grooved Pegboard non-dom (seconds). Saliva Mn	Saliva Mn	-0.087	-0.298	0.133	-0.775	0.438
Fixed	Saliva Mn			-0.106	-0.257	0.049	-1.338	0.181
Random	Saliva Mn			-0.106	-0.257	0.049	-1.338	0.181
	Scalp Hair Mn	Standridge et al., 2008 Postural balance testing (eyes open on the platform), Ln Sway area (cm2). Hair Mn	Scalp Hair Mn	-0.240	-0.501	0.061	-1.567	0.117
	Scalp Hair Mn	Standridge et al., 2008 Postural balance testing (eyes closed on the platform), Ln Sway area (cm2). Hair Mn	Scalp Hair Mn	-0.290	-0.540	0.008	-1.912	0.056
	Scalp Hair Mn	Standridge et al., 2008 Postural balance testing (eyes open on a 4 inch thick foam pad), Ln Sway area (cm2). Hair Mn	Scalp Hair Mn	-0.320	-0.563	-0.026	-2.124	0.034
	Scalp Hair Mn	Standridge et al., 2008 Postural balance testing (eyes closed on a 4 inch thick foam pad), Ln Sway area (cm2). Hair Mn	Scalp Hair Mn	-0.240	-0.501	0.061	-1.567	0.117
	Scalp Hair Mn	Standridge et al., 2008 Postural balance testing (eyes open on the platform), Ln Sway length (cm). Hair Mn	Scalp Hair Mn	-0.420	-0.637	-0.141	-2.867	0.004
	Scalp Hair Mn	Standridge et al., 2008 Postural balance testing (eyes closed on the platform), Ln Sway length (cm). Hair Mn	Scalp Hair Mn	-0.450	-0.659	-0.177	-3.104	0.002
	Scalp Hair Mn	Standridge et al., 2008 Postural balance testing (eyes open on a 4 inch thick foam pad), Ln Sway lenght (cm). Hair Mn	Scalp Hair Mn	-0.210	-0.477	0.093	-1.365	0.172
	Scalp Hair Mn	Standridge et al., 2008 Postural balance testing (eyes closed on a 4 inch thick foam pad), Ln Sway lenght (cm). Hair Mn	Scalp Hair Mn	-0.220	-0.485	0.082	-1.432	0.152
	Scalp Hair Mn	Viana et al., 2014 Grooved Pegboard dom (seconds). Scalp Hair Mn	Scalp Hair Mn	-0.223	-0.421	-0.005	-2.003	0.045
	Scalp Hair Mn	Viana et al., 2014 Grooved Pegboard non-dom (seconds). Scalp Hair Mn	Scalp Hair Mn	-0.127	-0.336	0.094	-1.128	0.259
Fixed	Scalp Hair Mn			-0.262	-0.343	-0.177	-5.895	0.000
Random	Scalp Hair Mn			-0.262	-0.343	-0.177	-5.895	0.000
Fixed	Overall			-0.122	-0.155	-0.089	-7.086	0.000
Random	Overall			-0.165	-0.216	-0.113	-6.143	0.000

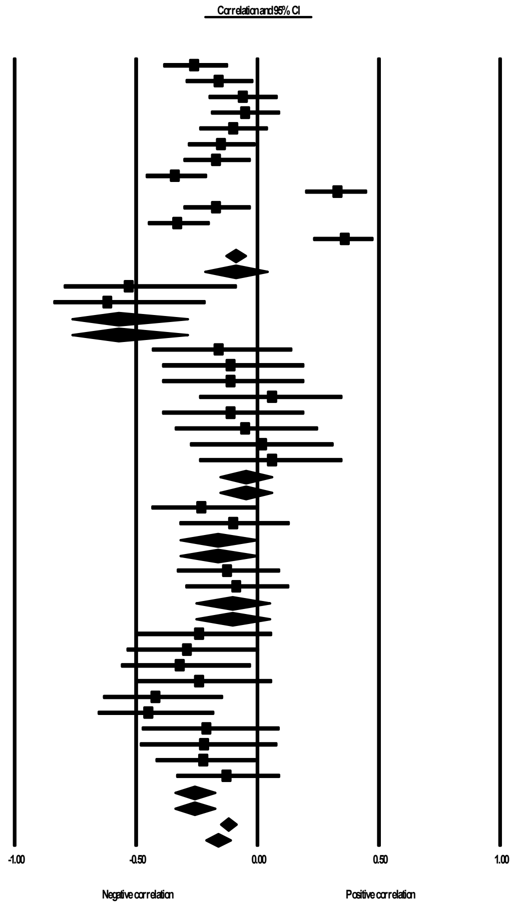

Online supplementary Figure S10. *Correlation between motor function and Mn exposure, as a function of the type of exposure to Mn evaluated.Note: a negative correlation indicates that the higher the Mn levels, the worse the motor function. A random effects model is used to combine studies within each subgroup. A fixed effect model is used to combine subgroups and yield the overall effect. The study-to-study variance (tau-squared) is NOT assumed to be the same for all subgroups - this value is computed within subgroups and NOT pooled across subgroups.*

Model	Study name	Statistics for each study						
		Std diff in means	Standard error	Variance	Lower limit	Upper limit	Z-Value	p-Value
	Bowler et al., 2012 Finger Tapping dom t-score	0.034	0.145	0.021	-0.251	0.318	0.232	0.817
	Bowler et al., 2012 Finger Tapping non-dom t-score	-0.019	0.145	0.021	-0.304	0.266	-0.130	0.897
	Bowler et al., 2012 Dynamometer dom t-score	0.239	0.146	0.021	-0.046	0.525	1.642	0.101
	Bowler et al., 2012 Dynamometer non-dom t-score	0.118	0.145	0.021	-0.167	0.403	0.814	0.415
	Bowler et al., 2012 Grooved Pegboard dom t score	-0.183	0.146	0.021	-0.468	0.103	-1.255	0.209
	Bowler et al., 2012 Grooved Pegboard non-dom t-score	-0.148	0.145	0.021	-0.433	0.138	-1.015	0.310
	Bowler et al., 2012 Unified Parkinson's Disease Rating Scale (UPDRS), Body bradykinesia	-0.196	0.146	0.021	-0.483	0.090	-1.345	0.179
	Bowler et al., 2012 UPDRS, Motor	-0.221	0.146	0.021	-0.508	0.065	-1.516	0.130
	Guarneros et al., 2013 Sniffin' sticks test, threshold	-0.517	0.262	0.069	-1.031	-0.002	-1.969	0.049
	Guarneros et al., 2013 Sniffin' sticks test, discrimination	-0.688	0.266	0.071	-1.208	-0.167	-2.588	0.010
	Guarneros et al., 2013 Sniffin' sticks test, identification	-0.688	0.266	0.071	-1.208	-0.167	-2.588	0.010
	Guarneros et al., 2013 Sniffin' sticks test, overall performance	-0.754	0.267	0.071	-1.277	-0.230	-2.820	0.005
	Kim et al., 2011 Computerized Adaptive Testing System (CATSYS) Postural Sway, (Sway 1) Mean sway (mm)	-0.228	0.146	0.021	-0.515	0.058	-1.562	0.118
	Kim et al., 2011 CATSYS Postural Sway, (Sway 1) Transversal sway (mm)	-0.053	0.146	0.021	-0.339	0.232	-0.366	0.714
	Kim et al., 2011 CATSYS Postural Sway, (Sway 1) Sagittal sway (mm)	-0.351	0.147	0.022	-0.639	-0.063	-2.392	0.017
	Kim et al., 2011 CATSYS Postural Sway, (Sway 1) Sway area (mm2)	-0.054	0.146	0.021	-0.339	0.232	-0.369	0.712
	Kim et al., 2011 CATSYS Postural Sway, (Sway 1) Sway intensity	-0.240	0.146	0.021	-0.527	0.046	-1.645	0.100
	Kim et al., 2011 CATSYS Postural Sway, (Sway 1) Sway velocity (mm/s)	-0.006	0.146	0.021	-0.291	0.280	-0.039	0.969
	Kim et al., 2011 CATSYS Postural Sway, (Sway 3) Mean sway (mm)	-0.299	0.146	0.021	-0.586	-0.012	-2.044	0.041
	Kim et al., 2011 CATSYS Postural Sway, (Sway 3) Transversal sway (mm)	-0.074	0.146	0.021	-0.360	0.212	-0.508	0.612
	Kim et al., 2011 CATSYS Postural Sway, (Sway 3) Sagittal sway (mm)	-0.418	0.147	0.022	-0.706	-0.129	-2.839	0.005
	Kim et al., 2011 CATSYS Postural Sway, (Sway 3) Sway area (mm2)	-0.151	0.146	0.021	-0.437	0.135	-1.037	0.300
	Kim et al., 2011 CATSYS Postural Sway, (Sway 3) Sway intensity	-0.093	0.146	0.021	-0.378	0.193	-0.636	0.525
	Kim et al., 2011 CATSYS Postural Sway, (Sway 3) Sway velocity (mm/s)	-0.138	0.146	0.021	-0.424	0.148	-0.948	0.343
	Lucchini et al., 2014 Finger Tapping dom	0.000	0.129	0.017	-0.253	0.253	0.000	1.000
	Lucchini et al., 2014 Finger Tapping non-dom	0.085	0.129	0.017	-0.168	0.338	0.659	0.510
	Lucchini et al., 2014 Sniffin' Sticks test, identification	-0.333	0.129	0.017	-0.586	-0.080	-2.584	0.010
	Lucchini et al., 2014 CATSYS Postural Sway, (Sway 1) Mean Sway (mm)	-0.092	0.132	0.017	-0.351	0.168	-0.693	0.488
	Lucchini et al., 2014 CATSYS Postural Sway, (Sway 1) Transversal sway (mm)	0.066	0.132	0.017	-0.194	0.325	0.495	0.620
	Lucchini et al., 2014 CATSYS Postural Sway, (Sway 1) Sagittal sway (mm)	-0.063	0.132	0.017	-0.323	0.196	-0.479	0.632
	Lucchini et al., 2014 CATSYS Postural Sway, (Sway 1) Sway area (mm2)	-0.069	0.132	0.017	-0.328	0.190	-0.521	0.602
	Lucchini et al., 2014 CATSYS Postural Sway, (Sway 1) Sway intensity	0.002	0.132	0.017	-0.257	0.261	0.017	0.986
	Lucchini et al., 2014 CATSYS Postural Sway, (Sway 1) Sway velocity (mm/s)	0.000	0.132	0.017	-0.259	0.259	0.000	1.000
	Lucchini et al., 2014 CATSYS Postural Sway, (Sway 2) Mean Sway (mm) assesment EC	0.039	0.132	0.017	-0.220	0.298	0.294	0.769
	Lucchini et al., 2014 CATSYS Postural Sway, (Sway 2) Transversal sway (mm)	0.053	0.132	0.017	-0.206	0.312	0.401	0.689
	Lucchini et al., 2014 CATSYS Postural Sway, (Sway 2) Sagittal sway (mm)	0.066	0.132	0.017	-0.194	0.325	0.495	0.620
	Lucchini et al., 2014 CATSYS Postural Sway, (Sway 2) Sway area (mm2)	-0.005	0.132	0.017	-0.264	0.254	-0.037	0.970
	Lucchini et al., 2014 CATSYS Postural Sway, (Sway 2) Sway intensity	0.018	0.132	0.017	-0.241	0.277	0.135	0.893
	Lucchini et al., 2014 CATSYS Postural Sway, (Sway 2) Sway velocity (mm/s)	0.065	0.132	0.017	-0.194	0.324	0.489	0.625
	Lucchini et al., 2014 CATSYS Tremor, Tremor Intensity Right Hand (m/s2)	0.062	0.132	0.017	-0.197	0.321	0.469	0.639
	Lucchini et al., 2014 CATSYS Tremor, Centre Frequency Right Hand (Hz)	-0.052	0.132	0.017	-0.311	0.207	-0.391	0.696
	Lucchini et al., 2014 CATSYS Tremor, Frequency dispersion Right Hand (Hz)	0.103	0.132	0.017	-0.156	0.363	0.782	0.434
	Lucchini et al., 2014 CATSYS Tremor Harmonic index Right Hand	0.000	0.132	0.017	-0.259	0.259	0.000	1.000
	Lucchini et al., 2014 CATSYS Tremor, Tremor Intensity Left Hand (m/s2)	0.000	0.132	0.017	-0.259	0.259	0.000	1.000
	Lucchini et al., 2014 CATSYS Tremor, Centre Frequency Left Hand (Hz)	0.103	0.132	0.017	-0.157	0.362	0.776	0.438
	Lucchini et al., 2014 CATSYS Tremor, Frequency dispersion Leftt Hand (Hz)	0.000	0.132	0.017	-0.259	0.259	0.000	1.000
	Lucchini et al., 2014 CATSYSTremor, Harmonic index Left Hand	0.000	0.132	0.017	-0.259	0.259	0.000	1.000
	Lucchini et al., 2014 Luria Nebraska, Task 1	-0.173	0.128	0.016	-0.424	0.078	-1.349	0.177
	Lucchini et al., 2014 Luria Nebraska, Task 2	-0.254	0.128	0.017	-0.506	-0.002	-1.978	0.048
	Lucchini et al., 2014 Luria Nebraska, Task 3	-0.079	0.128	0.016	-0.330	0.172	-0.616	0.538
	Lucchini et al., 2014 Luria Nebraska, Task 4	-0.480	0.130	0.017	-0.734	-0.226	-3.699	0.000
	Lucchini et al., 2014 Luria Nebraska, Task 5	-0.539	0.130	0.017	-0.795	-0.284	-4.142	0.000
	Lucchini et al., 2014 Luria Nebraska, sum score of the five subtests	-0.204	0.128	0.016	-0.456	0.047	-1.591	0.112
	Lucchini et al., 2014 Simple Visual Reaction Time, Mean (miliseconds)	0.000	0.129	0.017	-0.253	0.253	0.000	1.000
	Lucchini et al., 2014 Simple Visual Reaction Time, Std Dev	0.083	0.129	0.017	-0.170	0.336	0.641	0.522
	Mergler et al., 1999 Eurythmokinesimeter (EKM) Irregularity (<50 years)	-0.305	0.155	0.024	-0.609	-0.000	-1.963	0.050
	Mergler et al., 1999 EKM Irregularity (>50 years)	-0.404	0.205	0.042	-0.806	-0.001	-1.965	0.049
	Mergler et al., 1999 EKM Fitts´ constant (<50years)	-0.405	0.157	0.025	-0.712	-0.097	-2.580	0.010
	Mergler et al., 1999 EKM Fitts´constant (>50 years)	-0.538	0.208	0.043	-0.947	-0.130	-2.583	0.010
	Mergler et al., 1999 Purdue Pegboard (<50 years)	0.410	0.159	0.025	0.098	0.721	2.580	0.010
	Mergler et al., 1999 Purdue Pegboard (>50 years)	-0.538	0.208	0.043	-0.947	-0.130	-2.583	0.010
	Standridge et al., 2008 Postural balance testing (eyes open on the platform), Ln Sway area (cm2)	-1.078	0.323	0.104	-1.710	-0.445	-3.340	0.001
	Standridge et al., 2008 Postural balance testing (eyes closed on the platform), Ln Sway area (cm2)	-0.583	0.308	0.095	-1.186	0.021	-1.893	0.058
	Standridge et al., 2008 Postural balance testing (eyes open on a 4 inch thick foam pad), Ln Sway area (cm2)	-1.229	0.329	0.108	-1.873	-0.584	-3.737	0.000
	Standridge et al., 2008 Postural balance testing (eyes closed on a 4 inch thick foam pad), Ln Sway area (cm2)	-0.918	0.317	0.100	-1.540	-0.297	-2.897	0.004
	Standridge et al., 2008 Postural balance testing (eyes open on the platform), Ln Sway length (cm)	-0.466	0.306	0.093	-1.065	0.133	-1.524	0.128
	Standridge et al., 2008 Postural balance testing (eyes closed on the platform), Ln Sway length (cm)	-0.285	0.303	0.092	-0.879	0.309	-0.940	0.347
	Standridge et al., 2008 Postural balance testing (eyes open on a 4 inch thick foam pad), Ln Sway lenght (cm)	-0.677	0.310	0.096	-1.285	-0.070	-2.185	0.029
	Standridge et al ., 2008 Postural balance testing (eyes closed on a 4 inch thick foam pad), Ln Sway lenght (cm)	-0.608	0.308	0.095	-1.213	-0.004	-1.973	0.049
	Viana et al., 2014 Grooved Pegboard dom (seconds)	-0.128	0.219	0.048	-0.557	0.301	-0.585	0.559
	Viana et al., 2014 Grooved Pegboard non-dom (seconds)	0.191	0.219	0.048	-0.238	0.621	0.873	0.383
Fixed		-0.114	0.018	0.000	-0.149	-0.079	-6.348	0.000
Random		-0.140	0.028	0.001	-0.194	-0.086	-5.073	0.000

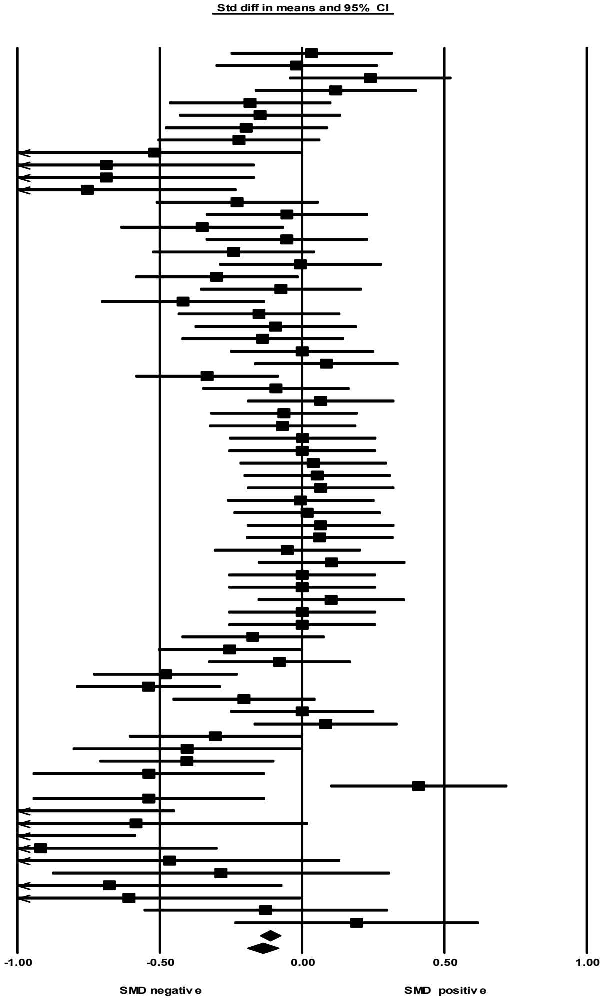

Online supplementary Figure S11. *Motor function. Standardized mean differences (SMDs) between groups, prioritizing the use of medians versus means (when both medians and means were reported). All tests and all exposures to Mn. Note: a negative SMD indicates that the group with higher Mn levels had worse motor function on average.*

Model	Group by / Subgroup within study	Study name	Subgroup within study	Std diff in means	Standard error	Variance	Lower limit	Upper limit	Z-Value	p-Value
	CATSYS	Kim et al., 2011 Computerized Adaptive Testing System (CATSYS) Postural Sway, (Sway 1) Mean sway (mm)	CATSYS	-0.228	0.146	0.021	-0.515	0.058	-1.562	0.118
	CATSYS	Kim et al., 2011 CATSYS Postural Sway, (Sway 1) Transversal sway (mm)	CATSYS	-0.053	0.146	0.021	-0.339	0.232	-0.366	0.714
	CATSYS	Kim et al., 2011 CATSYS Postural Sway, (Sway 1) Sagittal sway (mm)	CATSYS	-0.351	0.147	0.022	-0.639	-0.063	-2.392	0.017
	CATSYS	Kim et al., 2011 CATSYS Postural Sway, (Sway 1) Sway area (mm2)	CATSYS	-0.054	0.146	0.021	-0.339	0.232	-0.369	0.712
	CATSYS	Kim et al., 2011 CATSYS Postural Sway, (Sway 1) Sway intensity	CATSYS	-0.240	0.146	0.021	-0.527	0.046	-1.645	0.100
	CATSYS	Kim et al., 2011 CATSYS Postural Sway, (Sway 1) Sway velocity (mm/s)	CATSYS	-0.006	0.146	0.021	-0.291	0.280	-0.039	0.969
	CATSYS	Kim et al., 2011 CATSYS Postural Sway, (Sway 3) Mean sway (mm)	CATSYS	-0.299	0.146	0.021	-0.586	-0.012	-2.044	0.041
	CATSYS	Kim et al., 2011 CATSYS Postural Sway, (Sway 3) Transversal sway (mm)	CATSYS	-0.074	0.146	0.021	-0.360	0.212	-0.508	0.612
	CATSYS	Kim et al., 2011 CATSYS Postural Sway, (Sway 3) Sagittal sway (mm)	CATSYS	-0.418	0.147	0.022	-0.706	-0.129	-2.839	0.005
	CATSYS	Kim et al., 2011 CATSYS Postural Sway, (Sway 3) Sway area (mm2)	CATSYS	-0.151	0.146	0.021	-0.437	0.135	-1.037	0.300
	CATSYS	Kim et al., 2011 CATSYS Postural Sway, (Sway 3) Sway intensity	CATSYS	-0.093	0.146	0.021	-0.378	0.193	-0.636	0.525
	CATSYS	Kim et al., 2011 CATSYS Postural Sway, (Sway 3) Sway velocity (mm/s)	CATSYS	-0.138	0.146	0.021	-0.424	0.148	-0.948	0.343
	CATSYS	Lucchini et al., 2014 CATSYS Postural Sway, (Sway 1) Mean Sway (mm)	CATSYS	0.028	0.132	0.017	-0.232	0.287	0.208	0.835
	CATSYS	Lucchini et al., 2014 CATSYS Postural Sway, (Sway 1) Transversal sway (mm)	CATSYS	-0.020	0.132	0.017	-0.279	0.240	-0.148	0.882
	CATSYS	Lucchini et al., 2014 CATSYS Postural Sway, (Sway 1) Sagittal sway (mm)	CATSYS	0.083	0.132	0.017	-0.177	0.342	0.624	0.533
	CATSYS	Lucchini et al., 2014 CATSYS Postural Sway, (Sway 1) Sway area (mm2)	CATSYS	0.022	0.132	0.017	-0.237	0.281	0.169	0.866
	CATSYS	Lucchini et al., 2014 CATSYS Postural Sway, (Sway 1) Sway intensity	CATSYS	-0.084	0.132	0.017	-0.344	0.175	-0.637	0.524
	CATSYS	Lucchini et al., 2014 CATSYS Postural Sway, (Sway 1) Sway velocity (mm/s)	CATSYS	-0.007	0.132	0.017	-0.266	0.252	-0.051	0.960
	CATSYS	Lucchini et al., 2014 CATSYS Postural Sway, (Sway 2) Mean Sway (mm) assesment EC	CATSYS	0.065	0.132	0.017	-0.194	0.324	0.490	0.624
	CATSYS	Lucchini et al., 2014 CATSYS Postural Sway, (Sway 2) Transversal sway (mm)	CATSYS	0.053	0.132	0.017	-0.206	0.312	0.401	0.689
	CATSYS	Lucchini et al., 2014 CATSYS Postural Sway, (Sway 2) Sagittal sway (mm)	CATSYS	0.079	0.132	0.017	-0.181	0.338	0.594	0.552
	CATSYS	Lucchini et al., 2014 CATSYS Postural Sway, (Sway 2) Sway area (mm2)	CATSYS	0.035	0.132	0.017	-0.225	0.294	0.261	0.794
	CATSYS	Lucchini et al., 2014 CATSYS Postural Sway, (Sway 2) Sway intensity	CATSYS	0.071	0.132	0.017	-0.188	0.330	0.537	0.591
	CATSYS	Lucchini et al., 2014 CATSYS Postural Sway, (Sway 2) Sway velocity (mm/s)	CATSYS	0.080	0.132	0.017	-0.179	0.339	0.603	0.546
	CATSYS	Lucchini et al., 2014 CATSYS Tremor, Tremor Intensity Right Hand (m/s2)	CATSYS	0.149	0.132	0.018	-0.111	0.408	1.123	0.261
	CATSYS	Lucchini et al., 2014 CATSYS Tremor, Centre Frequency Right Hand (Hz)	CATSYS	-0.031	0.132	0.017	-0.290	0.228	-0.234	0.815
	CATSYS	Lucchini et al., 2014 CATSYS Tremor, Frequency dispersion Right Hand (Hz)	CATSYS	0.083	0.132	0.017	-0.176	0.342	0.626	0.531
	CATSYS	Lucchini et al., 2014 CATSYS Tremor Harmonic index Right Hand	CATSYS	-0.068	0.132	0.017	-0.327	0.191	-0.516	0.606
	CATSYS	Lucchini et al., 2014 CATSYS Tremor, Tremor Intensity Left Hand (m/s2)	CATSYS	0.119	0.132	0.018	-0.141	0.378	0.896	0.370
	CATSYS	Lucchini et al., 2014 CATSYS Tremor, Centre Frequency Left Hand (Hz)	CATSYS	0.098	0.132	0.017	-0.162	0.357	0.737	0.461
	CATSYS	Lucchini et al., 2014 CATSYS Tremor, Frequency dispersion Leftt Hand (Hz)	CATSYS	0.106	0.132	0.018	-0.153	0.365	0.801	0.423
	CATSYS	Lucchini et al., 2014 CATSYSTremor, Harmonic index Left Hand	CATSYS	-0.087	0.132	0.017	-0.346	0.172	-0.658	0.511
Fixed	CATSYS			-0.032	0.024	0.001	-0.079	0.016	-1.313	0.189
Random	CATSYS			-0.032	0.024	0.001	-0.079	0.016	-1.312	0.189
	Dynamometer	Bowler et al., 2012 Dynamometer dom t-score	Dynamometer	0.239	0.146	0.021	-0.046	0.525	1.642	0.101
	Dynamometer	Bowler et al., 2012 Dynamometer non-dom t-score	Dynamometer	0.118	0.145	0.021	-0.167	0.403	0.814	0.415
Fixed	Dynamometer			0.179	0.103	0.011	-0.023	0.381	1.736	0.083
Random	Dynamometer			0.179	0.103	0.011	-0.023	0.381	1.736	0.083
	EKM	Mergler et al., 1999 Eurythmokinesimeter (EKM) Irregularity (<50 years)	EKM	-0.305	0.155	0.024	-0.609	-0.000	-1.963	0.050
	EKM	Mergler et al., 1999 EKM Irregularity (>50 years)	EKM	-0.404	0.205	0.042	-0.806	-0.001	-1.965	0.049
	EKM	Mergler et al., 1999 EKM Fitts´ constant (<50years)	EKM	-0.405	0.157	0.025	-0.712	-0.097	-2.580	0.010
	EKM	Mergler et al., 1999 EKM Fitts´constant (>50 years)	EKM	-0.538	0.208	0.043	-0.947	-0.130	-2.583	0.010
Fixed	EKM			-0.396	0.088	0.008	-0.569	-0.223	-4.497	0.000
Random	EKM			-0.396	0.088	0.008	-0.569	-0.223	-4.497	0.000
	Finger Tapping	Bowler et al., 2012 Finger Tapping dom t-score	Finger Tapping	0.034	0.145	0.021	-0.251	0.318	0.232	0.817
	Finger Tapping	Bowler et al., 2012 Finger Tapping non-dom t-score	Finger Tapping	-0.019	0.145	0.021	-0.304	0.266	-0.130	0.897
	Finger Tapping	Lucchini et al., 2014 Finger Tapping dom	Finger Tapping	-0.171	0.129	0.017	-0.425	0.082	-1.323	0.186
	Finger Tapping	Lucchini et al., 2014 Finger Tapping non-dom	Finger Tapping	-0.112	0.129	0.017	-0.366	0.141	-0.870	0.385
Fixed	Finger Tapping			-0.076	0.068	0.005	-0.210	0.058	-1.110	0.267
Random	Finger Tapping			-0.076	0.068	0.005	-0.210	0.058	-1.110	0.267
	Grooved Pegboard	Bowler et al., 2012 Grooved Pegboard dom tscore	Grooved Pegboard	-0.183	0.146	0.021	-0.468	0.103	-1.255	0.209
	Grooved Pegboard	Bowler et al., 2012 Grooved Pegboard non-dom t-score	Grooved Pegboard	-0.148	0.145	0.021	-0.433	0.138	-1.015	0.310
	Grooved Pegboard	Viana et al., 2014 Grooved Pegboard dom (seconds)	Grooved Pegboard	-0.128	0.219	0.048	-0.557	0.301	-0.585	0.559
	Grooved Pegboard	Viana et al., 2014 Grooved Pegboard non-dom (seconds)	Grooved Pegboard	0.191	0.219	0.048	-0.238	0.621	0.873	0.383
Fixed	Grooved Pegboard			-0.105	0.086	0.007	-0.273	0.063	-1.225	0.221
Random	Grooved Pegboard			-0.105	0.086	0.007	-0.273	0.063	-1.225	0.221
	Luria Nebraska	Lucchini et al., 2014 Luria Nebraska, Task 1	Luria Nebraska	-0.296	0.129	0.017	-0.548	-0.044	-2.299	0.022
	Luria Nebraska	Lucchini et al., 2014 Luria Nebraska, Task 2	Luria Nebraska	-0.288	0.129	0.017	-0.540	-0.036	-2.240	0.025
	Luria Nebraska	Lucchini et al., 2014 Luria Nebraska, Task 3	Luria Nebraska	-0.027	0.128	0.016	-0.278	0.224	-0.209	0.834
	Luria Nebraska	Lucchini et al., 2014 Luria Nebraska, Task 4	Luria Nebraska	-0.278	0.129	0.017	-0.530	-0.026	-2.165	0.030
	Luria Nebraska	Lucchini et al., 2014 Luria Nebraska, Task 5	Luria Nebraska	-0.259	0.129	0.017	-0.511	-0.007	-2.014	0.044
	Luria Nebraska	Lucchini et al., 2014 Luria Nebraska, sum score of the five subtests	Luria Nebraska	-0.315	0.129	0.017	-0.567	-0.063	-2.447	0.014
Fixed	Luria Nebraska			-0.243	0.052	0.003	-0.346	-0.141	-4.640	0.000
Random	Luria Nebraska			-0.243	0.052	0.003	-0.346	-0.141	-4.640	0.000
	Postural balance testing	Standridge et al., 2008 Postural balance testing (eyes open on the platform), Ln Sway area (cm2)	Postural balance testing	-1.078	0.323	0.104	-1.710	-0.445	-3.340	0.001
	Postural balance testing	Standridge et al., 2008 Postural balance testing (eyes closed on the platform), Ln Sway area (cm2)	Postural balance testing	-0.583	0.308	0.095	-1.186	0.021	-1.893	0.058
	Postural balance testing	Standridge et al., 2008 Postural balance testing (eyes open on a 4 inch thick foam pad), Ln Sway area (cm2)	Postural balance testing	-1.229	0.329	0.108	-1.873	-0.584	-3.737	0.000
	Postural balance testing	Standridge et al., 2008 Postural balance testing (eyes closed on a 4 inch thick foam pad), Ln Sway area (cm2)	Postural balance testing	-0.918	0.317	0.100	-1.540	-0.297	-2.897	0.004
	Postural balance testing	Standridge et al., 2008 Postural balance testing (eyes open on the platform), Ln Sway length (cm)	Postural balance testing	-0.466	0.306	0.093	-1.065	0.133	-1.524	0.128
	Postural balance testing	Standridge et al., 2008 Postural balance testing (eyes closed on the platform), Ln Sway length (cm)	Postural balance testing	-0.285	0.303	0.092	-0.879	0.309	-0.940	0.347
	Postural balance testing	Standridge et al., 2008 Postural balance testing (eyes open on a 4 inch thick foam pad), Ln Sway lenght (cm)	Postural balance testing	-0.677	0.310	0.096	-1.285	-0.070	-2.185	0.029
	Postural balance testing	Standridge et al ., 2008 Postural balance testing (eyes closed on a 4 inch thick foam pad), Ln Sway lenght (cm)	Postural balance testing	-0.608	0.308	0.095	-1.213	-0.004	-1.973	0.049
Fixed	Postural balance testing			-0.715	0.111	0.012	-0.932	-0.498	-6.470	0.000
Random	Postural balance testing			-0.715	0.111	0.012	-0.934	-0.497	-6.420	0.000
	Purdue Pegboard	Mergler et al., 1999 Purdue Pegboard (<50 years)	Purdue Pegboard	0.410	0.159	0.025	0.098	0.721	2.580	0.010
	Purdue Pegboard	Mergler et al., 1999 Purdue Pegboard (>50 years)	Purdue Pegboard	-0.538	0.208	0.043	-0.947	-0.130	-2.583	0.010
Fixed	Purdue Pegboard			0.061	0.126	0.016	-0.186	0.309	0.487	0.627
Random	Purdue Pegboard			-0.055	0.474	0.224	-0.983	0.874	-0.115	0.908
	Simple Visual Reaction Time	Lucchini et al., 2014 Simple Visual Reaction Time, Mean (miliseconds)	Simple Visual Reaction Time	-0.101	0.129	0.017	-0.355	0.152	-0.784	0.433
	Simple Visual Reaction Time	Lucchini et al., 2014 Simple Visual Reaction Time, Std Dev	Simple Visual Reaction Time	-0.180	0.129	0.017	-0.433	0.074	-1.390	0.165
Fixed	Simple Visual Reaction Time			-0.141	0.091	0.008	-0.320	0.039	-1.537	0.124
Random	Simple Visual Reaction Time			-0.141	0.091	0.008	-0.320	0.039	-1.537	0.124
	Sniffin' sticks test	Guarneros et al., 2013 Sniffin' sticks test, threshold	Sniffin' sticks test	-0.517	0.262	0.069	-1.031	-0.002	-1.969	0.049
	Sniffin' sticks test	Guarneros et al., 2013 Sniffin' sticks test, discrimination	Sniffin' sticks test	-0.688	0.266	0.071	-1.208	-0.167	-2.588	0.010
	Sniffin' sticks test	Guarneros et al., 2013 Sniffin' sticks test, identification	Sniffin' sticks test	-0.688	0.266	0.071	-1.208	-0.167	-2.588	0.010
	Sniffin' sticks test	Guarneros et al., 2013 Sniffin' sticks test, overall performance	Sniffin' sticks test	-0.754	0.267	0.071	-1.277	-0.230	-2.820	0.005
	Sniffin' sticks test	Lucchini et al., 2014 Sniffin' Sticks test, identification	Sniffin' sticks test	-0.233	0.129	0.017	-0.485	0.019	-1.815	0.070
Fixed	Sniffin' sticks test			-0.440	0.092	0.009	-0.621	-0.259	-4.768	0.000
Random	Sniffin' sticks test			-0.501	0.121	0.015	-0.738	-0.265	-4.154	0.000
	UPDRS	Bowler et al., 2012 Unified Parkinson's Disease Rating Scale (UPDRS), Body bradykinesia	UPDRS	-0.196	0.146	0.021	-0.483	0.090	-1.345	0.179
	UPDRS	Bowler et al., 2012 UPDRS, Motor	UPDRS	-0.221	0.146	0.021	-0.508	0.065	-1.516	0.130
Fixed	UPDRS			-0.209	0.103	0.011	-0.411	-0.007	-2.023	0.043
Random	UPDRS			-0.209	0.103	0.011	-0.411	-0.007	-2.023	0.043
Fixed	Overall			-0.112	0.018	0.000	-0.147	-0.077	-6.270	0.000
Random	Overall			-0.112	0.018	0.000	-0.147	-0.076	-6.128	0.000

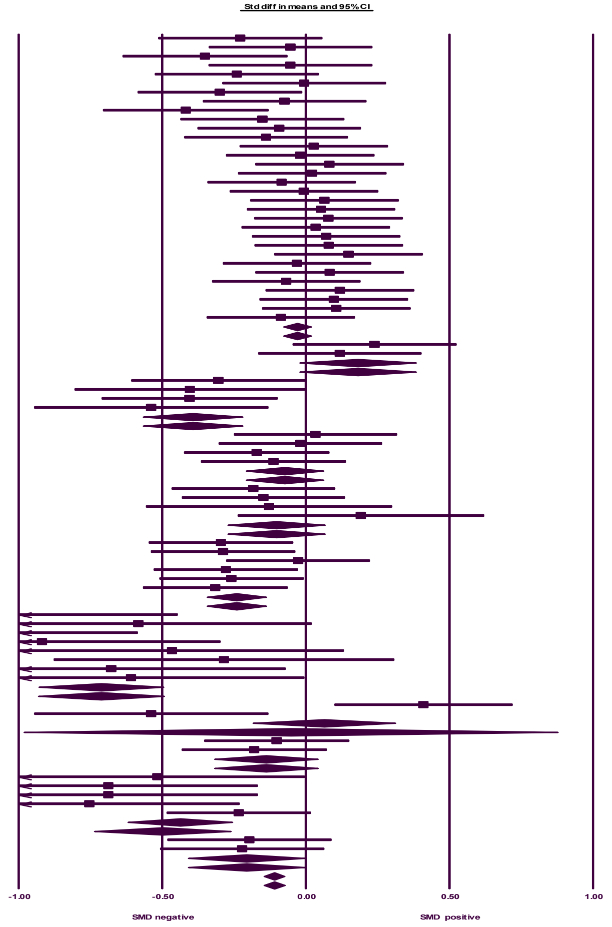

Online supplementary Figure S12. *Motor function. Standardized mean differences (SMDs) between groups, as a function of the motor test used. Note: a negative SMD indicates that the group with higher Mn levels had worse motor function on average. A random effects model is used to combine studies within each subgroup. A fixed effect model is used to combine subgroups and yield the overall effect. The study-to-study variance (tau-squared) is NOT assumed to be the same for all subgroups - this value is computed within subgroups and NOT pooled across subgroups.*

Model	Group by Subgroup within study	Study name	Subgroup within study	Statistics for each study						
				Std diff in means	Standard error	Variance	Lower limit	Upper limit	Z-Value	p-Value
	Bradykinesia	Bowler et al., 2012 Unified Parkinson's Disease Rating Scale (UPDRS), Body bradykinesia	Bradykinesia	-0.196	0.146	0.021	-0.483	0.090	-1.345	0.179
Fixed	Bradykinesia			-0.196	0.146	0.021	-0.483	0.090	-1.345	0.179
Random	Bradykinesia			-0.196	0.146	0.021	-0.483	0.090	-1.345	0.179
	Eye-hand coordination	Mergler et al., 1999 Eurythmokinesimeter (EKM) Irregularity (<50 years)	Eye-hand coordination	-0.305	0.155	0.024	-0.609	-0.000	-1.963	0.050
	Eye-hand coordination	Mergler et al., 1999 EKM Irregularity (>50 years)	Eye-hand coordination	-0.404	0.205	0.042	-0.806	-0.001	-1.965	0.049
	Eye-hand coordination	Mergler et al., 1999 EKM Fitts´ constant (<50years)	Eye-hand coordination	-0.405	0.157	0.025	-0.712	-0.097	-2.580	0.010
	Eye-hand coordination	Mergler et al., 1999 EKM Fitts´constant (>50 years)	Eye-hand coordination	-0.538	0.208	0.043	-0.947	-0.130	-2.583	0.010
Fixed	Eye-hand coordination			-0.396	0.088	0.008	-0.569	-0.223	-4.497	0.000
Random	Eye-hand coordination			-0.396	0.088	0.008	-0.569	-0.223	-4.497	0.000
	Eye-hand coordination and motor speed	Bowler et al., 2012 Grooved Pegboard dom t score	Eye-hand coordination and motor speed	-0.183	0.146	0.021	-0.468	0.103	-1.255	0.209
	Eye-hand coordination and motor speed	Bowler et al., 2012 Grooved Pegboard non-dom t-score	Eye-hand coordination and motor speed	-0.148	0.145	0.021	-0.433	0.138	-1.015	0.310
	Eye-hand coordination and motor speed	Viana et al., 2014 Grooved Pegboard dom (seconds)	Eye-hand coordination and motor speed	-0.128	0.219	0.048	-0.557	0.301	-0.585	0.559
	Eye-hand coordination and motor speed	Viana et al., 2014 Grooved Pegboard non-dom (seconds)	Eye-hand coordination and motor speed	0.191	0.219	0.048	-0.238	0.621	0.873	0.383
Fixed	Eye-hand coordination and motor speed			-0.105	0.086	0.007	-0.273	0.063	-1.225	0.221
Random	Eye-hand coordination and motor speed			-0.105	0.086	0.007	-0.273	0.063	-1.225	0.221
	Finger and hand dexterity	Mergler et al., 1999 Purdue Pegboard (<50 years)	Finger and hand dexterity	0.410	0.159	0.025	0.098	0.721	2.580	0.010
	Finger and hand dexterity	Mergler et al., 1999 Purdue Pegboard (>50 years)	Finger and hand dexterity	-0.538	0.208	0.043	-0.947	-0.130	-2.583	0.010
Fixed	Finger and hand dexterity			0.061	0.126	0.016	-0.186	0.309	0.487	0.627
Random	Finger and hand dexterity			-0.055	0.474	0.224	-0.983	0.874	-0.115	0.908
	Hand strength	Bowler et al., 2012 Dynamometer dom t-score	Hand strength	0.239	0.146	0.021	-0.046	0.525	1.642	0.101
	Hand strength	Bowler et al., 2012 Dynamometer non-dom t-score	Hand strength	0.118	0.145	0.021	-0.167	0.403	0.814	0.415
Fixed	Hand strength			0.179	0.103	0.011	-0.023	0.381	1.736	0.083
Random	Hand strength			0.179	0.103	0.011	-0.023	0.381	1.736	0.083
	Motor coordination	Lucchini et al., 2014 Luria Nebraska, Task 1	Motor coordination	-0.296	0.129	0.017	-0.548	-0.044	-2.299	0.022
	Motor coordination	Lucchini et al., 2014 Luria Nebraska, Task 2	Motor coordination	-0.288	0.129	0.017	-0.540	-0.036	-2.240	0.025
	Motor coordination	Lucchini et al., 2014 Luria Nebraska, Task 3	Motor coordination	-0.027	0.128	0.016	-0.278	0.224	-0.209	0.834
	Motor coordination	Lucchini et al., 2014 Luria Nebraska, Task 4	Motor coordination	-0.278	0.129	0.017	-0.530	-0.026	-2.165	0.030
	Motor coordination	Lucchini et al., 2014 Luria Nebraska, Task 5	Motor coordination	-0.259	0.129	0.017	-0.511	-0.007	-2.014	0.044
	Motor coordination	Lucchini et al., 2014 Luria Nebraska, sum score of the five subtests	Motor coordination	-0.315	0.129	0.017	-0.567	-0.063	-2.447	0.014
Fixed	Motor coordination			-0.243	0.052	0.003	-0.346	-0.141	-4.640	0.000
Random	Motor coordination			-0.243	0.052	0.003	-0.346	-0.141	-4.640	0.000
	Motor evaluation	Bowler et al., 2012 UPDRS, Motor	Motor evaluation	-0.221	0.146	0.021	-0.508	0.065	-1.516	0.130
Fixed	Motor evaluation			-0.221	0.146	0.021	-0.508	0.065	-1.516	0.130
Random	Motor evaluation			-0.221	0.146	0.021	-0.508	0.065	-1.516	0.130
	Olfactory performance	Guarneros et al., 2013 Sniffin' sticks test, threshold	Olfactory performance	-0.517	0.262	0.069	-1.031	-0.002	-1.969	0.049
	Olfactory performance	Guarneros et al., 2013 Sniffin' sticks test, discrimination	Olfactory performance	-0.688	0.266	0.071	-1.208	-0.167	-2.588	0.010
	Olfactory performance	Guarneros et al., 2013 Sniffin' sticks test, identification	Olfactory performance	-0.688	0.266	0.071	-1.208	-0.167	-2.588	0.010
	Olfactory performance	Guarneros et al., 2013 Sniffin' sticks test, overall performance	Olfactory performance	-0.754	0.267	0.071	-1.277	-0.230	-2.820	0.005
	Olfactory performance	Lucchini et al., 2014 Sniffin' Sticks test, identification	Olfactory performance	-0.233	0.129	0.017	-0.485	0.019	-1.815	0.070
Fixed	Olfactory performance			-0.440	0.092	0.009	-0.621	-0.259	-4.768	0.000
Random	Olfactory performance			-0.501	0.121	0.015	-0.738	-0.265	-4.154	0.000
	Postural balance	Standridge et al., 2008 Postural balance testing (eyes open on the platform), Ln Sway area (cm2)	Postural balance	-1.078	0.323	0.104	-1.710	-0.445	-3.340	0.001
	Postural balance	Standridge et al., 2008 Postural balance testing (eyes closed on the platform), Ln Sway area (cm2)	Postural balance	-0.583	0.308	0.095	-1.186	0.021	-1.893	0.058
	Postural balance	Standridge et al., 2008 Postural balance testing (eyes open on a 4 inch thick foam pad), Ln Sway area (cm2)	Postural balance	-1.229	0.329	0.108	-1.873	-0.584	-3.737	0.000
	Postural balance	Standridge et al., 2008 Postural balance testing (eyes closed on a 4 inch thick foam pad), Ln Sway area (cm2)	Postural balance	-0.918	0.317	0.100	-1.540	-0.297	-2.897	0.004
	Postural balance	Standridge et al., 2008 Postural balance testing (eyes open on the platform), Ln Sway length (cm)	Postural balance	-0.466	0.306	0.093	-1.065	0.133	-1.524	0.128
	Postural balance	Standridge et al., 2008 Postural balance testing (eyes closed on the platform), Ln Sway length (cm)	Postural balance	-0.285	0.303	0.092	-0.879	0.309	-0.940	0.347
	Postural balance	Standridge et al., 2008 Postural balance testing (eyes open on a 4 inch thick foam pad), Ln Sway lenght (cm)	Postural balance	-0.677	0.310	0.096	-1.285	-0.070	-2.185	0.029
	Postural balance	Standridge et al ., 2008 Postural balance testing (eyes closed on a 4 inch thick foam pad), Ln Sway lenght (cm)	Postural balance	-0.608	0.308	0.095	-1.213	-0.004	-1.973	0.049
Fixed	Postural balance			-0.715	0.111	0.012	-0.932	-0.498	-6.470	0.000
Random	Postural balance			-0.715	0.111	0.012	-0.934	-0.497	-6.420	0.000
	Postural sway	Kim et al., 2011 Computerized Adaptive Testing System (CATSYS) Postural Sway, (Sway 1) Mean sway (mm)	Postural sway	-0.228	0.146	0.021	-0.515	0.058	-1.562	0.118
	Postural sway	Kim et al., 2011 CATSYS Postural Sway, (Sway 1) Transversal sway (mm)	Postural sway	-0.053	0.146	0.021	-0.339	0.232	-0.366	0.714
	Postural sway	Kim et al., 2011 CATSYS Postural Sway, (Sway 1) Sagittal sway (mm)	Postural sway	-0.351	0.147	0.022	-0.639	-0.063	-2.392	0.017
	Postural sway	Kim et al., 2011 CATSYS Postural Sway, (Sway 1) Sway area (mm2)	Postural sway	-0.054	0.146	0.021	-0.339	0.232	-0.369	0.712
	Postural sway	Kim et al., 2011 CATSYS Postural Sway, (Sway 1) Sway intensity	Postural sway	-0.240	0.146	0.021	-0.527	0.046	-1.645	0.100
	Postural sway	Kim et al., 2011 CATSYS Postural Sway, (Sway 1) Sway velocity (mm/s)	Postural sway	-0.006	0.146	0.021	-0.291	0.280	-0.039	0.969
	Postural sway	Kim et al., 2011 CATSYS Postural Sway, (Sway 3) Mean sway (mm)	Postural sway	-0.299	0.146	0.021	-0.586	-0.012	-2.044	0.041
	Postural sway	Kim et al., 2011 CATSYS Postural Sway, (Sway 3) Transversal sway (mm)	Postural sway	-0.074	0.146	0.021	-0.360	0.212	-0.508	0.612
	Postural sway	Kim et al., 2011 CATSYS Postural Sway, (Sway 3) Sagittal sway (mm)	Postural sway	-0.418	0.147	0.022	-0.706	-0.129	-2.839	0.005
	Postural sway	Kim et al., 2011 CATSYS Postural Sway, (Sway 3) Sway area (mm2)	Postural sway	-0.151	0.146	0.021	-0.437	0.135	-1.037	0.300
	Postural sway	Kim et al., 2011 CATSYS Postural Sway, (Sway 3) Sway intensity	Postural sway	-0.093	0.146	0.021	-0.378	0.193	-0.636	0.525
	Postural sway	Kim et al., 2011 CATSYS Postural Sway, (Sway 3) Sway velocity (mm/s)	Postural sway	-0.138	0.146	0.021	-0.424	0.148	-0.948	0.343
	Postural sway	Lucchini et al., 2014 CATSYS Postural Sway, (Sway 1) Mean Sway (mm)	Postural sway	0.028	0.132	0.017	-0.232	0.287	0.208	0.835
	Postural sway	Lucchini et al., 2014 CATSYS Postural Sway, (Sway 1) Transversal sway (mm)	Postural sway	-0.020	0.132	0.017	-0.279	0.240	-0.148	0.882
	Postural sway	Lucchini et al., 2014 CATSYS Postural Sway, (Sway 1) Sagittal sway (mm)	Postural sway	0.083	0.132	0.017	-0.177	0.342	0.624	0.533
	Postural sway	Lucchini et al., 2014 CATSYS Postural Sway, (Sway 1) Sway area (mm2)	Postural sway	0.022	0.132	0.017	-0.237	0.281	0.169	0.866
	Postural sway	Lucchini et al., 2014 CATSYS Postural Sway, (Sway 1) Sway intensity	Postural sway	-0.084	0.132	0.017	-0.344	0.175	-0.637	0.524
	Postural sway	Lucchini et al., 2014 CATSYS Postural Sway, (Sway 1) Sway velocity (mm/s)	Postural sway	-0.007	0.132	0.017	-0.266	0.252	-0.051	0.960
	Postural sway	Lucchini et al., 2014 CATSYS Postural Sway, (Sway 2) Mean Sway (mm) assesment EC	Postural sway	0.065	0.132	0.017	-0.194	0.324	0.490	0.624
	Postural sway	Lucchini et al., 2014 CATSYS Postural Sway, (Sway 2) Transversal sway (mm)	Postural sway	0.053	0.132	0.017	-0.206	0.312	0.401	0.689
	Postural sway	Lucchini et al., 2014 CATSYS Postural Sway, (Sway 2) Sagittal sway (mm)	Postural sway	0.079	0.132	0.017	-0.181	0.338	0.594	0.552
	Postural sway	Lucchini et al., 2014 CATSYS Postural Sway, (Sway 2) Sway area (mm2)	Postural sway	0.035	0.132	0.017	-0.225	0.294	0.261	0.794
	Postural sway	Lucchini et al., 2014 CATSYS Postural Sway, (Sway 2) Sway intensity	Postural sway	0.071	0.132	0.017	-0.188	0.330	0.537	0.591
	Postural sway	Lucchini et al., 2014 CATSYS Postural Sway, (Sway 2) Sway velocity (mm/s)	Postural sway	0.080	0.132	0.017	-0.179	0.339	0.603	0.546
Fixed	Postural sway			-0.060	0.028	0.001	-0.116	-0.005	-2.128	0.033
Random	Postural sway			-0.061	0.029	0.001	-0.117	-0.004	-2.102	0.036
	Reaction time	Lucchini et al., 2014 Simple Visual Reaction Time, Mean (miliseconds)	Reaction time	-0.101	0.129	0.017	-0.355	0.152	-0.784	0.433
	Reaction time	Lucchini et al., 2014 Simple Visual Reaction Time, Std Dev	Reaction time	-0.180	0.129	0.017	-0.433	0.074	-1.390	0.165
Fixed	Reaction time			-0.141	0.091	0.008	-0.320	0.039	-1.537	0.124
Random	Reaction time			-0.141	0.091	0.008	-0.320	0.039	-1.537	0.124
	Self-directed manual speed	Bowler et al., 2012 Finger Tapping dom t-score	Self-directed manual speed	0.034	0.145	0.021	-0.251	0.318	0.232	0.817
	Self-directed manual speed	Bowler et al., 2012 Finger Tapping non-dom t-score	Self-directed manual speed	-0.019	0.145	0.021	-0.304	0.266	-0.130	0.897
	Self-directed manual speed	Lucchini et al., 2014 Finger Tapping dom	Self-directed manual speed	-0.171	0.129	0.017	-0.425	0.082	-1.323	0.186
	Self-directed manual speed	Lucchini et al., 2014 Finger Tapping non-dom	Self-directed manual speed	-0.112	0.129	0.017	-0.366	0.141	-0.870	0.385
Fixed	Self-directed manual speed			-0.076	0.068	0.005	-0.210	0.058	-1.110	0.267
Random	Self-directed manual speed			-0.076	0.068	0.005	-0.210	0.058	-1.110	0.267
	Tremor	Lucchini et al., 2014 CATSYS Tremor, Tremor Intensity Right Hand (m/s2)	Tremor	0.149	0.132	0.018	-0.111	0.408	1.123	0.261
	Tremor	Lucchini et al., 2014 CATSYS Tremor, Centre Frequency Right Hand (Hz)	Tremor	-0.031	0.132	0.017	-0.290	0.228	-0.234	0.815
	Tremor	Lucchini et al., 2014 CATSYS Tremor, Frequency dispersion Right Hand (Hz)	Tremor	0.083	0.132	0.017	-0.176	0.342	0.626	0.531
	Tremor	Lucchini et al., 2014 CATSYS Tremor Harmonic index Right Hand	Tremor	-0.068	0.132	0.017	-0.327	0.191	-0.516	0.606
	Tremor	Lucchini et al., 2014 CATSYS Tremor, Tremor Intensity Left Hand (m/s2)	Tremor	0.119	0.132	0.018	-0.141	0.378	0.896	0.370
	Tremor	Lucchini et al., 2014 CATSYS Tremor, Centre Frequency Left Hand (Hz)	Tremor	0.098	0.132	0.017	-0.162	0.357	0.737	0.461
	Tremor	Lucchini et al., 2014 CATSYS Tremor, Frequency dispersion Leftt Hand (Hz)	Tremor	0.106	0.132	0.018	-0.153	0.365	0.801	0.423
	Tremor	Lucchini et al., 2014 CATSYSTremor, Harmonic index Left Hand	Tremor	-0.087	0.132	0.017	-0.346	0.172	-0.658	0.511
Fixed	Tremor			0.046	0.047	0.002	-0.046	0.138	0.980	0.327
Random	Tremor			0.046	0.047	0.002	-0.046	0.138	0.980	0.327
Fixed	Overall			-0.112	0.018	0.000	-0.147	-0.077	-6.270	0.000
Random	Overall			-0.113	0.018	0.000	-0.149	-0.077	-6.131	0.000

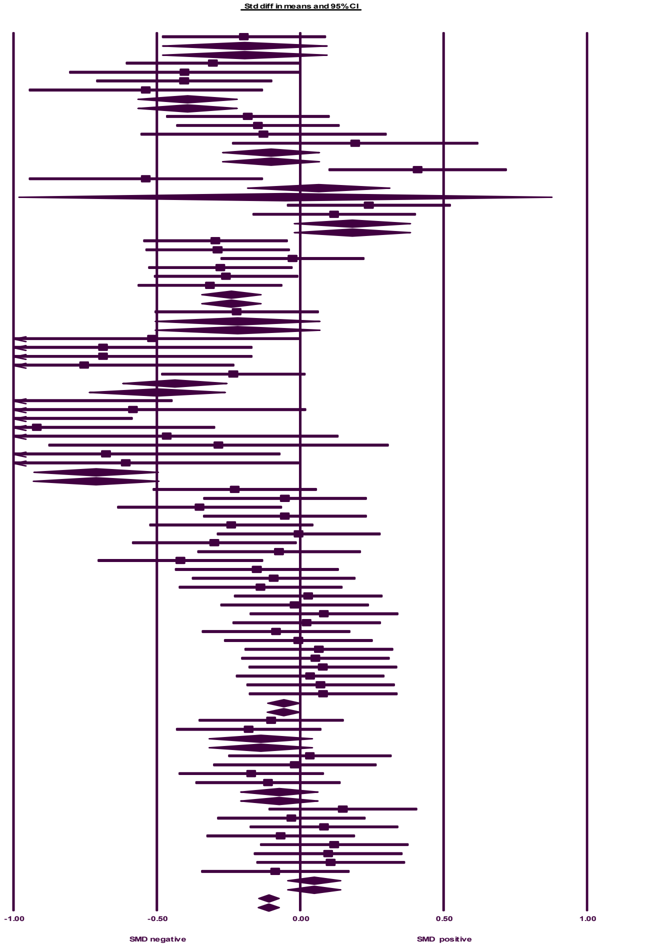

Online supplementary Figure S13. *Motor function. Standardized mean differences (SMDs) between groups, as a function of the motor domain assessed. Note: a negative SMD indicates that the group with higher Mn levels had worse motor function on average. A random effects model is used to combine studies within each subgroup. A fixed effect model is used to combine subgroups and yield the overall effect. The study-to-study variance (tau-squared) is NOT assumed to be the same for all subgroups - this value is computed within subgroups and NOT pooled across subgroups.*

Model	Group by Subgroup within study	Study name	Subgroup within study	Std diff in means	Standard error	Variance	Lower limit	Upper limit	Z-Value	p-Value
	Air Mn & Blood	Bowler et al., 2012 Finger Tapping dom t-score	Air Mn & Blood	0.034	0.145	0.021	-0.251	0.318	0.232	0.817
	Air Mn & Blood	Bowler et al., 2012 Finger Tapping non-dom t-score	Air Mn & Blood	-0.019	0.145	0.021	-0.304	0.266	-0.130	0.897
	Air Mn & Blood	Bowler et al., 2012 Dynamometer dom t-score	Air Mn & Blood	0.239	0.146	0.021	-0.046	0.525	1.642	0.101
	Air Mn & Blood	Bowler et al., 2012 Dynamometer non-dom t-score	Air Mn & Blood	0.118	0.145	0.021	-0.167	0.403	0.814	0.415
	Air Mn & Blood	Bowler et al., 2012 Grooved Pegboard dom t score	Air Mn & Blood	-0.183	0.146	0.021	-0.468	0.103	-1.255	0.209
	Air Mn & Blood	Bowler et al., 2012 Grooved Pegboard non-dom t-score	Air Mn & Blood	-0.148	0.145	0.021	-0.433	0.138	-1.015	0.310
	Air Mn & Blood	Bowler et al., 2012 Unified Parkinson's Disease Rating Scale (UPDRS), Body bradykinesia	Air Mn & Blood	-0.196	0.146	0.021	-0.483	0.090	-1.345	0.179
	Air Mn & Blood	Bowler et al., 2012 UPDRS, Motor	Air Mn & Blood	-0.221	0.146	0.021	-0.508	0.065	-1.516	0.130
	Air Mn & Blood	Kim et al., 2011 Computerized Adaptive Testing System (CATSYS) Postural Sway, (Sway 1) Mean sway (mm)	Air Mn & Blood	-0.228	0.146	0.021	-0.515	0.058	-1.562	0.118
	Air Mn & Blood	Kim et al., 2011 CATSYS Postural Sway, (Sway 1) Transversal sway (mm)	Air Mn & Blood	-0.053	0.146	0.021	-0.339	0.232	-0.366	0.714
	Air Mn & Blood	Kim et al., 2011 CATSYS Postural Sway, (Sway 1) Sagittal sway (mm)	Air Mn & Blood	-0.351	0.147	0.022	-0.639	-0.063	-2.392	0.017
	Air Mn & Blood	Kim et al., 2011 CATSYS Postural Sway, (Sway 1) Sway area (mm2)	Air Mn & Blood	-0.054	0.146	0.021	-0.339	0.232	-0.369	0.712
	Air Mn & Blood	Kim et al., 2011 CATSYS Postural Sway, (Sway 1) Sway intensity	Air Mn & Blood	-0.240	0.146	0.021	-0.527	0.046	-1.645	0.100
	Air Mn & Blood	Kim et al., 2011 CATSYS Postural Sway, (Sway 1) Sway velocity (mm/s)	Air Mn & Blood	-0.006	0.146	0.021	-0.291	0.280	-0.039	0.969
	Air Mn & Blood	Kim et al., 2011 CATSYS Postural Sway, (Sway 3) Mean sway (mm)	Air Mn & Blood	-0.299	0.146	0.021	-0.586	-0.012	-2.044	0.041
	Air Mn & Blood	Kim et al., 2011 CATSYS Postural Sway, (Sway 3) Transversal sway (mm)	Air Mn & Blood	-0.074	0.146	0.021	-0.360	0.212	-0.508	0.612
	Air Mn & Blood	Kim et al., 2011 CATSYS Postural Sway, (Sway 3) Sagittal sway (mm)	Air Mn & Blood	-0.418	0.147	0.022	-0.706	-0.129	-2.839	0.005
	Air Mn & Blood	Kim et al., 2011 CATSYS Postural Sway, (Sway 3) Sway area (mm2)	Air Mn & Blood	-0.151	0.146	0.021	-0.437	0.135	-1.037	0.300
	Air Mn & Blood	Kim et al., 2011 CATSYS Postural Sway, (Sway 3) Sway intensity	Air Mn & Blood	-0.093	0.146	0.021	-0.378	0.193	-0.636	0.525
	Air Mn & Blood	Kim et al., 2011 CATSYS Postural Sway, (Sway 3) Sway velocity (mm/s)	Air Mn & Blood	-0.138	0.146	0.021	-0.424	0.148	-0.948	0.343
Fixed	Air Mn & Blood			-0.123	0.033	0.001	-0.187	-0.059	-3.780	0.000
Random	Air Mn & Blood			-0.123	0.035	0.001	-0.192	-0.055	-3.527	0.000
	Air Mn, Blood & Urine	Lucchini et al., 2014 Finger Tapping dom	Air Mn, Blood & Urine	-0.171	0.129	0.017	-0.425	0.082	-1.323	0.186
	Air Mn, Blood & Urine	Lucchini et al., 2014 Finger Tapping non-dom	Air Mn, Blood & Urine	-0.112	0.129	0.017	-0.366	0.141	-0.870	0.385
	Air Mn, Blood & Urine	Lucchini et al., 2014 Sniffin' Sticks test, identification	Air Mn, Blood & Urine	-0.233	0.129	0.017	-0.485	0.019	-1.815	0.070
	Air Mn, Blood & Urine	Lucchini et al., 2014 CATSYS Postural Sway, (Sway 1) Mean Sway (mm)	Air Mn, Blood & Urine	0.028	0.132	0.017	-0.232	0.287	0.208	0.835
	Air Mn, Blood & Urine	Lucchini et al., 2014 CATSYS Postural Sway, (Sway 1) Transversal sway (mm)	Air Mn, Blood & Urine	-0.020	0.132	0.017	-0.279	0.240	-0.148	0.882
	Air Mn, Blood & Urine	Lucchini et al., 2014 CATSYS Postural Sway, (Sway 1) Sagittal sway (mm)	Air Mn, Blood & Urine	0.083	0.132	0.017	-0.177	0.342	0.624	0.533
	Air Mn, Blood & Urine	Lucchini et al., 2014 CATSYS Postural Sway, (Sway 1) Sway area (mm2)	Air Mn, Blood & Urine	0.022	0.132	0.017	-0.237	0.281	0.169	0.866
	Air Mn, Blood & Urine	Lucchini et al., 2014 CATSYS Postural Sway, (Sway 1) Sway intensity	Air Mn, Blood & Urine	-0.084	0.132	0.017	-0.344	0.175	-0.637	0.524
	Air Mn, Blood & Urine	Lucchini et al., 2014 CATSYS Postural Sway, (Sway 1) Sway velocity (mm/s)	Air Mn, Blood & Urine	-0.007	0.132	0.017	-0.266	0.252	-0.051	0.960
	Air Mn, Blood & Urine	Lucchini et al., 2014 CATSYS Postural Sway, (Sway 2) Mean Sway (mm) assesment EC	Air Mn, Blood & Urine	0.065	0.132	0.017	-0.194	0.324	0.490	0.624
	Air Mn, Blood & Urine	Lucchini et al., 2014 CATSYS Postural Sway, (Sway 2) Transversal sway (mm)	Air Mn, Blood & Urine	0.053	0.132	0.017	-0.206	0.312	0.401	0.689
	Air Mn, Blood & Urine	Lucchini et al., 2014 CATSYS Postural Sway, (Sway 2) Sagittal sway (mm)	Air Mn, Blood & Urine	0.079	0.132	0.017	-0.181	0.338	0.594	0.552
	Air Mn, Blood & Urine	Lucchini et al., 2014 CATSYS Postural Sway, (Sway 2) Sway area (mm2)	Air Mn, Blood & Urine	0.035	0.132	0.017	-0.225	0.294	0.261	0.794
	Air Mn, Blood & Urine	Lucchini et al., 2014 CATSYS Postural Sway, (Sway 2) Sway intensity	Air Mn, Blood & Urine	0.071	0.132	0.017	-0.188	0.330	0.537	0.591
	Air Mn, Blood & Urine	Lucchini et al., 2014 CATSYS Postural Sway, (Sway 2) Sway velocity (mm/s)	Air Mn, Blood & Urine	0.080	0.132	0.017	-0.179	0.339	0.603	0.546
	Air Mn, Blood & Urine	Lucchini et al., 2014 CATSYS Tremor, Tremor Intensity Right Hand (m/s2)	Air Mn, Blood & Urine	0.149	0.132	0.018	-0.111	0.408	1.123	0.261
	Air Mn, Blood & Urine	Lucchini et al., 2014 CATSYS Tremor, Centre Frequency Right Hand (Hz)	Air Mn, Blood & Urine	-0.031	0.132	0.017	-0.290	0.228	-0.234	0.815
	Air Mn, Blood & Urine	Lucchini et al., 2014 CATSYS Tremor, Frequency dispersion Right Hand (Hz)	Air Mn, Blood & Urine	0.083	0.132	0.017	-0.176	0.342	0.626	0.531
	Air Mn, Blood & Urine	Lucchini et al., 2014 CATSYS Tremor Harmonic index Right Hand	Air Mn, Blood & Urine	-0.068	0.132	0.017	-0.327	0.191	-0.516	0.606
	Air Mn, Blood & Urine	Lucchini et al., 2014 CATSYS Tremor, Tremor Intensity Left Hand (m/s2)	Air Mn, Blood & Urine	0.119	0.132	0.018	-0.141	0.378	0.896	0.370
	Air Mn, Blood & Urine	Lucchini et al., 2014 CATSYS Tremor, Centre Frequency Left Hand (Hz)	Air Mn, Blood & Urine	0.098	0.132	0.017	-0.162	0.357	0.737	0.461
	Air Mn, Blood & Urine	Lucchini et al., 2014 CATSYS Tremor, Frequency dispersion Leftt Hand (Hz)	Air Mn, Blood & Urine	0.106	0.132	0.018	-0.153	0.365	0.801	0.423
	Air Mn, Blood & Urine	Lucchini et al., 2014 CATSYSTremor, Harmonic index Left Hand	Air Mn, Blood & Urine	-0.087	0.132	0.017	-0.346	0.172	-0.658	0.511
	Air Mn, Blood & Urine	Lucchini et al., 2014 Luria Nebraska, Task 1	Air Mn, Blood & Urine	-0.296	0.129	0.017	-0.548	-0.044	-2.299	0.022
	Air Mn, Blood & Urine	Lucchini et al., 2014 Luria Nebraska, Task 2	Air Mn, Blood & Urine	-0.288	0.129	0.017	-0.540	-0.036	-2.240	0.025
	Air Mn, Blood & Urine	Lucchini et al., 2014 Luria Nebraska, Task 3	Air Mn, Blood & Urine	-0.027	0.128	0.016	-0.278	0.224	-0.209	0.834
	Air Mn, Blood & Urine	Lucchini et al., 2014 Luria Nebraska, Task 4	Air Mn, Blood & Urine	-0.278	0.129	0.017	-0.530	-0.026	-2.165	0.030
	Air Mn, Blood & Urine	Lucchini et al., 2014 Luria Nebraska, Task 5	Air Mn, Blood & Urine	-0.259	0.129	0.017	-0.511	-0.007	-2.014	0.044
	Air Mn, Blood & Urine	Lucchini et al., 2014 Luria Nebraska, sum score of the five subtests	Air Mn, Blood & Urine	-0.315	0.129	0.017	-0.567	-0.063	-2.447	0.014
	Air Mn, Blood & Urine	Lucchini et al., 2014 Simple Visual Reaction Time, Mean (miliseconds)	Air Mn, Blood & Urine	-0.101	0.129	0.017	-0.355	0.152	-0.784	0.433
	Air Mn, Blood & Urine	Lucchini et al., 2014 Simple Visual Reaction Time, Std Dev	Air Mn, Blood & Urine	-0.180	0.129	0.017	-0.433	0.074	-1.390	0.165
Fixed	Air Mn, Blood & Urine			-0.051	0.024	0.001	-0.097	-0.005	-2.171	0.030
Random	Air Mn, Blood & Urine			-0.051	0.026	0.001	-0.101	-0.000	-1.978	0.048
	Blood	Mergler et al., 1999 Eurythmokinesimeter (EKM) Irregularity (<50 years)	Blood	-0.305	0.155	0.024	-0.609	-0.000	-1.963	0.050
	Blood	Mergler et al., 1999 EKM Irregularity (>50 years)	Blood	-0.404	0.205	0.042	-0.806	-0.001	-1.965	0.049
	Blood	Mergler et al., 1999 EKM Fitts´ constant (<50years)	Blood	-0.405	0.157	0.025	-0.712	-0.097	-2.580	0.010
	Blood	Mergler et al., 1999 EKM Fitts´constant (>50 years)	Blood	-0.538	0.208	0.043	-0.947	-0.130	-2.583	0.010
	Blood	Mergler et al., 1999 Purdue Pegboard (<50 years)	Blood	0.410	0.159	0.025	0.098	0.721	2.580	0.010
	Blood	Mergler et al., 1999 Purdue Pegboard (>50 years)	Blood	-0.538	0.208	0.043	-0.947	-0.130	-2.583	0.010
Fixed	Blood			-0.246	0.072	0.005	-0.388	-0.105	-3.411	0.001
Random	Blood			-0.285	0.156	0.024	-0.590	0.021	-1.825	0.068
	Blood & Hair	Standridge et al., 2008 Postural balance testing (eyes open on the platform), Ln Sway area (cm2)	Blood & Hair	-1.078	0.323	0.104	-1.710	-0.445	-3.340	0.001
	Blood & Hair	Standridge et al., 2008 Postural balance testing (eyes closed on the platform), Ln Sway area (cm2)	Blood & Hair	-0.583	0.308	0.095	-1.186	0.021	-1.893	0.058
	Blood & Hair	Standridge et al., 2008 Postural balance testing (eyes open on a 4 inch thick foam pad), Ln Sway area (cm2)	Blood & Hair	-1.229	0.329	0.108	-1.873	-0.584	-3.737	0.000
	Blood & Hair	Standridge et al., 2008 Postural balance testing (eyes closed on a 4 inch thick foam pad), Ln Sway area (cm2)	Blood & Hair	-0.918	0.317	0.100	-1.540	-0.297	-2.897	0.004
	Blood & Hair	Standridge et al., 2008 Postural balance testing (eyes open on the platform), Ln Sway length (cm)	Blood & Hair	-0.466	0.306	0.093	-1.065	0.133	-1.524	0.128
	Blood & Hair	Standridge et al., 2008 Postural balance testing (eyes closed on the platform), Ln Sway length (cm)	Blood & Hair	-0.285	0.303	0.092	-0.879	0.309	-0.940	0.347
	Blood & Hair	Standridge et al., 2008 Postural balance testing (eyes open on a 4 inch thick foam pad), Ln Sway lenght (cm)	Blood & Hair	-0.677	0.310	0.096	-1.285	-0.070	-2.185	0.029
	Blood & Hair	Standridge et al ., 2008 Postural balance testing (eyes closed on a 4 inch thick foam pad), Ln Sway lenght (cm)	Blood & Hair	-0.608	0.308	0.095	-1.213	-0.004	-1.973	0.049
Fixed	Blood & Hair			-0.715	0.111	0.012	-0.932	-0.498	-6.470	0.000
Random	Blood & Hair			-0.715	0.111	0.012	-0.934	-0.497	-6.420	0.000
	Hair	Guarneros et al., 2013 Sniffin' sticks test, threshold	Hair	-0.517	0.262	0.069	-1.031	-0.002	-1.969	0.049
	Hair	Guarneros et al., 2013 Sniffin' sticks test, discrimination	Hair	-0.688	0.266	0.071	-1.208	-0.167	-2.588	0.010
	Hair	Guarneros et al., 2013 Sniffin' sticks test, identification	Hair	-0.688	0.266	0.071	-1.208	-0.167	-2.588	0.010
	Hair	Guarneros et al., 2013 Sniffin' sticks test, overall performance	Hair	-0.754	0.267	0.071	-1.277	-0.230	-2.820	0.005
Fixed	Hair			-0.660	0.133	0.018	-0.920	-0.400	-4.978	0.000
Random	Hair			-0.660	0.133	0.018	-0.920	-0.400	-4.978	0.000
	Scalp hair, Axilary Hair, Fingernails & Saliva	Viana et al., 2014 Grooved Pegboard dom (seconds)	Scalp hair, Axilary Hair, Fingernails & Saliva	-0.128	0.219	0.048	-0.557	0.301	-0.585	0.559
	Scalp hair, Axilary Hair, Fingernails & Saliva	Viana et al., 2014 Grooved Pegboard non-dom (seconds)	Scalp hair, Axilary Hair, Fingernails & Saliva	0.191	0.219	0.048	-0.238	0.621	0.873	0.383
Fixed	Scalp hair, Axilary Hair, Fingernails & Saliva			0.032	0.155	0.024	-0.272	0.335	0.203	0.839
Random	Scalp hair, Axilary Hair, Fingernails & Saliva			0.032	0.160	0.026	-0.282	0.345	0.197	0.844
Fixed	Overall			-0.112	0.018	0.000	-0.147	-0.077	-6.270	0.000
Random	Overall			-0.111	0.020	0.000	-0.149	-0.072	-5.605	0.000

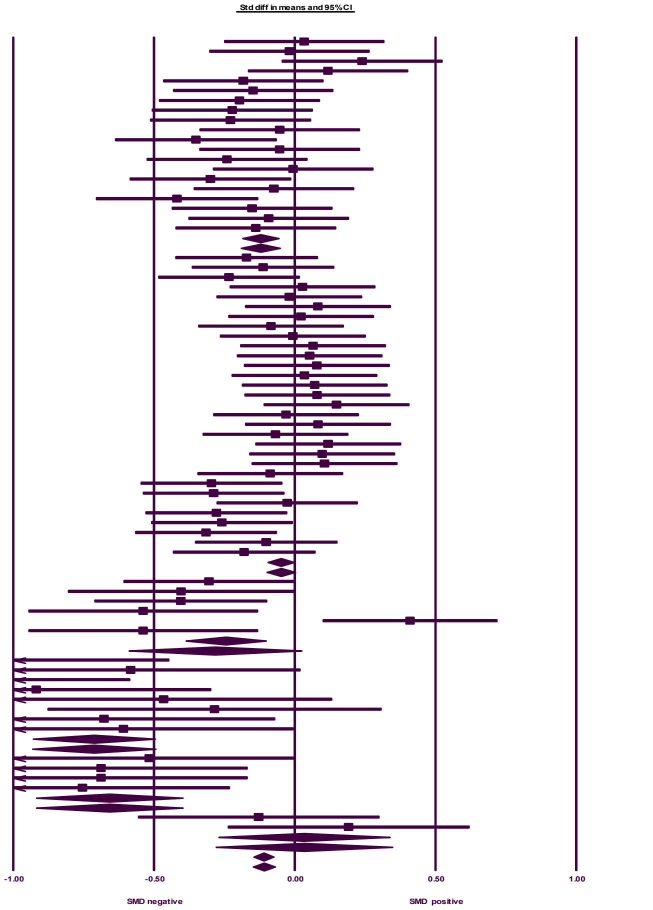

Online supplementary Figure 14. *Motor function. Standardized mean differences (SMDs) between groups, as a function of the type of exposure to Mn evaluated. Note: a negative SMD indicates that the group with higher Mn levels had worse motor function on average. A random effects model is used to combine studies within each subgroup. A fixed effect model is used to combine subgroups and yield the overall effect. The study-to-study variance (tau-squared) is NOT assumed to be the same for all subgroups - this value is computed within subgroups and NOT pooled across subgroups.*

Online supplementary appendices

Online supplementary appendix 1.1. *Studies based on the same geographical areas or exposed population.*

Beuter et al. (1999), is the same study as Mergler et al. (1999) based on 273 healthy persons (without cognitive impairment) from Southwest Quebec (Canada) where a former Mn alloy production plant existed [30,31]. Mergler et al. was the only one reporting mean differences susceptible of meta-analysis. Anyway, in Beuter et al. study, exposure to Mn was also found to be associated with a decrease in ability to perform regular, rapid and precise pointing movements and a decrease in ability to attain high maximum rotation speeds in rapid alternated movement, and an increase in regularity of tremor oscillations.

In Bowler et al. (2012), the population of Marietta (n=100) is compared to that of Mount Vernon, Knox County, Ohio (n=90), which is considered unexposed [37]. Kim et al. (2011) is the same population study as Bowler et al. (2012) [36]. In Bowler et al. (2015 and 2016), the population of Marietta is compared with that of East Liverpool (n=86), Ohio, also highly exposed to environmental Mn from industrial sources [28,42]. The publications by Bowler et al. in 2015 and 2016 are therefore the same study, but the first presents the results for cognitive function and the last for motor function. Kornblith et al. (2018) study [45] is also the same population study as Bowler et al. 2015 and 2016, but with different analysis strategy focused on different objectives, as this study aimed to determine whether: 1) clusters or subtypes of Tremor and No Tremor symptoms exist in Mn neurotoxicity as they do in Parkinson's disease (PD), and 2) to the extent subtypes exist, whether the clustering of No Tremor symptoms and executive functioning (EF) impairment resembles the clustering seen in samples of PD patients. The study published by Standridge et al. (2008), also includes healthy (non-cognitively impaired) individuals aged 19-68 (mean 50) from Marietta, Ohio [34].

From 2011 to 2013, four published articles were identified [3,32,35,39] based on the central Mexican state of Hidalgo, where the most important Mn extraction and processing facilities of Mexico are located. In Santos Burgoa et al. (2001) study, cognitive function was finally performed in 44 subjects and 27 subjects from two communities respectively which names were not detailed [32]. Rodríguez-Agudelo et al. (2006) and Solís Vivanco et al. (2009) present data from the same population study; 288 people (168 women and 120 men) from eight communities at various distances from Mn extraction or processing facilities in the district of Molango; but focusing on motor and cognitive function respectively [33,35]. In the Guarneros et al. (2013) study, 30 subjects living <1 km from the Mn processing plant (Tolago/Chiconcoac), were compared to other 30 non-exposed subjects living 50 km from the closest source of exposure [39].

Online supplementary appendix 1.2. *Studies included in the systematic review but not in any analysis strategy of the meta-analysis.*

As mentioned above, Beuter et al. (1999), is the same study as Mergler et al, (1999) but without reporting data susceptible of meta-analysis [30,31].

Santos Burgoa et al. (2001), using multivariate logistic regression, reported an adjusted 11.70 fold-increase in the risk of deficient cognitive performance (Mini-Mental score of less than 17) in subjects with higher levels of Blood Mn (when blood Mn was above the 75th percentile, in comparison to the lowest population 25th percentile level). No data susceptible of being included in our meta-analysis was reported [32].

In the Rodríguez-Agudelo et al. (2006) study, considering cumulative exposure index in quartiles (to have variability below and above the cutting point), there was an association between air Mn concentrations and motor tests that assessed the coordination of two movements: Odds Ratio (OR)= 3.69; 95%CI (0.9 to 15.13) and position changes in hand movements, with statistical significance: OR= 3.09; (95%CI 1.07 to 8.92). An association with tests evaluating conflictive reactions (task that explores verbal regulations of movements) was also found: OR= 2.30; 95%CI (1.00 to 5.28). No association was found between blood Mn and poorer motor tests results. No data susceptible of being included in our meta-analysis was reported [33].

In the Solís Vivanco et al. (2009) study, when using the 0.1 μg/m^3 cut-off point, there was a statistically significant association between air Mn and poor performance on the digit span test (attention impairment): OR= 1.75; 95%CI (1.01 to 3.06). When using the 0.05 μg/m^3 cut-off point there was no risk of poor performance on any test (e.g. OR digit span test= 1.24; 95%CI (0.67 to 2.29). There was no statistically significant association between blood Mn concentrations and cognitive function (e.g. OR MMSE= 1.17, 95%CI (0.99 to 1.38) [35].

The study by Kornblith et al (2018) is also the same study as that by Bowler et al, 2015 and 2016, but as it was based on the identification of clusters, it also did not report any data that could be meta-analysed [45].

Cabral Pinto et al. (2018) study determined potentially toxic elements (PTEs) concentrations in Urine including Mn exposure in103 permanent residents from the industrial city of Estarreja (Portugal), >55 years old, where only 40.2% of the subjects had a normal performance on neurological tests assessing cognitive status. 18.3% showed a mild cognitive impairment compatible (MCI) and the rest (36.6%) had a cognitive performance suggestive of dementia condition. Their linear regression models showed that aluminium (R^2 = 38%), cadmium (R^2 = 11%) and zinc (R^2 = 6%) were good predictors of the scores of the MMSE cognitive test, but Mn was not showed as a good predictor. Specific R^2 result for Mn is not reported [44].

The article published by Rafiee et al.(2019) also met inclusion criteria for our meta-analysis, as it assessed the effects of exposure to heavy metals including Mn determined through hair obtained by volunteers in hairdressing salons (in their usual haircuts), on cognitive function in the domains of attention and executive function determined through TMT* (A and B). Finally, their results could not be included in any of our final analyses, because they reported no correlation and no difference in means between groups. Their analysis strategy was based on a difference in TMT* scores for each unit increase in exposure, determined through the additive coefficient (ß) obtained through a linear regression model. Mn levels in hair were significantly associated with 0.201 and 0.204 more seconds per one 1 μg/g of Mn in TMT*-A and B score respectively, supporting the hypothesis that the higher the exposure, the worse the cognitive function [46].

Online supplementary appendix 1.3. *Studies included in some meta-analysis strategy, but which also provided other results that could not be meta-analysed.*

Kim et al. (2011) in US, reported also motor function data subsidiary to be analyzed through a SMD in our meta-analysis. In addition, they reported positive Odds Ratios ORs between abnormal Unified Parkinson's Disease Rating Scale (UPDRS) findings (scores > 0) using ''Motor and Bradykinesia'' criteria, with respect to study group (exposed vs. comparison town), after adjustment for covariates. Regarding correlation analysis, they showed that there was not a significant correlation between Mn-B or modeled air-Mn levels and any of the seven postural sway parameters measured using the CATSYS 2000 system (Coordination Ability Test System, Danish Product Development), but data were not shown so it was not possible to include these data in the motor function & correlation meta-analysis approach [36].

Guarneros et al. (2013) in Mexico, reported motor function data subsidiary to be analyzed through a SMD in our meta-analysis. In addition, they reported an association between Mn exposure and worse motor function in the form of Odds Ratios (ORs). The median level of hair Mn (9.73 μg/g) in subjects living <1 km from a Mn processing plant were 9-fold higher (OR=9) than median levels (1.01 μg/g) in non-exposed subjects living 50 km from the closest source of exposure, p<0.001. A tendential negative correlation was found between hair Mn and the performance of subjects within each group on Sniffin' sticks 3 subtests battery. Although this was clearer for the exposed than for the control subjects, none of the correlations reached significance for either group. Specific data were not shown so it was not possible to include these data in the Motor Function & correlation meta-analysis approach [39].

Iqbal et al. (2018) in Pakistan, reported cognitive function data subsidiary to be analyzed through a correlation coefficient in our meta-analysis. In addition to determining correlations, they compared

the means of Mn blood levels in 4 groups ordinarily classified based on MMSE scores. These data could not be included in the approach to SMD and cognitive function, but their results showed that mean Blood Mn levels were significantly higher in each of the cognitively impaired patients ordinal categorized according to their MMSE scores [43].
